# Quantitative Analysis Reveals that Actin and Src-Family Kinases Regulate Nuclear YAP1 and Its Export

**DOI:** 10.1016/j.cels.2018.05.006

**Published:** 2018-06-27

**Authors:** Nil Ege, Anna M. Dowbaj, Ming Jiang, Michael Howell, Steven Hooper, Charles Foster, Robert P. Jenkins, Erik Sahai

**Affiliations:** 1 Tumour Cell Biology Laboratory, The Francis Crick Institute, London NW1 1AT, UK; 2 Cell and Developmental Biology Department, University College London, London WC1E 6BT, UK; 3 High Throughput Screening, The Francis Crick Institute, London NW1 1AT, UK; 4 Transcription Laboratory, The Francis Crick Institute, London NW1 1AT, UK

**Keywords:** YAP1, fibroblast, mathematical modeling, photobleaching, nuclear export, actomyosin cytoskeleton, Src-family kinases

## Abstract

The transcriptional regulator YAP1 is critical for the pathological activation of fibroblasts. In normal fibroblasts, YAP1 is located in the cytoplasm, while in activated cancer-associated fibroblasts, it is nuclear and promotes the expression of genes required for pro-tumorigenic functions. Here, we investigate the dynamics of YAP1 shuttling in normal and activated fibroblasts, using EYFP-YAP1, quantitative photobleaching methods, and mathematical modeling. Imaging of migrating fibroblasts reveals the tight temporal coupling of cell shape change and altered YAP1 localization. Both 14-3-3 and TEAD binding modulate YAP1 shuttling, but neither affects nuclear import. Instead, we find that YAP1 nuclear accumulation in activated fibroblasts results from Src and actomyosin-dependent suppression of phosphorylated YAP1 export. Finally, we show that nuclear-constrained YAP1, upon XPO1 depletion, remains sensitive to blockade of actomyosin function. Together, these data place nuclear export at the center of YAP1 regulation and indicate that the cytoskeleton can regulate YAP1 within the nucleus.

## Highlights

• Photobleaching coupled with mathematical modeling identifies YAP1 dynamics
• Regulation of nuclear export is key determinant of YAP1 localization
• Serine phosphorylation is required for YAP1 nuclear export through XPO1
• Nuclear YAP1 remains sensitive to actin and Src-family kinase regulation

## Graphical Abstract

**Figure:**
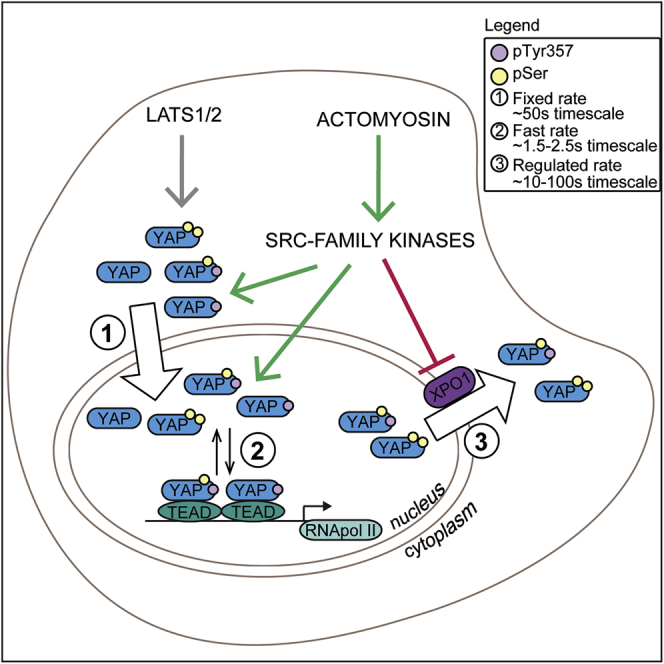

## Teaser

Ege et al. use quantitative analysis of photobleaching experiments to reveal that nuclear export is a convergent point of YAP1 regulation by actomyosin, Src-family kinases, and LATS1/2. Further, actomyosin and Src-family kinases additionally regulate YAP1 transcription competence via tyrosine phosphorylation.

## Introduction

The transmission of signals from the cytoplasm to the transcriptional machinery in the nucleus can occur in many ways. Signal transducing kinases can enter the nucleus and modulate transcription factor activity (Taagepera et al., 1998). Alternatively, DNA binding transcription factors can shuttle between the cytoplasm and the nucleus (Nicolás et al., 2004, Vartiainen et al., 2007, Xu and Massague, 2004). YAP1 and TAZ (WWTR1) are transcriptional regulators that are believed to be sequestered in the cytoplasm via interaction with 14-3-3 proteins when phosphorylated. In the absence of phosphorylation, YAP1 and TAZ are released and can interact with transcription factors, such as TEADs, in the nucleus (Piccolo et al., 2014, Zhao et al., 2011). Structural studies have shown that the YAP1/TEADs interaction is critically dependent on serine 94 in YAP1 (Chen et al., 2010, Li et al., 2010, Zhao et al., 2008). YAP1 and TAZ are negatively regulated by serine phosphorylation by the LATS1/2 kinases, which are themselves regulated by the MST1/2 kinases (Chan et al., 2005, Dong et al., 2007, Hao et al., 2008, Lei et al., 2008, Oka et al., 2008, Zhao et al., 2007). This pathway, often called the Hippo pathway, is critical for controlling the extent of tissue growth and organ size (Dong et al., 2007, Halder and Johnson, 2011, Pan, 2010, Zhao et al., 2011). It is regulated by a network of epithelial junctional molecules that transmit information about tissue integrity. Further, regulation by glucagon and other soluble factors couples tissue growth to nutrient availability (Enzo et al., 2015, Santinon et al., 2016, Yu et al., 2012). In all these cases, the activity of YAP1 and TAZ is negatively regulated by direct LATS1/2-mediated serine phosphorylation on several serine residues, including serine 127 in YAP1 (Zhao et al., 2007). Low levels of YAP1 and TAZ phosphorylation are linked to nuclear accumulation, leading to cell proliferation, wound healing, or tissue regeneration (Camargo et al., 2007, Dong et al., 2007, Gao et al., 2013, Lavado et al., 2013, Schlegelmilch et al., 2011, Zhao et al., 2011). High levels of phosphorylation are linked to cell quiescence via promotion of complexes with 14-3-3 proteins in the cytoplasm (Aitken, 2006, Muslin and Xing, 2000).

Mechanical cues and tyrosine phosphorylation can modulate YAP1 function (Dupont et al., 2011, Li et al., 2016) and are proposed to enable epithelial cells to monitor organ size (Benham-Pyle et al., 2015, Fernandez et al., 2011, Porazinski et al., 2015, Sansores-Garcia et al., 2011). This may depend on Src-mediated phosphorylation of tyrosine 357; however, the full details of how mechanical cues regulate YAP1 are not determined. YAP1 activation in fibroblasts within tumors depends on the actin cytoskeleton and is correlated with increased nuclear YAP1 and Y357 phosphorylation, but S127 phosphorylation and LATS1/2 activity are not changed (Calvo et al., 2013).

Little is known about the dynamics of YAP1 shuttling in and out of the nucleus (Zhao et al., 2007). Many binding partners have been identified, including the TEADs, 14-3-3, and cytoplasmic proteins localized at cell junctions (Couzens et al., 2013, Moya and Halder, 2014), yet it remains unclear if YAP1 is stably sequestered at these sites in either the cytoplasm or the nucleus. The rate of YAP1 shuttling between the cytoplasm and nucleus is not known, and apart from the implication of XPO1 (also called Exportin1 or Crm1) in YAP1 nuclear export (Dupont et al., 2011, Ren et al., 2010, Wei et al., 2015), the machinery regulating YAP1 entry and exit from the nucleus is not known. We answer these questions by using a variety of live imaging methods and mathematical analysis (Nicolás et al., 2004, Vartiainen et al., 2007). Fluorescence recovery after photobleaching (FRAP) is used to provide information about sequestration, diffusion, and the rate of dissociation from TEAD transcription factors. Fluorescence loss in photobleaching (FLIP) is used to assess nuclear import and export rates and the rate of TEAD association. By combining these methods with YAP1 point mutations, actomyosin manipulations, and a screen for regulators of YAP1 nuclear import/export, we are able to derive a detailed model of YAP1 dynamics in normal fibroblasts (NFs) and pathologically activated fibroblasts (CAFs).

## Results

### Establishment of a Functional YAP1 Fluorescent Protein

**Figure 1:**
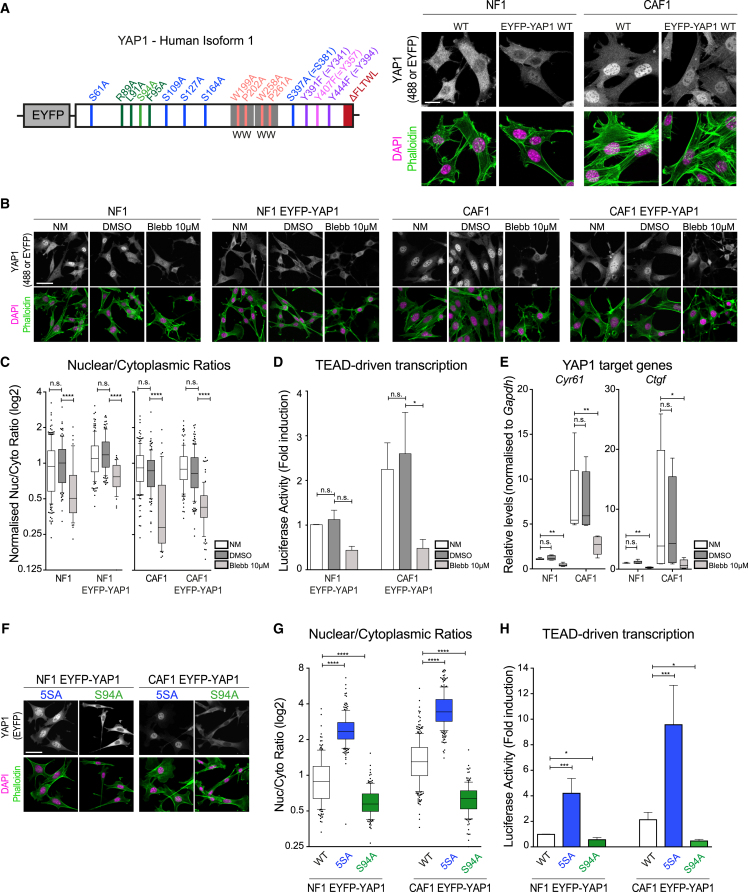
Establishment of a Functional YAP1 Fluorescent Protein (A) Schematic of the EYFP-YAP1 construct used in this study with relevant mutations together with representative images of endogenous YAP1 and EYFP-YAP1 localization in NF1 and CAF1. The isoform used is the human isoform 1-2γ. Double numbering refers to amino acid numbers commonly used in other publications. Scale bar, 20 μm. (B) Representative images of endogenous YAP1 and EYFP-YAP1 localization in NF1 and CAF1 in normal condition (normal media [NM]) and treated with DMSO or 10 μM blebbistatin. Scale bar, 50 μm. (C) Boxplot (with whiskers showing 10 and 90 percentiles) of nuclear-to-cytoplasmic ratio (log2 scale) corresponding to quantification of (B). n > 44 cells for each condition from at least two independent experiments. Data are normalized to NM for each cell line. (D) Luciferase assay of WT or EYFP-YAP1 expressing NF1 and CAF1 in NM and treated with DMSO or 10 μM blebbistatin. Bars represent mean ± SEM of four independent experiments. Data are normalized to NF1 in NM for each cell line. (E) Boxplot (with whiskers showing minimum to maximum) of qRT-PCR of two YAP1 target genes normalized to GAPDH in NF1 and CAF1 cell lines in NM, or treated with DMSO or 10 μM blebbistatin. Data summary of three independent experiments, each with two technical replicates. (F) Representative images of EYFP-YAP1_5SA and EYFP-YAP1_S94A localization in NF1 and CAF1. Scale bar, 50 μm. (G) Boxplot (10 and 90) of nuclear-to-cytoplasmic ratio (log2 scale) corresponding to quantification of (F). n > 150 cells for each condition from at least four independent experiments. (H) Luciferase assay of EYFP-YAP1_5SA and EYFP-YAP1_S94A expressing NF1 and CAF1 in NM or treated with DMSO or 10 μM blebbistatin. Bars represent mean ± SEM of eight independent experiments. Mann-Whitney U test, n.s., nonsignificant. ^∗^p ≤ 0.05^∗∗^, p ≤ 0.01, ^∗∗∗^p ≤ 0.001, ^∗∗∗∗^p ≤ 0.0001. See also Figure S1.

To image the localization and dynamics of YAP1 in normal mammary fibroblasts (NF1) and mammary carcinoma-associated fibroblasts (CAF1), we fused EYFP to the N terminus of the protein and generated NF1 and CAF1 stably expressing levels of EYFP-YAP1 similar to the level of the endogenous YAP1 (Figures 1A and S1A), estimated at ∼130,000 EYFP-YAP1 molecules/cell (Figure S1B). There was no correlation between the level of EYFP-YAP1 and its subcellular distribution, indicating that the system is not sensitive to modest variation in the level of YAP1 (Figure S1H). Functional matrix contraction assays demonstrated that the expression of EYFP-YAP1 in both cells did not erroneously activate them (Figure S1C).

We confirmed that EYFP-YAP1 is regulated in a similar manner to endogenous YAP1. Figure 1B shows that EYFP-YAP1 is more nuclear in CAF1 than NF1, mirroring the difference in endogenous protein localization (Figure S1D). EYFP-YAP1 also showed a similar cytoplasmic shift upon actomyosin blockade using blebbistatin (Figure 1C), and this was accompanied by reduced YAP1-dependent transcription (Figures 1D and 1E; note elevated YAP1 transcriptional activity in CAF1 versus NF1). We further probed the behavior of EYFP-YAP1 by introducing well-characterized mutations at serine 94 (S94A) (Zhao et al., 2008) and serines 61, 109, 127, 164, and 381 (termed 5SA) (Zhao et al., 2007), blocking the interaction of YAP1 with TEAD and 14-3-3, respectively (Figures 1A and S1E). Both mutants showed the expected cytoplasmic and nuclear localization, respectively (Figures 1F and 1G); this pattern was not altered by depletion of the endogenous YAP1 (Figures S1G and S1I). We confirmed that the altered localization of EYFP-YAP1_S94A was due to defective TEAD binding with additional R89A, L91A, and F95A mutations in the TEAD binding domain (Li et al., 2010) and TEAD1-4 siRNA (Figures S1J–S1M). Depletion of LATS1/2 promoted the nuclear accumulation of EYFP-YAP1 (Figures S1L and S1M). These data confirm that EYFP-YAP1 recapitulates key features of YAP1 regulation. The functionality of the EYFP-YAP1 was also evidenced by the increased TEAD reporter activity in cells expressing EYFP-YAP1_5SA (Figure 1H). Cells expressing EYFP-YAP1_S94A showed reduced TEAD reporter and matrix contraction activity, indicating that this construct acts as a dominant negative (Figures 1H and S1F).

### YAP1 Is Not Stably Sequestered in the Cytoplasm or the Nucleus

**Figure 2:**
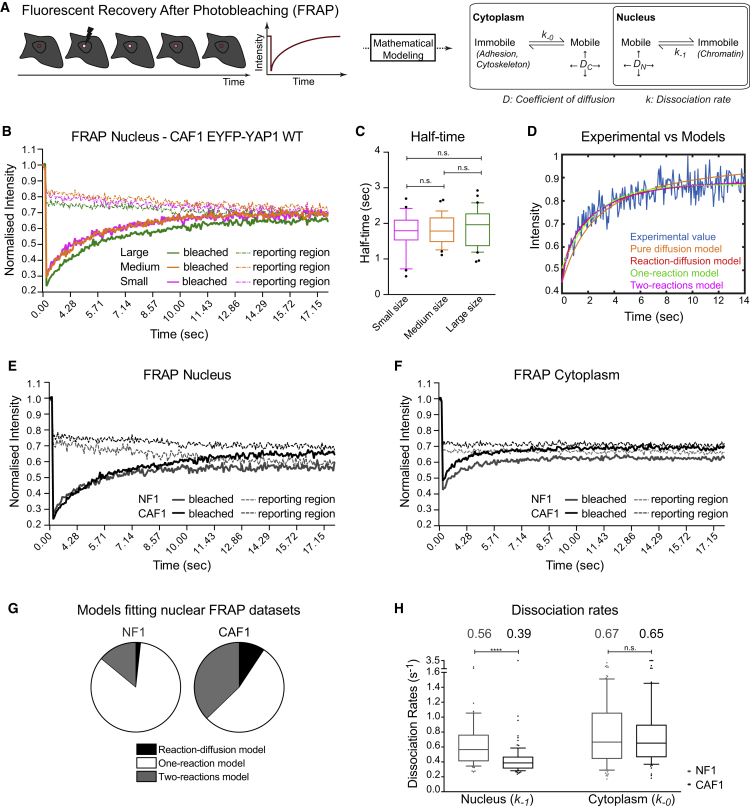
YAP1 Is Not Stably Sequestered in the Cytoplasm or the Nucleus (A) Schematic of the FRAP experiment and parameters extracted with mathematical modeling. (B) Graph showing the median of EYFP-YAP1 intensities of three different-sized bleached (plain line) and reporting (dotted line) regions during nuclear FRAP in CAF1. n > 29 cells for each size. (C) Boxplot (10 and 90) of half-time corresponding to recovery curves from (B). (D) One representative example of the fits of four models to experimental values. (E) Equivalent graph to (B) upon nuclear FRAP in NF1 (n = 20 cells) and CAF1 (n = 30 cells), from three biological replicates. (F) Equivalent graph to (B) upon cytoplasmic FRAP in NF1 (n = 30 cells) and CAF1 (n = 30 cells), from three biological replicates. (G) Pie charts showing the distribution of best-fitting model for nuclear FRAP in NF1 EYFP-YAP1_WT and CAF1 EYFP-YAP1_WT. (H) Boxplot (10 and 90) showing the dissociation rates of EYFP-YAP1 in the nucleus and the cytoplasm of NF1 and CAF1 from three biological replicates. n = 59 (nucleus)/81 (cytoplasm) cells for NF1 and n = 88 (nucleus)/72 (cytoplasm) cells for CAF1. Values above plots indicate medians. Noisy cells are assumed to have rapidly recovered and are represented with a large arbitrary unit of 3.5 s^−1^. Mann-Whitney U test, n.s., nonsignificant. ^∗∗∗∗^p ≤ 0.0001. See also Figures S2 and S3.

Having shown that EYFP-YAP1 is a valid tool to probe YAP1 function, we embarked on FRAP experiments combined with mathematical modeling in order to assess protein diffusion (D_C_ and D_N_) and dissociation rates (k_-0_ and k_-1_) in the cytoplasm and nucleus, respectively (Figure 2A) (Fritzsche and Charras, 2015, Sprague et al., 2004). During FRAP, a region of interest was bleached and the time for fluorescence recovery compared with an adjacent non-bleached region was assessed. An incomplete recovery would indicate an “immobile fraction” sequestered in the compartment of interest. FRAP analyses in both the cytoplasm and the nucleus of NF1 and CAF1 were performed. Figure 2B shows that the bleached area has a fluorescent intensity equivalent to a non-bleached region within ∼15 s (see also Figure S2A). This indicates that there is no measurable “immobile” fraction of EYFP-YAP1 on the timescale of our experiments and thus that the molecule does not engage in permanent binding to a fixed component in the nucleus or in the cytoplasm. We confirmed the validity of our FRAP experiments on fast-diffusing EGFP and stably chromatin-bound H2B-GFP (Figures S2B and S2C; Video S1). The H2B-GFP analysis also demonstrates that chromatin can be considered immobile in the time frame of our experiments.

### Binding to TEAD Transcription Factors Modulates YAP1 Nuclear Mobility

Both diffusion of EYFP-YAP1 and its release from a short-lasting interaction could influence the observed rate of fluorescence recovery (half-time), the effective radius (r_e_) and the depth (K) of the postbleach profile (Figure S2D; Mathematical Methods section of Mathematical Modeling and Model Validation) (Fritzsche and Charras, 2015, Kang et al., 2009, Kang et al., 2010). If diffusion is slow enough, these parameters would change according to the size of the bleached region, as diffusion into a larger region will take longer (Figure S2D, Situation 1). We therefore repeated FRAP analyses with different-sized bleached regions in the nucleus and the cytoplasm. The intensity recoveries (Figure 2B) and the postbleach profile (Figure S3A) showed no differences among the three bleached regions. Furthermore, the half-time, the effective radius, and the bleach depth did not change significantly (Figures 2C and S3). Taken together, these results indicate that diffusion is rapid relative to the imaging rate of 16.7 Hz, suggesting that the recovery observed reflects unbinding/binding reactions (Figure S2D, Situation 2). Consistent with this, Figure 2D shows that a pure diffusion model did not fit the experimental recovery curves well (orange versus blue curves). Both reaction-diffusion (red) and reaction models (green and magenta) fit the data well. Akaike Information Criterion (AIC) analysis indicated that in the majority of cases, reaction-based models fit best (Figure 2G; Table S1; Mathematical Methods section of Mathematical Modeling and Model Validation). In the cases where reaction-diffusion models fit well, the diffusion value was in the range of 25–40 μm^2^s^−1^, which is in the range reported for multimerized GFP with similar molecular mass to EYFP-YAP1 (Baum et al., 2014). Therefore, diffusion is so rapid that it has largely occurred by the first postbleach image acquisition and the subsequent recovery captured in our analyses mainly reflects the reaction component, such as the dissociation of molecules from their bound immobile state (Figures 2E and 2F). In the majority of cells, a one-reaction rate model gave the best fit (Figure 2G). When the two-reactions model was better, one of the rates was always similar to the dissociation rates generated in the single rate analysis and the other rate was much faster with very wide confidence intervals. Overall the two-reactions approach did not improve the match to the experimental data enough to justify the increase in number of parameters (Table S1). We therefore used a one-reaction model to extract YAP1 dissociation rate. Intriguingly, this rate in the nucleus was significantly lower in CAF1 (0.39 s^−1^) than in NF1 (0.56 s^−1^) (Figure 2H; Video S2). This suggests that YAP1 associates more stably with a nuclear partner in CAFs, likely a chromatin-bound factor. Depletion of endogenous YAP1 did not significantly alter these dissociation rates, excluding the possibility of saturation of the chromatin by endogenous YAP1 (data not shown). Similarly, FRAP analyses in the cytoplasm determined that the observed fluorescence recoveries could be explained by dissociation rates of 0.67 s^−1^ in NF1 and 0.65 s^−1^ in CAF1 (Figure 2H; Video S3). This means that there is relatively little difference between normal and activated fibroblasts and that YAP1 does not have a long-lived site of sequestration in the cytoplasm.

**Figure 3:**
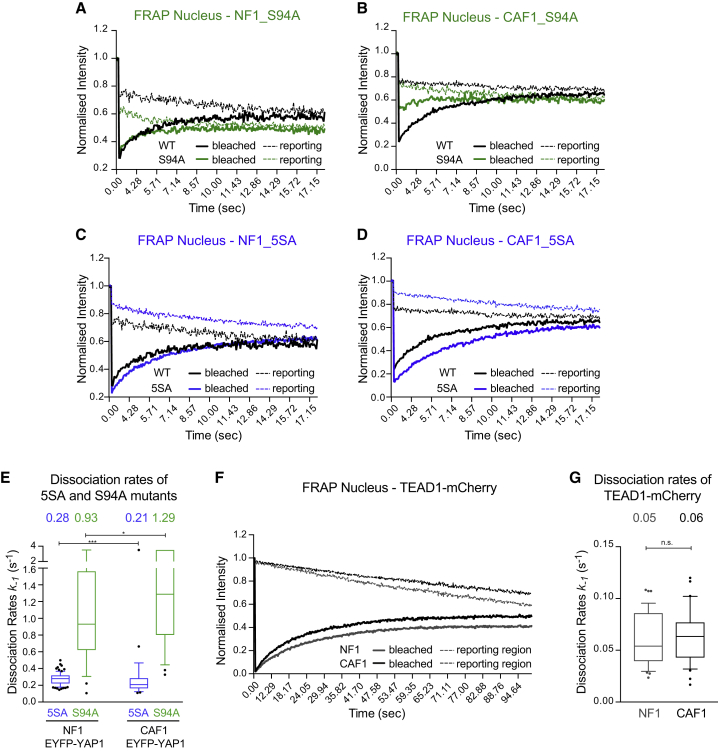
Binding to TEAD Transcription Factors Modulates YAP1 Nuclear Mobility (A) Graph showing the median intensities of EYFP-YAP1_S94A and EYFP-YAP1 (reproduced from Figure 2E for representation) from bleached (plain line) and reporting (dotted line) regions upon nuclear FRAP in NF1. n = 30 cells, three biological replicates. (B) Equivalent graph to (A) for nuclear FRAP in CAF1 with EYFP-YAP1_S94A, n = 30 cells, three biological replicates. (C) Equivalent graph to (A) for nuclear FRAP in NF1 with EYFP-YAP1_5SA, n = 30 cells, three biological replicates. (D) Equivalent graph to (A) for nuclear FRAP in CAF1 with EYFP-YAP1_5SA, n = 25 cells, three biological replicates. (E) Boxplot (10 and 90) showing the dissociation rates of 5SA and S94A mutants in the nucleus of NF1 and CAF1. Values above plots indicate medians. Noisy cells are assumed to have rapidly recovered and are represented with a large arbitrary unit of 3.5 s^−1^. (F) Equivalent graph to (A) for nuclear FRAP of TEAD1-mCherry in NF1 and CAF1, n > 37 for both cell lines, from three biological replicates. (G) Histogram 10–90 percentiles showing the dissociation rates of TEAD1-mCherry in the nucleus of NF1 and CAF1. Values above plots indicate medians. Mann-Whitney U test, n.s., nonsignificant. ^∗^p ≤ 0.05, ^∗∗∗^p ≤ 0.001.

Having established that YAP1 has differential binding in the nucleus in CAF1 compared with NF1, we sought to determine what the binding partner was. The most likely partners of YAP1 in the nucleus are TEAD transcription factors (Zanconato et al., 2015, Zhao et al., 2008). We therefore repeated the FRAP analyses with S94A mutation, which is known to abrogate TEAD interaction. Figures 3A and 3B show that S94A mutation did indeed affect fluorescence recovery (see also Video S4). In some cases, it became so rapid that it was not possible to reliably determine a dissociation rate. In the CAFs in which a rate could be measured, it was 0.97 s^−1^, while the median rate of the whole dataset including the rapid recovery cells was 1.29 s^−1^ (Figure 3E). These data confirm that the altered dynamics of EYFP-YAP1 in the nucleus of CAF1 is due to TEAD binding. In contrast, the active 5SA mutant, unable to bind 14-3-3 proteins, exhibited a slower dissociation rate, consistent with increased TEAD binding and transcriptional activation (Figures 3C–3E; Video S4). To determine if the dissociation rate measured in CAF1 (0.21 s^−1^) represents the dissociation rate of an intact YAP1-TEAD complex from chromatin or the dissociation rate of YAP1 from TEAD that remains bound to chromatin, we performed FRAP of TEAD1-mCherry. Figures 3F and 3G show that TEAD1-mCherry had a slower recovery than YAP1 with a dissociation rate of 0.05/0.06 s^−1^ (see also Video S5). This suggests that in CAFs, TEAD1 typically spends 30 s bound to chromatin, whereas YAP1 typically spends ∼2.5 s bound to TEADs, indicating that the rate we measured primarily represents the dissociation of YAP1 from TEAD transcription factors.

### YAP1 Dynamics Is Regulated by Nuclear Export

**Figure 4:**
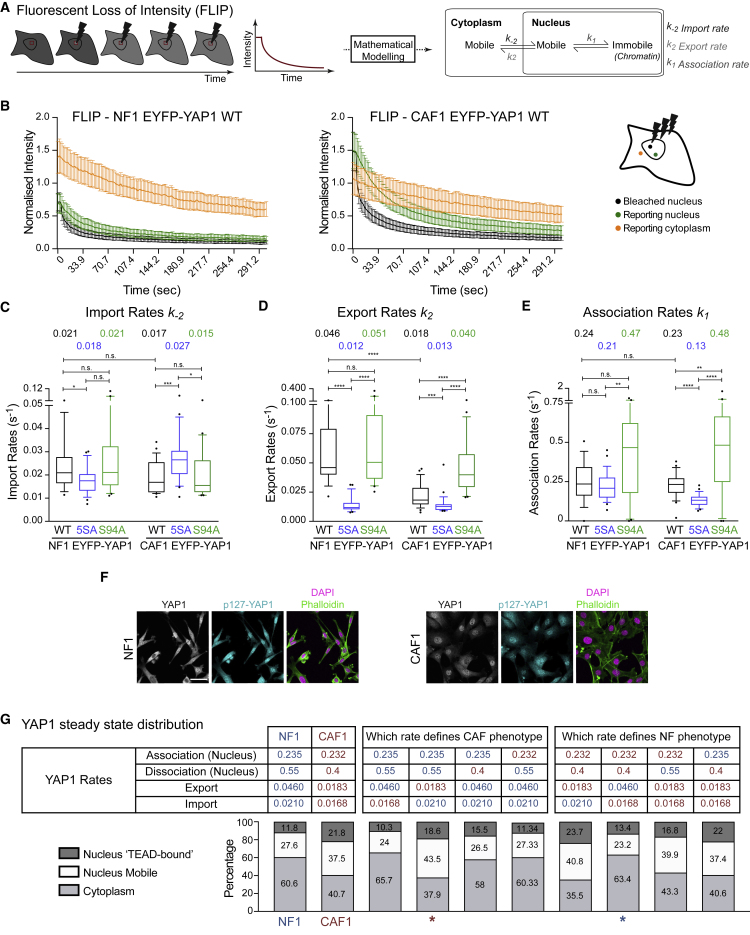
YAP1 Dynamics Is Regulated by Nuclear Export (A) Schematic of the FLIP experiment and parameters extracted with mathematical modeling. (B) Graph showing the intensities of EYFP-YAP1_WT from bleached (black), nuclear reporting (green), and cytoplasmic reporting (orange) regions upon nuclear FLIP in NF1 and CAF1. Graph represents mean with 95% confidence interval (CI). n = 26 cells for each, from three biological replicates. (C–E) Boxplot (10&90) showing the import (*k-*_*2*_), export (*k*_*2*_) and association rates (*k*_*1*_) of EYFP-YAP1_WT, 5SA or S94A in NF1 and CAF1. Values above plots indicate medians. Noisy cells are assumed to have rapidly recovered and are represented with a large arbitrary unit of 3.5 s^−1^. (F) Representative images of S127YAP1 compared with total YAP1 in NF1 and CAF1. Scale bar, 50 μm. (G) Estimation of YAP1 steady-state distribution in normal condition and upon modification of specific rates. Asterisks highlight CAF-like (red) or NF-like (blue) distributions. Mann-Whitney U test, n.s., nonsignificant. ^∗^p ≤ 0.05, ^∗∗^p ≤ 0.01, ^∗∗∗^p ≤ 0.001, ^∗∗∗∗^p ≤ 0.0001. See also Figure S4.

Next, we turned our attention to determining the rate of nuclear import and export. We employed FLIP analysis to continually bleach EYFP-YAP1 in the nucleus and assess import rates (k_−2_), export rates (k_2_), as well as the nuclear association rates (k_1_) (Figure 4A). Figure 4B shows the different loss of signal at the bleached point (black) and “reporting” points in the nucleus (green) and the cytoplasm (orange) that were selected for being a similar distance from the bleached point. FLIP analyses on fast-diffusing EGFP revealed no differences in the loss of signals between the bleached and the reporting point in the nucleus and a fast drop of the cytoplasmic intensity (Figure S4A). The greater difference in curves of the nuclear and cytoplasmic “reporting” points (green and orange) in CAF1 compared with NF1 provide a qualitative indication of reduced exchange between the nucleus and the cytoplasm in these cells (Figure 2B). However, we sought quantitative determination of the rates (Ungricht et al., 2015, Wustner et al., 2012). Closer inspection of the imaging data revealed that EYFP-YAP1 signal did not diminish uniformly across the nucleus (Video S6). This is not unexpected given that YAP1 dynamics in the nucleus result from the combined action of diffusive and reactive processes (i.e., TEAD binding), but this meant it would not be sensible to consider nuclear EYFP-YAP1 signal as a single variable, as shown in Figure 4B. To overcome this, we divided the entire cell into regions of 2.1 μm^2^ and measured EYFP-YAP1 signal in each region and constructed a partial differential equation (PDE)-based model of YAP1 dynamics (Figure S4; Mathematical Methods section of Mathematical Modeling and Model Validation). This accounted for the mobility of YAP1 in the nucleus and in the cytoplasm. The diffusion value (*D*) and dissociation values (*k*_*−1*_) determined by FRAP analysis were then used to model the non-uniform decrease in EYFP-YAP1 signal across the nucleus. The import (*k*_*−2*_) and export (*k*_*2*_) rates were calculated based on EYFP-YAP1 signal intensity in grid-points either side of the nuclear boundary (Figure S4B). This technique allows the model to fit the data according to all spatial locations, as opposed to just the bleached region and two user-selected regions in the nucleus and the cytoplasm, generating greater robustness in the model and certainty in the estimated parameters (Figure S4C). In addition to the import and export rates, this analysis also generated a value for the association rate (*k*_*1*_) of YAP1 with TEAD, that is the reverse process of the dissociation rate measured in Figure 3.

The quantitative analysis described above revealed that YAP1 import was remarkably comparable between NF1 (0.021 s^−1^) and CAF1 (0.017 s^−1^) (Figure 4C). Further, mutation of the serine residues involved in 14-3-3 binding did not greatly affect YAP1 import in NF1 (wild-type [WT] 0.021 s^−1^ and 5SA 0.018 s^−1^) (Figures 4C and S4D–S4G; Video S7). This argues against 14-3-3 mediated sequestration in the cytoplasm preventing nuclear import. Further, the increased levels of nuclear YAP1 in CAF1 cannot be attributed to faster import. Figure 4D shows that the export rate of EYFP-YAP1 was different between NF1 and CAF1. YAP1 export was 2.5 times slower in CAF1, compared with NF1 (NF1 0.046s^−1^, CAF1 0.018s^−1^). The rate of YAP1 export was also diminished by mutation of LATS1/2 target sites in both cells (NF1_5SA 0.012s^−1^, CAF1_5SA 0.013s^−1^). However, the reduced export rate of YAP1 in CAF1 cannot be attributed to reduced LATS1/2-mediated phosphorylation as both pS127-YAP1 and active pLATS1/2 levels are equivalent between NF1 and CAF1 (Figure S4H). Sensitivity analysis revealed that increasing or decreasing the dissociation rate by 50% (which approximates to the range of values measured by FRAP in Figure 2) had minimal effect on the import and export rates (Mathematical Methods section of Mathematical Modeling and Model Validation), increasing our confidence in our results. Together, these data establish the regulation of nuclear export as a key difference between normal and activated fibroblasts. They also suggest that nuclear export can be modulated LATS1/2 phosphorylation dependent and independent ways. The reduced export of EYFP-YAP1_5SA implies that phosphorylated YAP1 is present in the nucleus, which is contrary to the view of its retention in the cytoplasm bound to 14-3-3 proteins. We sought direct evidence of this by performing staining of pS127-YAP1. Figure 4F shows that phosphorylated YAP1 could indeed be found in the nucleus or both NF1 and CAF1, confirming one prior report (Wada et al., 2011).

We additionally generated EYFP-YAP1_WW and EYFP-YAP1_Δ5C to test the role of the WW (Chan et al., 2011, Iglesias-Bexiga et al., 2015, Komuro et al., 2003, Oka et al., 2008, Zhao et al., 2009) and C-terminal (Oka et al., 2010) domains (Figure 1A) that have previously been implicated in YAP1 regulation. The Δ5C mutant exhibited more cytoplasmic localization and lower transcriptional activity in CAFs (Figures S4J–S4L, red), whereas the WW mutant had similar distribution to WT YAP1, but also exhibited reduced transcriptional competence (Figures S4J–S4L, coral). Intriguingly, EYFP-YAP1_Δ5C had slightly elevated export rates and slightly reduced import rates compared with EYFP-YAP1, which would explain its moderately reduced nuclear accumulation (Figure S4M). The WW mutant had increased rates of both import and export, suggesting shorter dwell times in the nucleus (Figure S4M).

Analysis of the FLIP data also enabled the association rate of YAP1 to TEAD to be determined. This parameter, alongside dissociation and diffusion rates, affects the spatial variability of intensity within the nucleus (Mathematical Methods section of Mathematical Modeling and Model Validation). For both NF1 and CAF1, and for the various YAP1 mutants, the association rate was consistently in the range of 0.15–0.5 s^−1^ (Figure 4E). Unsurprisingly, the association rate showed greater sensitivity to changes in the dissociation rate (Mathematical Methods section of Mathematical Modeling and Model Validation). Together these data enable a description of the steady-state distribution and dynamics of YAP1 in both normal and activated fibroblasts (Figure 4G). Briefly, we determine that ∼40% of EYFP-YAP1 is in the nucleus in NF1, with ∼12% in a chromatin-bound fraction. In contrast, ∼60% of EYFP-YAP1 is nuclear in CAF1, with ∼22% bound to chromatin. This ∼50% increase in chromatin binding in CAFs is similar to the two times increase in transcriptional activity (Figure 1D). Steady-state distributions were generated for the 5SA and S94A mutants (Figure S4I). 5SA mutant distributions are similar between NF1 and CAF1, showing an increase of both nuclear fractions (chromatin-bound and mobile). The steady-state distributions of S94A mutant are also similar between both NF1 and CAF1 and resembled the steady-state of YAP1 distribution in NF1 (see also Figure 4G). Individually substituting dynamic parameters between NF1 and CAF1 indicated that simply swapping the export rate and keeping all the other NF1 parameters (red star) was sufficient to yield a CAF-like distribution of YAP1, and vice versa (blue star) (compare columns 1, 2, 4, and 8 in Figure 4G).

**Figure 5:**
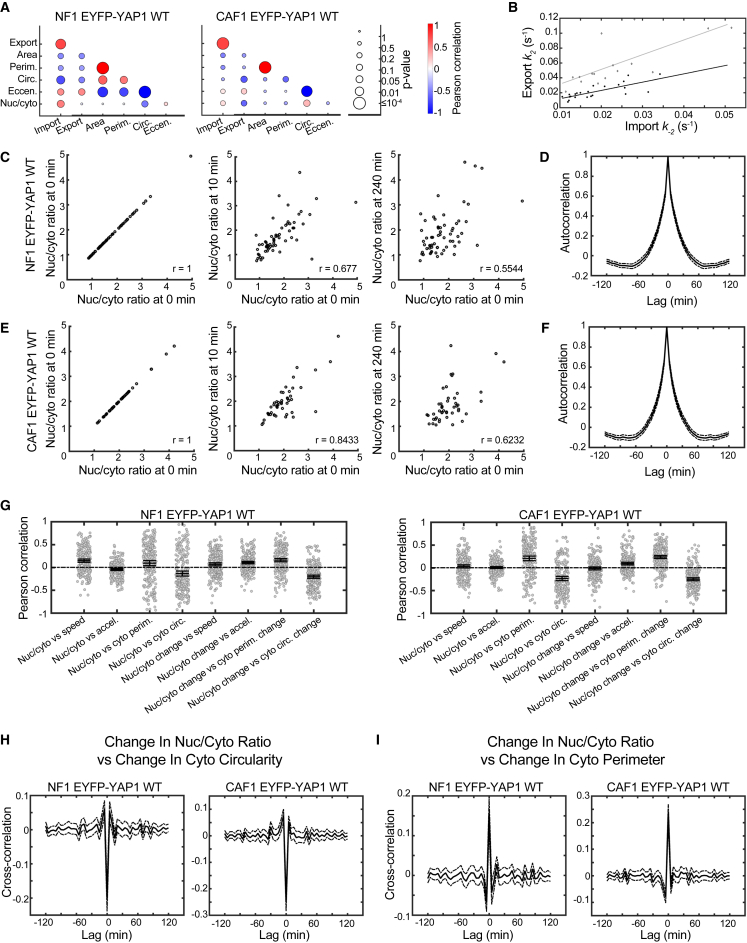
YAP1 Dynamics Correlate with Cellular Morphology (A) Plot illustrating correlation between import and export and nuclear morphology (left NF1 EYFP-YAP1_WT 16 cells, right CAF1 EYFP-YAP1_WT 23 cells). Circle color reflects Pearson correlation (red positive, blue negative) and circle size the p value of the correlation (large, significant; small, nonsignificant). (B) Scatterplot of import versus export and line of the best fit for NF1 EYFP-YAP1_WT (gray) and CAF1 EYFP-YAP1_WT (black). (C–F) Scatterplots of nuclear-to-cytoplasmic ratio at time 0 min versus time 0 min, 10 min and 240 min for NF1 EYFP-YAP1_WT (C) and CAF1 EYFP-YAP1_WT (n > 46 cells) (E) and autocorrelations of nuclear-to-cytoplasmic ratio (mean of all cells, solid line; and 95% CI, dot-dash line) for NF1 EYFP-YAP1_WT (D) and CAF1 EYFP-YAP1_WT (F). A total of 231 cells tracked for NF1 EYFP-YAP1_WT and 221 cells tracked for CAF1 EYFP-YAP1_WT. (G) Scatterplots and 95% CIs for various Pearson correlations of nuclear-to-cytoplasmic ratio, cellular morphology, cell speed, and their derivatives for NF1 EYFP-YAP1_WT and CAF1 EYFP-YAP1_WT. (H and I) Cross-correlations of change in nuclear-to-cytoplasmic ratio and change in cytoplasmic circularity (left) and change in cytoplasmic perimeter (right) for NF1 EYFP-YAP1_WT (H) and CAF1 EYFP-YAP1_WT (I). Mean of all cells, solid line; 95% CI, dot-dash line.

A feature of our analysis is the relatively large variance in both the morphological and rate measurements, which could suggest intercellular variability within the normal fibroblast and CAF populations. To explore this in more detail, we analyzed correlations at the single-cell level. Figure 5A shows correlation matrices for import, export, association, and a range of cell morphology parameters. In addition to the expected correlations of area with perimeter and anti-correlation of circularity with perimeter, we noted a striking correlation between import and export rates (Figure 5B). This argues that the nuclear envelope of some cells is more amenable to YAP1 transit in both directions. Further, we find that nuclear eccentricity is correlated with higher import and export rates, supporting the idea that cells with “deformed” nuclei present less of a barrier to YAP1 transit. Interestingly, the gradient of the link between import or export and eccentricity or circularity is shallower in CAF1 (Figures S5A–S5D). This indicates that NF1 is more sensitive to nuclear deformation than CAF1. Together, these data argue that nuclear deformation increases the rate at which the system can get to equilibrium, especially in NF1, but that the equilibrium position is not dictated by nuclear deformation.

### Cell Shape Changes Trigger Rapid Re-distribution of YAP1

Import and export rates in the order of 0.01–0.05 s^−1^ would permit pronounced shifts in the nuclear-to-cytoplasmic balance of YAP1 within minutes. To investigate how rapidly the nuclear-to-cytoplasmic ratio (N/C ratio) of EYFP-YAP1 changed in the absence of exogenous perturbation, we analyzed 5-hr time-lapse movies (Videos S8 and S9). The N/C ratio of individual cells was tracked over time together with positional and morphological information. Ten minutes after beginning tracking, the N/C ratio was well correlated with the initial value; however, this correlation was reduced at 4 hr (Figures 5C and 5E). Autocorrelation analysis of EYFP-YAP1 N/C ratio revealed significant correlation over a period of up to 30 min (Figures 5D and 5F). The greater decay in autocorrelation compared with correlations of Figures 5C and 5E reflects intercellular variability being larger than the dynamic variability within single cells. As expected, both NF1 and CAF1 exhibit significant cell shape changes and migratory behavior over the 5-hr imaging period (Videos S8 and S9). We therefore sought to correlate changing N/C ratio with cell shape changes, cell speed, or acceleration. This revealed a striking positive correlation between increasing N/C ratio (i.e., a positive derivative of N/C ratio) and increasing cytoplasmic perimeter and a negative correlation between N/C ratio and cytoplasmic circularity (Figures 5G and S5E). A weak correlation was observed with cytoplasmic acceleration. Cross-correlation analysis demonstrated that the change in N/C ratio occurred within 5 min of the change in cell shape, which is the limit of resolution of the long time-frame videos (Figures 5H, 5I, and S5F–S5H). Together, these data show that during the normal migratory behavior of fibroblasts, cell stretching (increase in perimeter and reduction in circularity) and acceleration is coupled to increasing nuclear YAP1.

### Actin and Src-Family Kinases Regulate YAP1 Export

**Figure 6:**
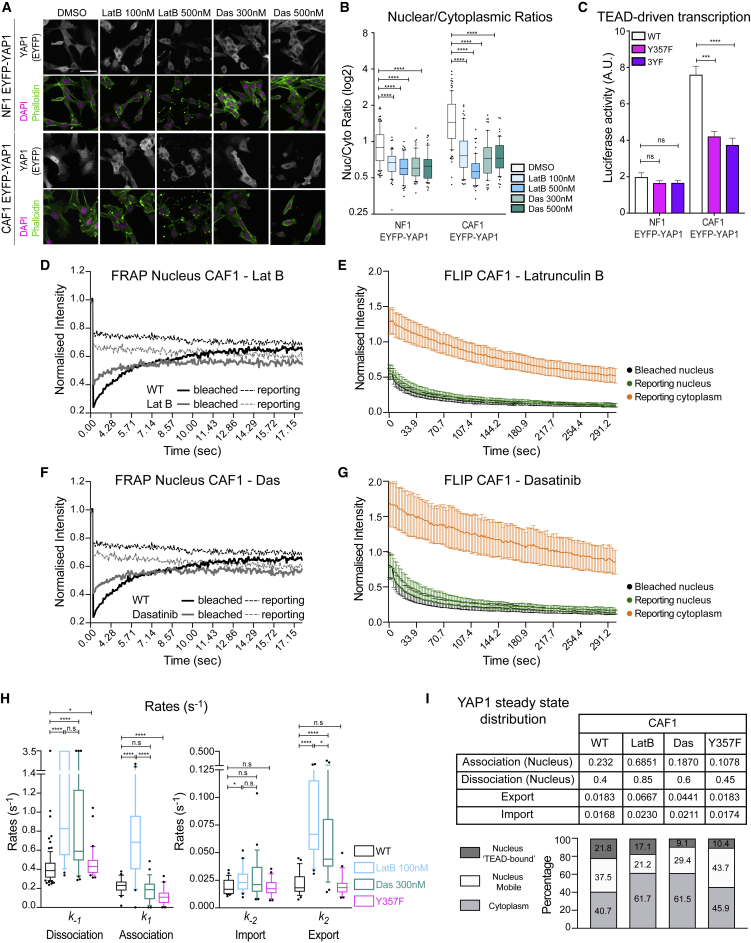
Actin and Src-Family Kinases Regulate YAP1 Export (A) Representative images of EYFP-YAP1 localization in NF1 and CAF1 treated with DMSO or 100 nM/500 nM latrunculin B and 300 nM/500 nM dasatinib. Scale bar, 50 μm. (B) Boxplot (10 and 90) of nuclear-to-cytoplasmic ratio (log2 scale) corresponding to (A). n > 60 cells, at least two independent experiments. (C) Luciferase assay of EYFP-YAP1, EYFP-YAP1_Y357F, and EYFP-YAP1_3YF in NF1 and CAF1. Bars represent mean ± SEM of at least three independent experiments, each with three technical replicates. (D) Graph showing the median intensities of EYFP-YAP1 from bleached (plain line) and reporting (dotted line) regions upon nuclear FRAP in CAF1 treated with 100 nM latrunculin B, n = 29 cells, three biological replicates. EYFP-YAP1_WT with no treatment is reproduced from Figure 2E for representation. (E) Graph showing the intensities of EYFP-YAP1 from bleached (black), nuclear reporting (green), and cytoplasmic reporting (orange) regions upon nuclear FLIP in CAF1 treated with 100 nM latrunculin B. Graph represents mean with 95% CI. n = 30 cells, three biological replicates. (F) Equivalent graph to (D) upon nuclear FRAP in CAF1 treated with 300 nM dasatinib, n = 30 cells, three biological replicates. EYFP-YAP1_WT with no treatment is reproduced from Figure 2E for representation. (G) Equivalent to (E) upon FLIP in CAF1 treated with 300 nM dasatinib, n = 30 cells, three biological replicates. (H) Boxplot (10 and 90) showing the different rates. Rates of EYFP-YAP1_WT are reproduced from Figure 2H for representation. Noisy FRAP cells are assumed to have rapidly recovered and are represented with a large a.u. of 3.5 s^−1^. Medians are indicated in the table (I). (I) Estimation of YAP1 steady-state distribution. EYFP-YAP1_WT in CAF1 is reproduced from Figure 4G for representation. Mann-Whitney U test, n.s., nonsignificant. ^∗^p ≤ 0.05, ^∗∗∗^p ≤ 0.001, ^∗∗∗∗^p ≤ 0.0001. See also Figure S5.

The data above reinforce the tight linkage between cytoskeletal state, which determines cell shape changes during migration, and YAP1 localization. We therefore focused on understanding how the actomyosin cytoskeleton modulates YAP1 dynamics and shuttling. The fluorescent properties of blebbistatin precluded its use in photobleaching experiments (Kolega, 2004), we therefore used latrunculin B to disrupt the actin cytoskeleton. We additionally used dasatinib, an Abl and Src-family kinase inhibitor, as previous analyses had implicated these kinases in “mechano”-regulation of YAP1 (Calvo et al., 2013, Elbediwy et al., 2016, Li et al., 2016, Rosenbluh et al., 2012). We confirmed that both inhibitors promote endogenous YAP1 and EYFP-YAP1 cytoplasmic accumulation and reduce YAP1-dependent transcription (Figures 6A, 6B, and S6A–S6C; controls of S6C are reproduced from Figure 1E). Similar effects on YAP1 localization were obtained using saracatinib, which is a more specific Src-family kinase inhibitor, but not with imatinib, which is an Abl/BCR-Abl inhibitor (Figures S6D and S6E).

Tyrosine phosphorylation on Y341/Y357/Y394 has been implicated in YAP1 regulation by Src-family kinases, and dasatinib reduced Y357 phosphorylation (Calvo et al., 2013, Levy et al., 2008, Li et al., 2016, Rosenbluh et al., 2012, Tamm et al., 2011, Zaidi et al., 2004). We also observed a reduction in YAP1 Y357 phosphorylation in CAF1 treated with latrunculin B or blebbistatin (Figure S6F), thereby formally demonstrating the linkage of actomyosin to YAP1 tyrosine phosphorylation. We next investigated whether mutation of Y357 or Y341/Y357/Y394 (3YF) together would phenocopy the effects of disrupting actin or Src-family kinases. These mutations reduced the transcriptional competence of YAP1 (Figure 6C), and mutation of Y357F was sufficient to decrease the contractile phenotype of CAF1 (Figures S6G and S6H). However, Y357F and Y3F mutants exhibited surprisingly similar subcellular distribution as EYFP-YAP1 (Figures S6I and S6J). Further, FRAP and FLIP analyses confirm that mutation of Y357F had little effect on the rates of import, or export (Figures 6H, S6K, and S6L; Videos S10 and S11), although the association rate was reduced leading to a lower fraction of EYFP-YAP1_Y357F bound to chromatin (∼10%) compared with EYFP-YAP1 (∼22%) (Figure 6I). These data demonstrate that the effect of latrunculin and dasatinib on YAP1 localization cannot be accounted for solely by direct YAP1 tyrosine phosphorylation.

To gain insight into how actin and Src-family kinases regulate YAP1 localization we returned to FRAP and FLIP analysis. This revealed that both latrunculin B and dasatinib increased the dissociation rate of YAP1 from chromatin (Figures 6D–6H; Video S10). More crucially, YAP1 export rates increased significantly following latrunculin B and dasatinib treatment (Figure 6H; Video S11), returning to rates similar to those in NF1 (Figure 3H). Furthermore, EYFP-YAP1 CAF1 treated with latrunculin B or dasatinib present similar protein distribution that in NF1 (Figure 6I compared with Figure 4G). S127 phosphorylation of YAP1 was not affected by latrunculin B or dasatinib treatment (Figure S6M). However, the non-phosphorylated EYFP-YAP1_5SA mutant remained nuclear even in latrunculin B- and dasatinib-treated cells (Figures S6N and S6O). These data are consistent with elevated actin and Src-family kinase activity reducing the rate of export of only phosphorylated YAP1. This was reinforced by the minimal boost of latrunculin B and dasatinib to the export rate of EYFP-YAP_5SA (Figure S6P). Together, these data reveal that perturbation of actin and Src-family kinases alters YAP1 regulation within the nucleus; specifically, Y357-dependent transcriptional activation, dissociation from chromatin, and export.

### Import/Export Machinery Screen Identifies XPO1 as Key Mediator of YAP1 Export

**Figure 7:**
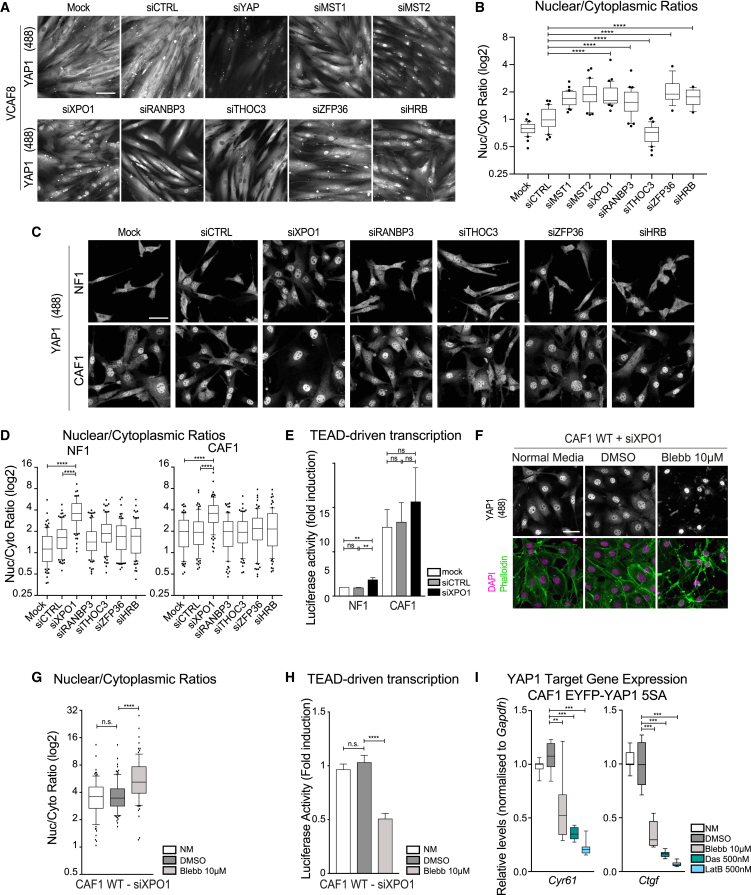
XPO1 Mediates YAP1 Export, but Nuclear YAP1 Remains Actomyosin Regulated (A) Representative images of the secondary screen showing endogenous YAP1 staining in human VCAF8 upon siRNA knockdown of control targets and several hits. Scale bar, 100 μm. (B) Boxplot (10&90) of nuclear-to-cytoplasmic ratio (log2 scale) corresponding to one experimental repeat of the secondary screen. n > 15 cells. (C) Representative images showing the effect of specified hits on endogenous YAP1 localization in murine cells, NF1 and CAF1. Scale bar, 50 μm. (D) Boxplot (10 and 90) of nuclear-to-cytoplasmic ratio (log2 scale) corresponding to three experimental repeats in NF1 and CAF1. n > 89 cells. (E) Luciferase assay of NF1 and CAF1 upon knockdown of specified siRNA. Bars represent mean ± SEM of five independent experiments, each with three technical replicates. (F) Representative images of endogenous YAP1 localization upon XPO1 depletion followed by 10 μM blebbistatin treatment. Scale bar, 50 μm. (G) Boxplot (10 and 90) of nuclear-to-cytoplasmic ratio (log2 scale) corresponding to (F). n > 80 cells from three experimental repeats. (H) Luciferase activity of CAF1 upon XPO1 depletion in normal media (NM) and treated with DMSO or 10 μM blebbistatin. Bars represent mean ± SEM of five independent experiments, each with three technical replicates. (I) Boxplot (minimum to maximum) of qRT-PCR of two YAP1 target genes normalized to GAPDH in CAF1 EYFP-YAP1_5SA cell line in NM, or treated with DMSO, 10 μM blebbistatin, 500 nM dasatinib, or 500 nM latrunculin B. Data summary of four independent experiments, each with two technical replicates. Mann-Whitney U test, n.s., nonsignificant. ^∗∗^p ≤ 0.01, ^∗∗∗^p ≤ 0.001, ^∗∗∗∗^p ≤ 0.0001.

The results above indicate that nuclear export is a key step in the regulation of YAP1. To learn more about the regulation of YAP1 entry and exit from the nucleus, we performed a small interfering RNA (siRNA) screen targeting the known complement of nuclear import and export machinery (Figure S7A). The siRNA library was targeted against human genes; we therefore carried out the screen in two human cancer-associated fibroblasts. To identify regulators of both import and export, we sought to identify conditions in which the levels of YAP1 in the nucleus and cytoplasm were roughly equivalent. Figure S7B shows that increasing the confluence especially for VCAF8 led to similar YAP1 staining intensity in both nucleus and cytoplasm. Figures S7C and S7D confirm that nuclear YAP1 localization in human VCAF4 and VCAF8 depends upon actomyosin function. VCAF4&8 were reverse transfected with human siRNA pools targeting 143 different genes. After 4 days, we fixed and stained the cells for YAP1 and images were acquired using a Cellomics Arrayscan (Figure S7A). siRNA targeting YAP1 and MST1/MST2 had the expected effect of reducing overall YAP1 levels and increasing nuclear YAP1, respectively (Figures 7A and 7B). The images were then assessed in a double-blinded manner for either altered nuclear or cytoplasmic distribution of YAP1. Several siRNAs consistently perturbed YAP1 localization (summarized in Table S2); 14 genes were selected for a secondary screen using three different siRNAs distinct from those used in the original screen. XPO1, RANBP3, ZPF36, and HRB were found to consistently, and with multiple siRNAs, increase the nuclear localization of YAP1 (Figures 7A and 7B). In contrast, THOC3 decreased the nuclear localization of YAP1. A tertiary screen in the murine NF1 and CAF1 fibroblasts highlighted the central role of XPO1 in YAP1 nuclear export (Figure 7C). Quantification revealed an increased YAP1 nuclear localization with approximately four times (NF1) and two times (CAF1) more YAP1 in the nucleus compared with the cytoplasm (Figure 7D). The level of XPO1 knockdown was confirmed in NF1 and CAF1 (Figure S7E). An off-target effect due to the use of a siRNA pool was excluded by checking YAP1 localization using single oligos (Figure S7F). XPO1 depletion did not affect β-catenin or c-jun localization (Figure S7G). We next explored if XPO1 depletion would affect TEAD-driven transcription. Figure 7E shows that depletion of XPO1 leads to a small, but significant, increase in TEAD-dependent transcription in NF1, similar increases were observed in endogenous YAP1 target genes in both NF1 and CAF1 (Figure S7H). These data demonstrate that XPO1 is a key mediator of YAP1 export in both human and murine cells. Further, nuclear accumulation of YAP1 in NFs is sufficient to modestly increase TEAD-dependent transcription, albeit not as dramatically as mutation of LATS1/2 phosphorylation sites (Figure 1H).

### Nuclear YAP1 Is Regulated by Actomyosin

We next determined the relationship between XPO1-mediated export and cytoskeletal regulation of YAP1. Blebbistatin treatment did not promote the cytoplasmic translocation of YAP1 following XPO1 depletion (Figures 7F and 7G), arguing that cytoskeletal regulation is reducing XPO1-dependent YAP1 nuclear export. Finally, we asked whether inhibiting export prevented cytoskeletal regulation of YAP1 transcriptional function. Analysis of endogenous YAP1 target genes and TEAD-dependent reporter assays indicated that latrunculin B, blebbistatin, and dasatinib still reduced the transcription competence of YAP1, even though it remained in the nucleus (Figures 7H and S7I). As shown above, actomyosin blockade and dasatinib treatment reduce YAP1 Y357 phosphorylation but do not affect LATS1/2-mediated S127 phosphorylation (Figures S6F and S6M). These data predict that cytoskeletal and tyrosine kinase perturbation should reduce the transcriptional competence of EYFP-YAP1_5SA despite not altering its localization (shown in Figure S6O). Figures 7I and S7J confirm that latrunculin B, blebbistatin, dasatinib, and saracatinib all reduce the ability of EYFP-YAP1_5SA to drive the expression of target genes. These data show that two divergent mechanisms exist by which the cytoskeleton and Src-family kinases influence YAP1 activity. First, they reduce the export of LATS1/2-phosphorylated YAP1, thereby controlling its subcellular localization. Second, cytoskeletal integrity influences the tyrosine phosphorylation of YAP1 required for its maximal transcriptional competence.

## Discussion

The transcriptional regulator YAP1 is critically important for the control of growth of epithelial tissues and organ size (Piccolo et al., 2014, Zhao et al., 2007, Zhao et al., 2011). YAP1 also plays a key role in fibroblast activation in pathological contexts (Calvo et al., 2013). The regulation of YAP1 can be divided into a “canonical” pathway involving negative LATS1/2-mediated serine phosphorylation events and a more recently described pathway linked to cytoskeletal integrity and Src-family kinase function (Low et al., 2014). However, the interplay between these pathways and how they regulate the subcellular dynamics of YAP1 is not understood. We have used photobleaching of fluorescently tagged YAP1 combined with molecular perturbations and mathematical modeling to tackle this issue. The use of ordinary and partial differential equation-based methods have many benefits over the traditional “t_1/2_” (half-time) and immobile fraction analysis historically used for FRAP analysis. t_1/2_ metrics overlook the possibility of multiple processes occurring simultaneously and the estimation of the immobile fraction relies on judging asymptotic points with noisy data in systems that may not even exhibit such behavior. Our FRAP analysis enables reaction rates to be determined. Furthermore, the implementation of PDE analysis of FLIP data avoids the subjective selection of reporting points for analysis and provides parameter estimates that robustly describe spatial variability.

Our quantitative analysis reveals several notable findings. First, YAP1 is highly dynamic with molecules shuttling in and out of the nucleus in a timescale of 50 to 100 s. Second, the interaction with TEAD is a very short-lived with dissociation rates ∼0.5 s^−1^ for WT YAP1 and only ∼0.2s^−1^ for the strong gain-of-function 5SA mutant. This contrasts with the DNA binding of TEAD, which has a dissociation rate two orders of magnitude slower. These data indicate that a time frame of seconds is required for YAP1 to trigger the molecular events that promote RNA polymerase II-dependent transcription, possibly the engagement of TEAD-occupied enhancers with the more proximal core machinery of promoters (Galli et al., 2015, Zanconato et al., 2015). The third observation is the dominance of nuclear export as a point of YAP1 regulation. Serine phosphorylation is required for nuclear export as EYFP-YAP1_5SA has a greatly reduced export rate and remains nuclear even when the cytoskeleton or Src-family kinases are perturbed. This realization represents a shift in the view that LATS1/2-phosphorylated YAP1 is stably sequestered in the cytoplasm. Instead, the increased cytoplasmic localization of LATS1/2-phosphorylated YAP1 is the result of increased nuclear export. In agreement with this view, pS127 YAP1 is observed in the nucleus (Figure 4F). Furthermore, constraining YAP1 in the nucleus as a result of XPO1 depletion does not alter pS127 phosphorylation, which suggests that the phosphatase that counteracts LATS1/2 phosphorylation is located in the cytoplasm.

Serine phosphorylation is not the only factor influencing YAP1 localization. Targeting actin or Src-family kinase function in CAFs increases the export rate to similar levels to those in NFs without changing LATS1/2-mediated YAP1 phosphorylation. Thus, instead of cytoskeletal integrity releasing YAP1 from a cytoplasmic anchor, we propose that nuclear import is constitutive. This step is not regulated by the cytoskeleton, instead, the cytoskeleton modulates XPO1-mediated nuclear export. The exact mechanism by which this is achieved remains to be investigated; we can exclude Y341/357/394 phosphorylation. It is possible that emerin phosphorylation is involved. This protein links the inner nuclear membrane to the nuclear lamina and is subject to Src-mediated phosphorylation following nuclear stress (Tifft et al., 2009). Further, it has been implicated in regulating the subcellular distribution of two other transcriptional regulators, β-catenin and MKL1/MRTF (Guilluy et al., 2014, Markiewicz et al., 2006).

The strong correlation of YAP1 import and export rates in cells was unexpected, but fits with the proposal that deformation of nuclear pores alters the energetic penalty for protein transit into the nucleus (Elosegui-Artola et al., 2017). In support of this, import and export rates were correlated with nuclear deformation in NF1. Comprehensive functional analysis of the known nuclear import and export machinery identified XPO1 as key for the export of YAP1. This is consistent with previous work using leptomycin B, which can inhibit XPO1 (Nishi et al., 1994). Interestingly, we found no functional conservation in murine cells of numerous other hits in the human system (even though they were validated with multiple siRNAs). This may reflect a multitude of minor secondary export mechanisms that are variable between different fibroblast lineages or species. However, control of subcellular distribution is not sufficient to strongly activate YAP1. Tyrosine phosphorylation of Y357 represents an independent mechanism of YAP1 regulation. It does not affect subcellular localization, but reduces the transcriptional competence of YAP1. The ability of the cytoskeleton to influence YAP1 transcriptional activity even when export is blocked suggests that phosphorylation may occur in the nucleus. There are precedents for nuclear Src activity, including the aforementioned phosphorylation of emerin and regulation of estrogen receptor (Castoria et al., 2012). Taken together, these analyses lead to a re-evaluation of YAP1 regulation (Graphical Abstract). We find no evidence that it is stably sequestered in the cytoplasm. Instead, it cycles frequently in and out of the nucleus and is subject to extensive control of its rate of export. This requires “canonical” LATS1/2 phosphorylation, but the rate is negatively tuned by actomyosin and Src-family kinase activity.

## STAR★Methods

### Key Resources Table

**Table:** 

REAGENT or RESOURCE	SOURCE	IDENTIFIER
**Antibodies**		

Mouse monoclonal Igl2a anti-YAP/TAZ (63.7)	Santa Cruz Biotechnology	Cat# sc-101199; RRID: AB_1131430
Rabbit polyclonal anti-S127P-YAP	Cell Signalling Technology	Cat# 4911S; RRID: AB_2218913
Rabbit polyclonal anti-Y357P-YAP	Abcam	Cat# ab62751; RRID: AB_956486
Rabbit polyclonal anti-GFP tag	Thermo Fisher Scientific	Cat# A11122; RRID: AB_221569
Mouse monoclonal IgG1 anti-beta-tubulin	Sigma	Cat# T7816; RRID: AB_261770
Mouse monoclonal IgG2b anti-pan-14-3-3 (H-8)	Santa Cruz Biotechnology	Cat# sc-1657; RRID: AB_626618
Mouse monoclonal IgG1 anti-beta-catenin (E5)	Santa Cruz Biotechnology	Cat# sc-7963; RRID: AB_626807
Mouse monoclonal IgG1 anti-c-Jun (G-4)	Santa Cruz Biotechnology	Cat# sc-74543; RRID: AB_1121646
Rabbit polyclonal anti-pLATS1 (S909)	Cell Signalling Technology	Cat# 9157; RRID: AB_2133515)
Rabbit polyclonal anti-LATS1	Cell Signalling Technology	Cat# 9153S; RRID: AB_2296754
Mouse monoclonal IgG1 anti-TEAD1 (TEF-1)	Santa Cruz Biotechnology	Cat# sc-376113; RRID: AB_10988229
Rabbit polyclonal anti-TEAD4 (TEF-3)	Santa Cruz Biotechnology	Cat# sc-134071; RRID: AB_10611591
Mouse monoclonal IgG1 anti-XPO1 (CRM1)	BD Biosciences	Cat# 611832; RRID: AB_399312

**Chemicals, Peptides, and Recombinant Proteins**		

Collagen I, High Concentration, Rat Tail	Cornig	354249
Matrigel Matrix	Cornig	356235
Blebbistatin	Calbiochem/Merck	203391
Dasatinib	LC Laboratories	D-3307
LatrunculinB	Enzo Life Sciences	BML T110-0001
Saracatinib	Cambridge Bioscience	11497
Imatinib	LC Laboratories	I-5508
Recombinant GFP	Gift from Grosse Lab, Marburg	N/A
cOmplete EDTA-free Protease inhibitor cocktails	Roche	**04693159001**

**Critical Commercial Assays**		

Dual-Luciferase Reporter Assay System 10-Pack	Promega	E1960
Dynabeads Protein G for Immunoprecipitation	ThermoFisher Scientific	10003D

**Experimental Models: Cell Lines**		

Mouse: NF1 normal mammary gland fibroblast cell line	(Calvo et al., 2013)	N/A
Mouse: CAF1 mammary carcinoma fibroblast cell line	(Calvo et al., 2013)	N/A
Human: VCAF4 vulval cancer associated fibroblast cell line	E.S. and S. Derzsi, unpublished data	N/A
Human: VCAF8 vulval cancer associated fibroblast cell line	R.P.J., E.S., and D. Park, unpublished data	N/A

**Oligonucleotides**		

Primer: qRT-PCR Cyr61 gene mouse forward: GCCGTGGGCTGCATTCCTCT	(Calvo et al., 2013)	N/A
Primer: qRT-PCR Cyr61 gene mouse reverse: GCGGTTCGGTGCCAAAGACAGG	(Calvo et al., 2013)	N/A
Primer: qRT-PCR Ctgf gene mouse forward: CAGCTGCCAGTTTTCCACTACA	(Foster et al., 2017)	N/A
Primer: qRT-PCR Ctgf gene mouse reverse: GGCCTCATTTGGAGTGTCTTG	(Foster et al., 2017)	N/A
Primer: qRT-PCR Gapdh gene mouse forward: TCTTGTGCAGTGCCAGCCT	(Foster et al., 2017)	N/A
Primer: qRT-PCR Gapdh gene mouse reverse: CAATATGGCCAAATCCGTTCA	(Foster et al., 2017)	N/A
siRNA targeting sequences: see Table S3	Qiagen, Dharmacon, Sigma	N/A

**Recombinant DNA**		

Lenti-EF-EYFP-YAP1	This paper	N/A
Lenti-EF-mCherry-TEAD1	This paper	N/A
pPB-puro-H2B-mTurquoise2	This paper	N/A

**Software and Algorithms**		

MetaMorph	Molecular Devices	www.moleculardevices.com
MATLAB Version R2015b	MathWorks	www.mathworks.com
MATLAB Image Processing Toolbox	MathWorks	www.mathworks.com
MATLAB Statistics and Machine Learning Toolbox	MathWorks	www.mathworks.com
Custom MATLAB Algorithms	https://github.com/RobertPJenkins/FLIP-MATLAB-Skeleton-Scripts	N/A

### Contact for Reagent and Resource Sharing

Erik Sahai is the Lead Contact; please send enquiries to erik.sahai@crick.ac.uk. The distribution of patient-derived fibroblasts will require an M.T.A.; this is a requirement of the ethical consent under which the patient cells were obtained.

### Experimental Model and Subject Details

#### Cell Lines

Normal mammary glands fibroblasts (NF1) and mammary carcinoma fibroblasts (CAF1) are described in (Calvo et al., 2013). Briefly, fibroblasts were isolated from female transgenic FVB/n MMTV-PyMT mice and immortalised with HPV-E6 retrovirus. Human vulval (VCAF) fibroblasts were isolated from patient tissue samples collected from patients at Bart’s and the London Hospital under ethical approval 10/H0304/14 and immortalised by pBABE-Hygro-HTERT retroviral transfection. STR profiling was performed on VCAF to confirm that they are unique with no significant similarity to any previously reported cell lines.

### Method Details

#### Experimental Design

All data is derived from multiple experiments, the details of replicates are clarified in the statistics section and the number of replicates indicated in the figure legends. The initial import/export screen analysis was performed blinded and all FRAP and FLIP data was analyzed mathematically, thus blinding is not relevant. No mice or patients were used so randomization or stratification is not pertinent. We explicitly state the cases where data was too noisy to be analyzed and if it was therefore excluded.

#### Plasmids

Both Lenti-EF-EYFP-YAP1 and Lenti-EF-mCherry-TEAD1 were generated using LentiLox 3.7 vector (pLL3.7 – gift from Way lab) where CMV promoter was replaced by EF1 alpha promoter. EYFP-YAP1 fusion was cloned from pDEST EYFP-YAP1 plasmid, gift from Nic Tapon, Francis Crick Institute. The EYFP fluorophore was well-suited to our photobleaching experiments because it is not as photostable as some more ‘optimized’ derivatives of GFP, but is sufficiently bright to be easily imaged (Shaner et al., 2005). YAP1 corresponds to the human YAP1-2γ isoform, which is 504 amino acids long (Gaffney et al., 2012, Sudol, 2013). The different mutants were generated by site-directed mutagenesis. The five serines mutated for the 5SA mutant correspond to serines 61, 109, 127, 164 and 397 (=381) for the isoform used in this study. The three tyrosines known as ‘341’, ‘357’ and ‘394‘ correspond to residues 391, 407 and 444 for the isoform1-2γ used in this study. Δ5C mutant lacks the last five residues (FLTWL) in the PDZ binding domain at the N-terminus of the protein. For the WW domains mutant: the WQDP sequence (residues 199-202) in the first WW domain was changed to AQDA, whereas the WLDP sequence (residues 258-261) of the second WW domain was changed to ALDA. mCherry-TEAD1 was generated fusing TEAD1 from pRK5-Myc-TEAD1 plasmid (Addgene, ♯33109) to mCherry. EGFP and H2B-EGFP constructs were generated previously in the lab and are both cloned under CMV promoter. The two plasmids used for the luciferase experiments, pGL3-49 and pGL3-5xMCAT-49, a gift from Nic Tapon. The pPB-puro-H2B-mTurquoise2 was generated in the lab (details available on request).

#### Transfections, Inhibitors and siRNA

All EYFP-YAP1 constructs (described in Plasmids section) were introduced into the cells using the Lentivirus system. For H2B-turquoise cells the construct was introduced with PiggyBac system. Upon transfection, populations expressing EYFP signal were selected by fluorescence-activated cell sorting (FACS). All cells were cultured in DMEM (Invitrogen), 10% FCS (PAA Labs), 1% ITS (insulin–transferrin–selenium; #41400-045; Invitrogen). Primary and secondary screens were done on human fibroblasts using reverse siRNA transfection with Lipofectamine RNAiMAX (#13778030, Thermo Fisher), where VCAF4 and VCAF8 cells were added directly to wells containing siRNA (384/96well plate for primary/secondary screen respectively) using manufacturer’s instructions. For primary screen, 1000 cells were seeded per well, while for secondary screen 3500-4000 cells were seeded. Cells were fixed 4 days after transfection and stained with YAP/TAZ antibody and DAPI. For siRNA transfection in mouse fibroblasts (NF1 and CAF1), reverse transfection method was used for siRNA transfection with Lipofectamine RNAiMAX (50pmol siRNA per well, 6 well plate) using 60000-75000 cells per transfection. For DNA transfection, cells were subject to transfection with Lipofectamine LTX and Plus reagent (# 15338100, Thermo Fisher) the following day (2.5μg DNA per well, 6 well plate). When specified, drug treatment was started 6hrs after DNA transfection, lasting 48hrs. Otherwise drugs treatment was 4hrs (qRT-PCR and IF analysis). siRNA was purchased from Dharmacon or Sigma, and sequences are listed in Table S2). The following drugs were used: Blebbistatin – 10μm (#203391; Calbiochem /Merck), Dasatinib – 300nM or 500nM (LC Labs), LatrunculinB –100nM or 500nM (LC Labs), Saracatinib 1μm (#11497, Cambridge Bioscience), Imatinib 5μm (LC Labs).

#### Immunofluorescence

All immunofluorescence experiments were performed on cells seeded on glass in a 35mm glass bottom MatTek dish (P35-1.5-14-C, MatTek Co., Ashland, MA, USA). Cells were fixed in 4% paraformaldehyde, washed with PBS and permeabilised by incubation in 0.2 % Triton X100, PBS for 3-5 minutes at room temperature. Samples were subsequently blocked for 1 hour with 5%BSA, PBS before incubation with primary antibody for YAP1 (Santa Cruz, sc101199, 1:200) in 3%BSA, PBS overnight at 4°C. Primary antibody was washed off in 3 washes of 15 minutes with 0.05 % Tween-20, PBS. Fluorescent secondary antibody (Life Technologies) was diluted 1:500 in 3%BSA, PBS and incubated with the samples for one hour, then washed off with 3 washes of 0.05 % Tween-20, PBS. Samples requiring staining for F-actin or the nucleus were also stained with 633-phalloidin (SIGMA) and DAPI (SIGMA) at 1:500 dilution. After 3 washes of 15 min in PBS, secondary antibody in blocking solution was added.

#### Photobleaching Experiments

Cells were plated at low confluence and cultured overnight in 35 mm glass bottom MatTek dishes (P35-1.5-14-C, MatTek Co., Ashland, MA, USA) in DMEM media with 10 % FCS and 1% ITS. One hour prior to imaging, the media was changed to Leibovitz L-15 media (CO2 independent - Invitrogen) -1% serum. The cells were subsequently bleached and imaged with a Zeiss LSM880 inverted confocal laser scanning microscope equipped with an argon laser (Zeiss, Germany) using 514nm laser and a 63X objective (Zeiss, alpha-Plan Apochromat 63x/1.46 NA oil korr TIRF).

For FRAP (Fluorescent Recovery After Photobleaching) experiments, three sizes of circular ROIs were used: small, medium and large. Small, medium and large corresponds to a circle of diameter 11 pixels (∼3.1 μm^2^), 14 pixels (∼4.6 μm^2^) and 17 pixels (∼6.9 μm^2^) in the nucleus, respectively and 14 pixels (∼4.6 μm^2^), 17 pixels (∼6.9 μm^2^) and 20 pixels (∼9.4 μm^2^) in the cytoplasm, respectively. All images were 8-bit and 128x128 pixels. Before photobleaching, 3 measurements of fluorescence were taken. The ROI was then photobleached for 2.9 seconds using maximum laser power. A series of images were then taken every 60 milliseconds for up to 18 seconds, enough to observe complete recovery for most conditions. In order to have complete recovery of intensity EYFP-YAP1 5SA samples were imaged up to 48 seconds. FRAP of TEAD1-mCherry were imaged for 100 seconds. Graphs for FRAP represent only the 17x17 pixels size, except for diffusion analyses where all three sizes are represented.

For FLIP (Fluorescent Loss In Photobleaching) experiments, a single square ROI of 8x8 pixels (4.4 μm^2^) was used. All images were 12-bit and 128x128 pixels. Before photobleaching, 3 measurements of fluorescence were taken. The ROI was then photobleached between every frame for 2 seconds using maximum laser power. A series of 150 images were taken every 2 seconds for up to 5 minutes.

#### Image Analysis

For quantification of subcellular localization of EYFP-YAP1, the nuclear-to-cytoplasmic ratio was calculated. Using MetaMorph (Molecular Devices), the integrated intensities in three square regions of interest (ROI) of the same size, 8x8 pixels (11 μm^2^) were measured: one region in the nucleus, one in the cytoplasm and one outside any cells to assess the background intensity. The two ROIs in the cells were positioned at equal distance from the nuclear boundary. To normalize, the background intensity was subtracted from the two other intensities. Subsequently, the nuclear intensity was divided by the cytoplasmic intensity in order to find the nuclear-to-cytoplasmic ratio.

For quantification of FRAP (Fluorescent Recovery After Photobleaching) experiments, the integrated intensities were followed using MetaMorph (Molecular Devices). Three circular ROIs of the same size as the bleached ROI were followed: bleached ROI, one reporting ROI in the bleached nucleus and one ROI outside any cells to measure the background intensity. In parallel, the intensity of the whole field was measured to follow loss of intensity due to multiple and continual acquisition. To normalize, first the background intensity was subtracted from all other intensities. Then, nuclear intensities were normalized to the loss of intensity of the whole field over the entire experiment. Finally, they were normalized to their intensity in the first frame, prior to photobleaching. It is the recovery of this normalized intensity that was plotted and analyzed quantitatively (see Mathematical Modeling section).

For quantification of FLIP (Fluorescent Loss In Photobleaching) experiments, MetaMorph (Molecular Devices) was used to follow the integrated intensities of six different square ROIs of the same size as the bleached ROI: the bleached ROI, two reporting ROIs (one in the same compartment as the bleached point and one in the other compartment), two controls ROIs (one in the nucleus and one in the cytoplasm of one control cell) and one ROI outside any cells to measure the background intensity. The two nuclear ROIs and the cytoplasmic ROI of the bleached cells were positioned to be at approximately equal distances form one another. To normalize, first the background intensity was subtracted from the five others intensities measured in the cells. Then, the intensities measured in the cell of interest (three ROIs) were normalized by dividing by the average intensity of the two control ROIs. These data were plotted in the graphs. Our mathematical model – used to extract import rates, export rates and association rates - was based on the intensities of the whole cells rather than 6 individual ROIs (see Mathematical Modeling Methods). For all the image analyses using ROIs, the nucleoli regions, where YAP1 appeared to be excluded, were avoided.

#### Luciferase Assay

Luciferase assays were performed with the dual luciferase assay kit (Promega). Cells were lysed using passive lysis buffer. Lysates were placed into a white 96-well plate (Perkin Elmer) to assess Luciferase and Renilla activities using Envision Multilabel plate reader (Perkin Elmer). To normalize, the measurements of firefly luciferase activities were normalized to the renillla luciferase activities of the same sample.

#### ECM Remodeling Assay

To assess force-mediated matrix remodeling, 50 × 10^3^ fibroblasts were embedded in 100 μL of Collagen I:Matrigel (#354249: #354234; BD Biosciences) and seeded on a 35 mm glass bottom MatTek dish (P35-1.5-14-C, MatTek Co., Ashland, MA, USA). Once the gel was set, cells were maintained in DMEM + 10% FCS + 1% ITS, unless otherwise stated. Gel contraction was monitored daily by taking photographs of the gels. The gel contraction value refers to the contraction observed after 2 days. To obtain the gel contraction value, the relative diameter of the well and the gel were measured using ImageJ software, and the percentage of contraction was calculated using the formula 100 × (well diameter − gel diameter) / well diameter.

#### Western Blot and Immunoprecipitation

All protein lysates were obtained, processed and ran following standard procedures. Antibody description and working dilutions used can be found in Table S2. Immunoprecipitation was performed as follows: cells were lysed using buffer containing 50 mM HEPES at pH 7.5, 200 mM NaCl, 1 mM EDTA, 1% NP-40, 10 mM glycerophosphate, 50 mM NaF, 1.5 mM Na_3_VO_4_, protease inhibitor cocktail (Roche), 1 mM PMSF. Cell lysates were cleared by centrifugation for 10 min at 4°C and supernatants were used for immunoprecipitation. Total YAP1 was pre-incubated with protein-G conjugated Dynabeads for 1 h, followed by washing in 0.1%BSA/PBS and addition to 200ug of lysate for overnight incubation at 4°C. Immunoprecipitates were washed 3 times with lysis buffer, and proteins were eluted by boiling in SDS-PAGE sample buffer.

#### qRT-PCR

RNA isolation and arrays. RNA was isolated using RNeasy Kit (#74104, Qiagen) and cDNA made using Promega products (random primers: C1181, MMLV-RT: M3681, RNAse inhibitor: N2511) following standard procedures. All sequences used for PCR reactions are listed in Table S3.

### Mathematical Modeling and Model Validation

#### 1 Mathematical Methods

##### 1.1 FRAP Data Analysis

Estimation of the reactive and diffusive processes taking place during FRAP included analysis of both the postbleach intensity profile of the cell frame following the bleach process and the dynamic recovery of intensity in the Region Of Interest (ROI). Mathematical derivation leading to this methodology can be found in (Kang et al., 2009, Kang et al., 2010) and it explicitly accounts for rapid diffusion of the protein of interest over the timescale used for photobleaching. We briefly explain the main points of data fitting here. Methods for image acquisition and intensity normalization in specified ROIs are explained in Method Details.

###### 1.1.1 Postbleach Profile Analysis

The prebleach (the frame captured prior to the bleach process taking place) and postbleach (the frame immediately captured after completion of the bleach process) profiles were re-centred around the mid-point of the nominal bleach region (Figures S8A and S8B with the red circle in S8A illustrating the bleach region). An image mask of the nucleus minus the nucleoli regions was created by manually tracing the relevant boundaries using MATLAB's roipoly command with nucleoli most obvious from the prebleach profile (Figure S8C). This image mask enabled us to consider intensity changes only in the nucleus. Nucleoli were excluded because the dense chromatin packing excludes YAP1 and alters protein diffusion. Both the postbleach profile and image mask were transformed from Cartesian to polar coordinates using MATLAB's cart2pol function (Figures S8D and S8E). Data-points were then interpolated over the polar angle, *θ*, to account for the lower density of points near the centre of the nominal bleach region (Figure S8F). The median postbleach profile was then calculated, as the median intensity of all data-points within the image mask, for increasing radius from the nominal bleach-point (blue curve in Figure S8G). It was confirmed that an exponential of a Gaussian (red curve)

(Equation 1.1) $$C\left(r,0\right)=\;\exp\left[-K_{PB}\;\exp\left(-\frac{2r^{2}}{r_{e}^{2}}\right)\right]\text{for}\;r\geq 0$$

fit the median postbleach profiles well, where *r* is the radial distance from the origin, *r*_*e*_ is the effective radius (measure of distance along x-axis in S8G) and *K*_*PB*_ is the bleach-depth (measure of drop in intensity on y-axis in S8G). By minimizing the sum of squares due to error, the parameters *r*_*e*_ and *K*_*PB*_ for which Equation 1.1 best fits the data could be determined.

###### 1.1.2 Recovery Curve Analysis

Three possible model fits to the recovery curve, *S*(*t*), were then compared (Sprague et al., 2004). These were i) a diffusion model arising from either the mobile fraction being large (pure diffusion) or the on/off binding rates being fast relative to diffusion (effective diffusion); ii) a reaction-diffusion model that makes no assumptions on relative rates of binding and diffusion and iii) a reaction model where diffusion is assumed to be rapid and the recovery is determined by the slow reaction from immobile to mobile state. Each of the models thus incorporate some combination of rates of transfer from bound to unbound states (dissociation), rates of association from unbound to bound states (association) and rates of diffusion in bound and unbound states. These parameters are given respectively by *k*_*on*_, *k*_*off*_ and *D* for association, dissociation and diffusion.

###### Pure Diffusion and Effective Diffusion Models

In addition to being derived from the postbleach profile (1.1), the bleach depth can alternatively be calculated via the recovery curve intensity. Utilizing the point of completion of the bleach process, *S*(0) in the recovery curve, the corresponding bleach depth, *K*_*RC*_, solves the equation

(Equation 1.2) $$S\left(0\right)=\frac{\nu_{0}}{K_{RC}^{\nu_{0}}}\gamma \left(\nu_{0},K_{RC}\right)$$

where $\nu_{0}=r_{e}^{2}/r_{n}^{2}$, *r*_*n*_ is the nominal bleach radius i.e. the radius of the bleach region and *γ*(*ν*_0_,*K*_*RC*_) is the incomplete gamma function. The two parameters estimating bleach depth, *K*_*PB*_ and *K*_*RC*_, may not be exactly equal. For model fitting to the recovery curve, (Kang et al., 2009) recommend using the value derived from the recovery curve itself, for fitting consistency.

The diffusion function, *Q*_*D*_(*t*), fitted to the recovery curve *S*(*t*) is then given by

(Equation 1.3) $$Q_{D}\left(t\right)=\sum_{m=0}^{\infty }\frac{{\left(-K_{RC}\right)}^{m}r_{e}^{2}}{m!\left[r_{e}^{2}+m\left(8D_{e}t+r_{n}^{2}\right)\right]}$$

where *D*_*e*_, the only free parameter in this function is selected to minimize the weighted residuals (1.6) between this function *Q*_*D*_(*t*), and the recovery curve data, *S*(*t*). The diffusion coefficient is thus obtained explicitly via knowledge of the effective radius, *r*_*e*_, in the postbleach profile.

###### Reaction-Diffusion Model

For the reaction-diffusion model, in addition to the parameters required for the diffusion only model, two additional parameters are determined from the recovery curve: *f*_0_, the normalized initial postbleach intensity (a value between zero and one) and *R*, the mobile fraction of recovery, calculated as

(Equation 1.4) $$R=\frac{f_{oo}-f_{0}}{f_{i}-f_{0}}$$

where *f*_*oo*_ gives the mean intensity of the recovery curve data, once it has reached steady-state, and *f*_*i*_ gives the mean intensity of the recovery curve prior to bleaching (due to normalization, this value will be equal to or close to one). The reaction-diffusion function, *Q*_*RD*_(*t*), fitted to the recovery curve is rather more complex than the others described here (see (Kang et al., 2009) for further details). The MATLAB m-file, BDfrap.m, corresponding to the previous reference, was used to calculate these reaction-diffusion function values for varying *k*_*on*_, *k*_*off*_ and *D*_1_ (diffusion corresponding to the unbound state). We assumed that diffusion *D*_2_ corresponding to the bound state was 0*μm*^2^*s*^−1^ (supported by the FRAP data of H2B which does not diffuse - Figure S2C) to reduce the degrees of freedom in the model fit. The selection of the unknown parameters *k*_*on*_, *k*_*off*_ and *D*_1_ was then again made to minimize the weighted residuals between this function and recovery curve data.

###### Reaction Models

Reaction models were fitted as described in (Fritzsche and Charras, 2015), such that

(Equation 1.5) $$Q_{R_{n}}\left(t\right)=\sum_{i=1}^{n}A_{i}\left(1-\exp\left(-k_{off_{i}}\left(t-t_{0}\right)\right)\right)$$

where *n* = 1 for the single reaction and *n* = 2 for the double reaction. Here, *A*_*i*_ gives the amplitude for recovery, *k*_*offi*_ the corresponding rate of recovery and *t*_0_ allows the model fitting to account for noise in measurement at time zero of the recovery - the postbleach intensity.

###### Weighted Sum of Squares of Error

Without loss of generality, let the function, *Q*(*t*), refer to each of the reaction-diffusion based models described. Data fitting was then carried out to minimize the time-weighted residuals between the data, *S*(*t*), and model, *Q*(*t*), as described in (Kang et al., 2009) such that data-points for earlier time contribute more to the residual, allowing greater capacity to identify faster rates. We minimize the time-weighted sum of squares of error, given by

(Equation 1.6) $$SSE=\int_{0}^{\tau }\frac{{\left(s\left(\psi \right)-Q\left(\psi \right)\right)}^{2}}{\psi +\int_{0}^{\tau }S\left(\phi \right)d\phi }d\psi$$

where *τ* is the final point in time of the data and the integral in the denominator is included to remove the singularity at *t* = 0.

###### Implementation of Model Fitting

The nonlinear regression of each proposed model, *Q*(*t*), to the data, *S*(*t*), was carried out using the MATLAB algorithm nlinfit, found in the Statistics and Machine Learning Toolbox. The approach uses the Levenberg-Marquardt nonlinear least squares algorithm to find the fit that minimizes the weighted SSE (1.6). Initial parameter guesses are required and the algorithm then searches for the global minimum of the weighted SSE using these initial guesses as starting points. Poor initial guesses can lead to the algorithm becoming slower in reaching the global minimum or, even worse, becoming stuck in a local, rather than global, minimum. Care should therefore be taken in the choice of these initial guesses. Fortunately, the Levenberg-Marquardt approach is quite robust to poor initial parameter guesses, although it is then slower in reaching the minimum.

The diffusion parameter was initially guessed at 19*μm*^2^*s*^−1^. We estimated that EYFP-YAP1 has a volume roughly four times greater than a single GFP. We thus interpolated the rate of diffusion of three and five GFPs through the nucleus as estimated by (Baum et al., 2014). In order to provide good initial guesses for association and dissociation (and related amplitudes of recovery) for the models incorporating reaction dynamics, the exponential function

(Equation 1.7) y=α[1−exp(−κt)]

was fitted to each recovery curve whereby the fits for *α* and *κ* could be used as guesses for amplitude and association/dissociation for each curve. The function (1.7) is also nonlinear and so to derive *α* and *κ* we used the nlinfit algorithm and again needed initial guesses. For a small subsample of cells, a grid was constructed for the two parameters *α* and *κ* and the standard SSE calculated at each point on the grid. This identified the region of parameter space where the global minimum occurred as being *α* ≈ 0.3 and *κ* ≈ 0.5. For the fit of (1.7) to each curve we could then use α˜ = 0.3 and κ˜ = 0.5 as initial parameter guesses. The output values for *α* and *κ*, estimated from the nlinfit algorithm, were then used as initial guesses for various parameters in the models *Q*_*RD*_(*t*) and *Q*_*Rn*_(*t*).

For reaction-diffusion, *Q*_*RD*_(*t*), the initial guesses were given by $\left[\tilde{D},{\tilde{k}}_{on},{\tilde{k}}_{off}\right]=\left[19,\kappa ,\kappa \right]\text{.}$ In the case of the single reaction, *Q*_*R*1_(*t*), the initial guesses were given by $\left[{\tilde{A}}_{i},{\tilde{k}}_{off_{1}},{\tilde{t}}_{0}\right]=\left[\alpha ,\kappa ,0\right]\text{.}$ For the double reaction, the initial rates are estimated similarly to (Fritzsche and Charras, 2015). The fast reaction is assumed to contribute the first 30% of overall recovery and the slow reaction contributes the final 30% of recovery. We estimate where these regions occur based on the single reaction fit, *Q*_*R*1_(*t*) (due to it being a smooth curve as opposed to the original noisy data).

To determine the model that most likely describes the behavior of the system, we made use of the effective radius and bleach depth derived from the postbleach profile alongside a number of statistics that describe the model fitting to the recovery curve. The postbleach statistics implied that diffusion was likely to be fast and that we were in a regime where the observed recovery was dominated by slower reactions. From the model fitting to the recovery curves, we first ruled out certain model fits based on the magnitude of their rates being unrealistic. Diffusion model fits whereby the fitted diffusion parameter was fitted at over 60*μm*^2^*s*^−1^ were ruled out as erroneous as they approached, or exceeded, the measured diffusion rate in cells of a single GFP. A single GFP is approximately a quarter of the size of EYFP-YAP1 (see (Baum et al., 2014)). Reaction model fits in which the binding on and/or off rates were greater than 25*s*^−1^ were again determined to be erroneous. Such rates would be incredibly rapid and would not be able to be accurately estimated due to our frame-rate. Similarly, reaction models with dissociation rates less than 0.01*s*^−1^ (and 0.001*s*^−1^ for slower TEAD based experiments) were discarded as on the time-scale of our experiments, such a rate would reflect the model fitting erroneously picking up some possibly dubious linear trend in recovery that may not be representative of the true underlying processes. Following the discounting of models with implausible parameter fits, we compared the four model fits using the Akaike Information Criterion (AIC), a statistic that rewards goodness of fit but penalizes models based on the number of parameters required for that fit. From the AIC values for the four models, the Akaike weights (normalized relative likelihoods of each model) can be calculated. These Akaike weights can be interpreted as the probability that a certain model is most likely, given the data. Importantly, the AIC analysis does not say that a specific model is correct, it says only how likely the model is to be true in comparison to the other models. From the model fits that had not been ruled out due to erroneous parameter estimates, we used AIC to rank which was the most likely. A small number of fits were recorded as diffusion or reaction-diffusion. In cases where Akaike weights or implausible parameter values ruled out diffusion and reaction-diffusion models, the single and double reaction models were interpreted. Firstly, the Akaike weights for both the single and double reactions were compared. Secondly, an F-test comparing nested models was carried out on the two reaction models, with F-statistic given by

(Equation 1.8) $$F=\frac{\left(SSE_{1}-SSE_{2}\right)/\left(df_{1}-df_{2}\right)}{SSE_{2}/df_{2}}$$

with *df*_1_−*df*_2_ and *df*_2_ degrees of freedom. Here *SSE*_1_ and *SSE*_2_ are the time-weighted sum of squares of error (1.6) of the single and double reaction models respectively and *df*_1_ and *df*_2_ are the degrees of freedom of the single and double reaction models respectively. Significant p-values (≤0.05) alongside low Akaike weights for the single reaction implied a difference between the two models suggesting that a double reaction may be more likely than a single reaction. Non-significant p-values (*p* > 0.05) alongside Akaike weights for the single reaction of a similar magnitude to (or larger than) the double reaction suggested either that there was not enough evidence to assume a double reaction or strong evidence to assume a single reaction. In either case, a single reaction was assumed.

##### 1.2 FLIP Model Fitting

To model the FLIP data we developed a compartmentalized Partial Differential Equation (PDE) model that incorporated the bleach region, the rest of the nucleus and the cytoplasm (Figure S9A). The PDE contains one compartment corresponding to the nucleus and another corresponding to the cytoplasm. The two compartments are linked via flux boundary conditions.

###### 1.2.1 FLIP PDE Description

Within the nucleus we assume a single binding reaction

(Equation 1.9) $${\text{N}}_{\text{M}}+{\text{N}}_{\text{CHR}}\overset{{\tilde{k}}_{1}}{\underset{k_{-1}}{⇌}}\;{\text{N}}_{\text{I}}$$

where N_M_ represents unbound mobile proteins in the nucleus, N_CHR_ the chromatin binding partners such as TEAD and N_I_ the bound immobile state. We set *N*_*M*_(**x**,*t*), *N*_*I*_(**x**,*t*) and *N*_*CHR*_(**x**,*t*) to be the respective concentrations of N_M_, N_I_ and N_CHR_ at time *t* and location **x**=(*x*,*y*). Setting $k_{1}={\tilde{k}}_{1}N_{CHR}\text{,}$ we arrive at the general PDE reaction-diffusion model within the nucleus, given by

(Equation 1.10) $$\frac{\partial N_{M}}{\partial t}=D_{N}\nabla^{2}N_{M}-k_{1}N_{M}+k_{-1}N_{I},\;\;\left(\text{where}\;\nabla^{2}=\frac{\partial^{2}\;}{\partial x^{2}}+\frac{\partial^{2}\;}{\partial y^{2}}\right)$$

(Equation 1.11) $$\frac{\partial N_{I}}{\partial t}=k_{1}N_{M}-k_{-1}N_{I}\text{,}$$

reflecting our assumption that molecules in the bound immobile state, *N*_*I*_, do not diffuse and remain stationary. Bleaching occurs over a set region within the nucleus, which we describe with the reactions

(Equation 1.12) $${\text{N}}_{\text{M}}\overset{\eta }{\to }\emptyset \text{,}$$

(Equation 1.13) $${\text{N}}_{\text{I}}\overset{\eta }{\to }\emptyset ,$$

where the empty set, ∅, corresponds to molecules that have been bleached and effectively left our system and *η* gives the rate of bleaching.

Thus, in the nucleus, we have

(Equation 1.14) $$\frac{\partial N_{M}}{\partial t}=D_{N}\nabla^{2}N_{M}-k_{1}N_{M}+k_{-1}N_{I}-\eta \delta_{N,B}N_{M}\text{,}$$

(Equation 1.15) $$\frac{\partial N_{I}}{\partial t}=k_{1}N_{M}-k_{-1}N_{I}-\eta \delta_{N,B}N_{I}\text{,}$$

where the delta function is defined

(Equation 1.16) $$\delta_{N,B}=\{\begin{array}{cc} 1 & \text{at}\;\text{the}\;\text{bleachpoint}\\ 0 & \text{elsewhere}\text{.} \end{array}$$

Regions of nucleoli drastically affect the mobility of the molecule within the nucleus. They are densely packed, making it difficult for molecules to diffuse in or out of them. No clear YAP1 signal is observed in these regions. Hence, in our system we treat nucleoli as impenetrable by molecules and therefore impose zero-flux boundary conditions where the nucleus is in contact with these impenetrable islands. That is

(Equation 1.17) $${\frac{\partial N_{M}}{\partial x}|}_{x\in N_{int}},\;{\frac{\partial N_{M}}{\partial y}|}_{y\in N_{int}}=0$$

where **N**_**int**_ is the boundary between the nucleus and islands of nucleoli.

In the cytoplasmic compartment, we assume that the entire molecular population is mobile (observe the FRAP postbleach profiles and recovery times in Figures S3H–S3O) such that we obtain the single diffusive equation

(Equation 1.18) $$\frac{\partial C}{\partial t}=D_{C}\nabla^{2}C\text{.}$$

From here, we assume that the molecules diffuse at the same rate in both the nucleus and the cytoplasm such that *D* = *D*_*N =*_ *D*_*C*_. At the exterior boundary of the cytoplasm (the boundary of the cell) we impose zero-flux boundary conditions such that there is no concentration gradient at the boundary i.e.

(Equation 1.19) $${\frac{\partial C}{\partial x}|}_{x\in C_{ext}},\;\;{\frac{\partial C}{\partial y}|}_{y\in C_{ext}}=0$$

where **C**_**ext**_ is the cytoplasmic exterior.

Linking these two compartments is import and export. We can express this import/export between the two compartments via the reaction

(Equation 1.20) $${\text{N}}_{\text{M}}\overset{k_{2}}{\underset{k_{-2}}{⇌}}\;\text{C}$$

where it is assumed that only the mobile fraction in the nucleus can be exported and any molecules imported from the cytoplasm are mobile in the nucleus. These two compartments are linked via flux boundary conditions (Figure S9A) on the exterior of the nucleus (in contact with the cytoplasm) and the interior of the cytoplasm (in contact with the nucleus). In the case of the nucleus compartment, the outward flux is then proportional to *k*_2_**N**_**ext**_ where **N**_**ext**_ is the nucleus exterior and the inward flux is proportional to *k*_−2_**C**_**int**_ where **C**_**int**_ is the internal cytoplasmic boundary. For the case of the cytoplasmic compartment, the outward flux is then proportional to *k*_−2_**C**_**int**_ and the inward flux to *k*_2_**N**_**ext**_.

###### 1.2.2 Numerical PDE Fitting

Here we describe the main image extraction and data fitting techniques to fit the PDE described in FLIP PDE Description to our numerical data. Corresponding MATLAB scripts illustrating these procedures can be found at https://github.com/RobertPJenkins/FLIP-MATLAB-Skeleton-Scripts.

###### Numerical Implementation in MATLAB

We solved the compartmentalized PDE numerically in MATLAB. Finite differences were applied in the spatial domain to account for diffusion between grid-points in the same compartment and flux boundary conditions (import and export) between the nuclear compartment and cytoplasmic compartment. This reduced the PDE to a system of ODEs for each grid-point which were solved in the temporal domain using MATLAB's ode15s. The ODE solver, ode15s, for use in stiff problems (i.e. systems that include widely varying time-scales), was selected due to the fact that the solution has a region of very sharp change in concentration where it is not stiff, alongside regions of slowly changing concentration where it is stiff. The sharp change in concentration occurs most notably in and around the bleach-point at the initiation of the bleach process. The slowly changing concentration occurs both for the remainder of the cell far from the bleach-point, for all time, and the region in and around the bleach-point for longer time (when the majority of molecules within the cell have been bleached).

###### Image Discretization

To find the parameters in which the numerical solution to the PDE best fits our data we discretized the cell into coarse grid-points with each grid-point being the same dimensions as our bleach region, a 4.46*μm*^2^ square. The lattice of grid-points was initialised such that one of the grid-points overlapped exactly with the bleach-point (Figure S9B). The lattice size was selected to be more computationally efficient and more robust to erroneously defined flux boundaries between the nucleus and cytoplasm. Future iterations of the model fitting will attempt to fit a finer lattice, whilst controlling for the effects of increasing noise at the boundaries, in order to further refine parameter estimates.

The nuclear, nucleola and cytoplasmic boundaries were then determined manually, again using MATLAB's roipoly command. In the case of the cytoplasm, we excluded regions where the cell was very thin. For example, lamellipodia are much thinner than the focal section achieved by the microscope (200-300nm compared to >1 micron) and the low fluorescence intensity in these regions reflects the low volume of cytoplasm in the focal plane, not the differential dynamics or localization of the fluorescent protein. Typically, the cytoplasmic area selected for analysis was 1-2 times the area of the nucleus.

Image masks of both the nucleus, minus nucleoli, and the cytoplasm were then used to determine which grid-points were cytoplasmic and which were nuclear. Grid-points were defined as nuclear or cytoplasmic if at least 50% of that grid-point was occupied by the nuclear or cytoplasmic image mask (Figure S9C). The intensity at a given grid-point was then calculated as the mean intensity of all pixels within that grid-point. Only pixels that overlapped the nuclear or cytoplasmic masks were used in these calculations. This then defined our coarsely gridded cell with which we fit our PDE to (Figure S9D).

###### Implementation of Model Fitting

When fitting the model to the data, a number of parameters were required to be estimated: diffusion, *D*, association *k*_1_ and dissociation *k*_−1_, import *k*_−2_ and export *k*_2_, the rate of bleaching, *η* and the initial concentrations of the bound *N*_*I*0_ and unbound *N*_*M*0_ states in the nucleus and the cytoplasmic concentration *C*_0_. We fixed diffusion at *D* = 19*μm*^2^*s*^−1^ for all cases. This is an interpolation of the rate of diffusion of three and five GFPs through the nucleus reported in (Baum et al., 2014). We fixed the dissociation rate, *k*_−1_, to equal the median value for each cell-type derived from our FRAP analysis.

For the initial nuclear and cytoplasmic concentrations, we did not simply use the prebleach levels derived from the data, due to noise in acquisition. However, we were able to reduce the number of free parameters by fixing the initial concentrations in terms of each other. Prior to any bleaching having occurred, the concentrations of both the nuclear and cytoplasmic compartments were assumed to be homogeneously distributed. By reducing the spatial system to a simple system of Ordinary Differential Equations (ODEs) for concentration, the transfer between the two compartments described by the import/export reaction with bound/unbound states in the nucleus can naively be expressed as

(Equation 1.21) $$\frac{dN_{M_{0}}}{dt}=-\left(k_{2}+k_{1}\right)N_{M_{0}}+k_{-2}C_{0}+k_{-1}N_{I_{0}}\text{,}$$

(Equation 1.22) $$\frac{dN_{I_{0}}}{dt}=k_{1}N_{M_{0}}-k_{-1}N_{I_{0}}\text{,}$$

(Equation 1.23) $$\frac{dC_{0}}{dt}=k_{2}N_{M_{0}}-k_{-2}C_{0}\text{,}$$

where the subscripts in *N*_*M*0_, *N*_*I*0_ and *C*_0_ indicate that our system is in steady-state prior to perturbations due to bleaching. Thus, at this steady-state, we observe the relations between concentrations $N_{M_{0}}=k_{-2}C_{0}/k_{2}$ and $N_{I_{0}}=k_{1}N_{M_{0}}/k_{-1}$ and we fix concentrations in the nucleus in terms of reaction rates and cytoplasmic concentration, reducing the number of free parameters by two. Therefore, we estimated the parameters *k*_1_, *k*_2_, *k*_−2_, *η* and *C*_0_ by fitting our model to the data.

As with FRAP, the nonlinear regression of the compartmentalized PDE model to the data was carried out using the MATLAB algorithm nlinfit and we required initial parameter guesses for *k*_1_, *k*_2_, *k*_−2_, *η* and *C*_0_. For the initial cytoplasmic intensity, *C*_0_, we used the median intensity within our cytoplasmic region. For association rate, *k*_1_, our initial guess was set to be equal to the median dissociation rate acquired from FRAP for that cell type. For the final parameters, import, *k*_−2_, export, *k*_2_, and decay due to bleaching, *η*, we initially set up a simpler ODE based model that incorporated just these three mechanisms and fitted to data from a single reporting point in the nucleus and another in the cytoplasm. The single reporting point in the nucleus could be either the bleach-point or another location.

This simple ODE model given by

(Equation 1.24) $$\frac{dN}{dt}=-k_{2}N+k_{-2}C-\eta N\text{,}$$

(Equation 1.25) $$\frac{dC}{dt}=k_{2}N-k_{-2}C\text{,}$$

allowed us to get a naive idea of the magnitudes of import, export and decay due to bleaching via analysis of quality of fit for multiple cells. Hence we made initial guesses of export and import of around 0.002*s*^−1^ to 0.005*s*^−1^, with the nuclear-to-cytoplasmic ratio helping to inform on which of the two should be initially guessed to be larger. A good initial guess for the bleaching decay was found to be 1.5*s*^−1^, via model fitting analysis of both this simpler ODE model and our full compartmentalized PDE model.

###### Weighted Sum of Squares of Error

Data fitting was carried out to minimize spatial-time-weighted residuals between the data and model. As with FRAP, data-points for earlier time contribute more to the residual. The data-points were spatially weighted such that the bleach-point, entire nucleus minus the bleach-point and entire cytoplasm were equally weighted with each other. This avoided the critical bleach-point and, to a lesser extent, the nucleus surrounding the bleach-point having a low influence on the overall model fit due to the larger size of the rest of the cell. Within the nucleus excluding the bleach-point and cytoplasm, each grid-point was also weighted equally with all other grid-points in that given compartment. More formally, let the signal at a given grid-point *i*, *j* and compartment *k* (where *k* = *B* for the bleach-point, *k* = *N* for grid-points in the nucleus excluding the bleach-point and *k* = *C* for grid-points in the cytoplasm) be given by *S*_*i*,*j*,*k*_(*t*) for time-point *t*. We set $S_{i,j,C}\left(t\right)=0\;\forall \;i,j\notin C,\;S_{i,j,N}\left(t\right)=0\;\forall \;i,j\notin N$ and $S_{i,j,B}\left(t\right)=0\;\forall \;i,j\notin B\text{.}$ To account for both the time-weighting and equally weighted grid-points in each compartment we set

(Equation 1.26) $$w_{i,j,k}\left(\psi \right)=\{\begin{array}{cc} \frac{1}{\left[\psi +\sum_{i,j,k}\int_{0}^{\tau }S_{i,j,k}\left(\phi \right)d\phi \right]\left\{\int_{0}^{\tau }S_{i,j,k}\left(\phi \right)d\phi \right\}} & S_{i,j,k}\left(t\right)>0\;\text{for}\;\text{some}\;t\text{,}\\ 0 & \text{else}, \end{array}$$

where *τ* is the final point in time of the data. The function in square brackets in the denominator is time-weighted, as in FRAP, except the integral to remove the singularity at *t* = 0 is summed over all grid-points. The integral in the curly brackets in the denominator ensures each grid-point within a compartment has equal weighting on the best-fit by normalizing by the total intensity, over all time, of the signal at that grid-point. Finally, the grid-points are re-weighted such that the bleach-point, entire cytoplasm and entire nucleus minus the bleach-point all have equal weighting with each other. Let

(Equation 1.27) $$W_{N}=\sum_{i,j}\int_{0}^{\tau }w_{i,j,N}\left(\psi \right)d\psi \text{,}$$

i.e. the total sum of weights of all grid-points in the nucleus for all time. Similarly, let

(Equation 1.28) $$W_{B}=\sum_{i,j}\int_{0}^{\tau }w_{i,j,B}\left(\psi \right)d\psi ,\;\text{and}\;W_{C}=\sum_{i,j}\int_{0}^{\tau }w_{i,j,C}\left(\psi \right)d\psi$$

for the bleach-point and cytoplasm. Then ${\tilde{w}}_{i,j,N}\left(\psi \right)=w_{i,j,N}\left(\psi \right),\cdot {\tilde{w}}_{i,j,B}\left(\psi \right)=w_{i,j,B}\left(\psi \right)W_{N}/W_{B}$ and ${\tilde{w}}_{i,j,C}\left(\psi \right)=w_{i,j,C}\left(\psi \right)W_{N}/W_{C}\text{,}$ such that the nucleus minus the bleach-point, the bleach-point and the cytoplasm all confer equal weighting on the overall fit. We then seek to minimize

(Equation 1.29) $$SSE=\sum_{i,j,k}\int_{0}^{\tau }{\left(S_{i,j,k}\left(\psi \right)-G_{i,j,k}\left(\psi \right)\right)}^{2}{\tilde{w}}_{i,j,k}\left(\psi \right)d\psi$$

where *G*_*i*,*j*,*k*_(*ψ*) is the solution to the compartmentalized PDE at the relevant spatial grid-point and time-point.

#### 2 Sensitivity Analysis

##### 2.1 FRAP

###### 2.1.1 Postbleach Profile

Figure S10A provides a heatmap of Sum of Squares due to Error (SSE) of the fit of Equation 1.1 to the data as the bleach depth, *K*_*PB*_, and effective radius, *r*_*e*_, are varied. The heatmap demonstrates that our choice of parameters are at the global minimum.

###### 2.1.2 Recovery Curve

Figure S10B provides the SSE for Equation 1.2 fitted to the recovery curve as the bleach depth, *K*_*RC*_, is varied. Once again, the plot demonstrates our parameter fit occurs at the global minimum. Finally, in Figure S10C we plot the heatmap of SSE of the fit of a single exponential reaction as the amplitude and rate of reaction are varied. Again, we find our choice of parameters occurs at the global minimum, although there does exist a shallower gradient of increasing SSE for increasing dissociation rate.

###### 2.1.3 The Zero Import/Export Assumption in FRAP

One of the assumptions in our FRAP model fitting was that import and export could be assumed to be zero on the experimental timescale for recovery. This assumption was justified via experimental observation. However, we also attempted to quantify what effect this assumption had on our estimated rates of recovery in FRAP. To do so we turned to a simplified FLIP model where there was no decay due to bleaching. The model thus consisted of association, dissociation and diffusion in the nuclear compartment, diffusion in the cytoplasmic compartment and import and export between the two compartments. By setting import and export to zero, the model reduces to a simple model of reaction diffusion in the nucleus and diffusion in the disconnected cytoplasm. We attempted to use FLIP to simulate the recovery of the fluorescent signal at the bleach-point in the nucleus immediately following bleaching, both in the presence and absence of import/export.

Taking the nuclear and cytoplasmic boundary data from FLIP model fitting, we quantified the average area and eccentricity of the nucleus and cytoplasm in CAF1 EYFP-YAP1_WT (CAF1_WT) cells. With this, we generated a typical cell to simulate. For initial conditions, the protein concentration was set to one in the nucleus and the corresponding concentration in the cytoplasm for a given cell-type found via the steady-state solutions to Equations 1.21, 1.22, and 1.23. The concentration of protein around the bleach-point would be lower, following Equation 1.1 with nominal radius selected to be 1.232 microns and effective radius and bleach depth being the corresponding average values from the FRAP postbleach profile analysis for a given cell-type. We set the bleach-point to either the centre of the nucleus or right-shifted and closer to the nuclear-cytoplasmic boundary (Figure S10D).

The FLIP model is not designed to analyse the FRAP recovery and so will only be a rough approximation. To account for this, firstly, assumptions have to be made about the proportions of the mobile and immobile fractions at time zero. The bleach-depth affects the assumptions about the fraction of mobile and immobile protein. The total mobile concentration at time zero has to be assumed the same across the whole nucleus. The maximum mobile concentration possible is then limited by the magnitude of the reduced protein concentration at the bleach-point. Otherwise, if the concentration of mobile protein was higher further from the bleach-point, mobile proteins would rapidly diffuse into the bleach-region leading to a diffusive recovery rather than a reactive one. Secondly, to force the system into a reactive recovery, diffusion is set to be rapid at 1000*μm*^2^*s*^−1^.

From these postbleach initial conditions, the FLIP model can simulate the recovery of the bleach region both in the presence and absence of import/export over the timescale of our FRAP experiments (≈15*s*). The difference in recovery between these two models at the bleach-point gives an indication of the effects of ignoring import/export on our estimates of dissociation. Fitting the single reaction FRAP model (1.5) to each of these FLIP approximations yields estimates of the rate of recovery in each case (Table S4). There is a marginal increase in the total recovery in models with import/export included in comparison to the case of import/export excluded. This is due to mixing with the cytoplasm. This results in the model assuming zero import/export hitting steady-state earlier and thus predicting a faster rate of recovery. Thus, when assuming import/export is zero, we underestimate the rate of dissociation since we are in fact fitting to the recovery curve that also includes the marginal effects of import/export. However, the ratio of the two rates of recovery shows that in most cases, the absence of import/export in the FRAP model is having less than a 5% effect on the estimated dissociation rate. The location of the bleach-point also only has a marginal effect on estimated dissociation rate, although bleach-points closer to the nuclear-cytoplasmic boundary appear to be estimated more exactly. The faster the estimated rate of dissociation, the greater the relative error in estimate of dissociation. The greatest effect is thus on NF1_S94A and CAF1_S94A, presumably due to the increased turnover in mobile-immobile state causing greater levels of mixing between the nucleus and cytoplasm. This effect is reduced for bleach-points closer to the nuclear-cytoplasmic boundary.

Figure S10E compares the recovery for various cell-types assuming import/export is zero (black) or non-zero (blue). The red dot-dash line corresponds to the exponential recovery (1.5) with dissociation rate corresponding to FRAP results and remaining parameters fitted to the non-zero import/export recovery curve. The results demonstrate the close agreement between the three recoveries.

##### 2.2 FLIP

We have carried out a sensitivity analysis over six cells, three NF_WT and three CAF_WT to analyze how robust our association, import and export parameter fits are to noise in our estimates for the dissociation rates (acquired from FRAP) and the rate of diffusion estimated from (Baum et al., 2014). Here we illustrate our findings via a single NF1_WT and CAF1_WT. Our initial analysis focusses on the sensitivity of the five free parameters, *k*_1_, *k*_2_, *k*_−2_, *η* and *C*_0_ to the dissociation rate and diffusion rate, both fixed in the FLIP model.

The dissociation rate was acquired via FRAP analysis of each cell. However, we used a static value that does not account for intercellular variation. We began the sensitivity analysis by varying the dissociation rate and seeing how this affected the fitting of the other parameters. Predictably, the association rate is most sensitive to the value of the dissociation rate. This is due to the rate of diffusion being fixed and thus the spread of the bleach around the nucleus is reliant on the proportion of the molecule that is mobile at any point in time. For smaller *k*_*off*_ (i.e. a value twice the fitted experimental value or below) the relationship is nonlinear with the association rate decreasing faster than the association rate to maintain data fit (Figures S11A and S11E). This suggests that when the time for the molecule to dissociate from the bound state is set to be too long, the association rate needs to be even slower to allow for a greater pool of mobile protein at any point in time. For larger *k*_*off*_ (i.e. greater than two times the experimental rate) the relationship becomes linear. However, for fast dissociation rates, the association rate increases about three times as fast to maintain data fit. This is due to the reverse of the above: when molecules are set to dissociate too fast, then the association rate must increase faster to allow for a greater immobile pool at any point in time. This is a consequence of the fact that the rate of diffusion is fixed. The cytoplasmic intensity, bleach decay rate and import from the cytoplasm are not sensitive to the rate at which dissociation is fitted. The rate of export is sensitive to rate of dissociation although much less so than the association rate. The export rate is sensitive only for dissociation rates that have been fitted too slow. For slow dissociation, the export rate decreases below the experimentally fitted value. This is most likely a consequence of the proportion of mobile protein increasing (see above). As the dissociation rate increases, the mobile fraction decreases and thus the export rate must increase to maintain the correct levels of compartment mixing and fit to the data.

We then considered dissociation fixed (at 0.55*s*^−1^ for NFs and 0.40*s*^−1^ for CAFs) and observed the effects of varying the fitted value of diffusion on the other parameter fits (Figures S11B and S11F). All parameters are more sensitive to diffusion that has been fitted as very slow compared to our estimate, than to other rates of fitted diffusion. Since we have already observed that diffusion must be fast, this suggests we are in a region of parameter space where parameter fits are less sensitive to the fitted rate of diffusion. Predictably once again, the association rate is most sensitive to the fitted rate of diffusion. For low fitted rates of diffusion, the association rate tends to zero implying that for the model to fit well, the entire molecular population must be mobile. For faster fitted rates of diffusion, the association rate increases, implying a smaller mobile fraction. The association rate tends to a constant for fast fitted diffusion, demonstrating that for rapid diffusion, the association rate is not sensitive to changes in diffusivity. The effects on export rate are similar to those of association rate but export is not as sensitive to fitted diffusion rate as association rate is. A low fitted diffusion rate implies the mobile fraction must be large to account for the spread within the nucleus. This in turn requires a reduction in the export rate to fit the global nuclear and cytoplasmic concentrations. The reverse is then true for fast fitted diffusion rates. That is, export rate is dependent on the diffusion rate only via the association rate. The import, decay rate and cytoplasmic intensity are insensitive to the fitted value of diffusion, apart from being mildly sensitive at very low fitted rates of diffusion. This possibly reflects the model assumptions beginning to break down.

Following from this we fixed all the parameters to their experimentally fitted values and varied the association rate and export rate simultaneously. We calculated the weighted-SSE for each fit, generating a heat map of error of fit versus association rate and export rate (Figures S11C and S11G). The results clearly demonstrate that our fitted parameters for association rate and export occur at the global minimum.

Similarly we fixed all parameters at their fitted values except for import and export. The heatmaps of weighted-SSE for import/export (Figures S11D and S11H) demonstrate that although we are at the global minimum, it is shallower than the association rate/export rate equivalent. The heatmap demonstrates a linearity in relationship between import and export qualitatively similar to the relationship between association and dissociation rates. This linearity reflects the ratio of nuclear-to-cytoplasmic intensity within the cell. However, the nucleus and cytoplasm have differing levels of YAP1 on the flux boundary between the two compartments and these levels change over time. Only the unique global minimum truly reflects these dynamically shifting values on the boundary.

Figure S11I gives a horizontal linescan of the FLIP mathematical model output for the CAF cell shown in Figure S4B at various time-points. The results demonstrate the spatial stability of the PDE solution. The solution is relatively flat in the cytoplasm. In the nucleus the solution is flat at the boundaries with the cytoplasm and smoothly decreases towards the sink at the bleach-point. Between the nucleus and cytoplasm there is a discontinuous jump in intensity between the two compartments.

#### 3 Residual Analysis

Residual analysis has been carried out for both the FRAP and FLIP model fitting (Figure S12).

Residual analysis (observed value minus predicted value) for FRAP was carried out on the model fits for pure/effective diffusion (S12A), reaction-diffusion (S12B), single reaction only (S12C) and double reaction only (S12D) on the recovery curves of 30 NF_WT (10 of each bleach radius). In each case, the red line represents the median residual of all 30 NF_WT cells at each time-point. The figures demonstrate that the pure-diffusion model is inappropriate to describe the recovery as it significantly underestimates the short-time recovery and significantly overestimates the long-time recovery. However, the residuals of the reaction-diffusion, single reaction only and double reaction only models demonstrate no obvious functional behavior, being distributed normally around zero, suggesting that each of these models describes the data well. With this in mind, the simplest system that describes the data best may be most appropriate, in this case the single reaction only model. This is in agreement with the AIC and F-test analysis (Table S1 and Recovery Curve Analysis above).

Residual analysis of FLIP of a single cell (Figure S12E) demonstrates no obvious functional link between the residuals and spatial region of a cell, suggesting that the model can correctly describe the appropriate spatial aspects of the system. We then considered 15 CAF_WT cells and extracted the residuals versus time for the bleach-point (Figure S12F), remainder of the nucleus (S12G) and the cytoplasm (S12H). The results suggest that the model slightly overestimates the rate of decay due to bleaching (S12F) and as a consequence, overestimates the long-time behavior at the bleach-point. The residuals at the remainder of the nucleus and cytoplasm are generally distributed around zero. The more sparse outlier regions of residuals in Figures S12G and S12H could illustrate erroneous boundary determination between the nucleus and cytoplasm such that some grid-points defined as nuclear are cytoplasmic and vice-versa. This effect would become more pronounced if there was significant cell movement. Again, for small time, the residuals do hint at the model overestimating the rate at which YAP1 leaves the system due to bleaching. This could be a consequence, for example, of an unaccounted for additional reaction that reduced the overall motility of YAP1 in the nucleus. Overall though, the residual analysis suggests that the FLIP model is a good fit.

#### 4 Correlation of Cellular Morphology and Protein Dynamics

##### 4.1 Import and Export versus Cellular Morphology

FLIP data was used to correlate import and export with the nuclear morphology (Figures 5A, 5B, and S5A–S5D). Using the nuclear and cytoplasmic boundary information acquired during FLIP model fitting, the function regionprops, in MATLAB's Image Processing Toolbox, was used to quantify various nuclear morphologies for each cell at time zero. Circularity was calculated from metrics defined in regionprops, as *f*_*circ*_ = 4*πArea*/*Perimeter*^2^ where the circularity of a circle is 1 and lower for other shapes. Some of the values of *f*_*circ*_ were calculated to be greater than one as a consequence of the way MATLAB numerically estimates a shape's perimeter. The nuclear-to-cytoplasmic ratio was calculated at time zero by taking the ratio of the median intensities for each of the defined regions. Pearson correlations and corresponding p-values testing the hypothesis of zero correlation versus the alternative of non-zero correlation were calculated using MATLAB's corr function.

##### 4.2 Analysis of Dynamic Nuclear-to-Cytoplasmic Ratio, Cellular Morphology and Motility

Cell tracking of Videos (Videos S8 and S9) was carried out in order to quantify how dynamic changes in nuclear and cytoplasmic morphologies, cell speeds and nuclear-to-cytoplasmic ratios correlate with each other (Figures 5C–5I and S5E–S5H). The cell tracking was carried out using a custom written algorithm in MATLAB. The nuclear and cytoplasmic channels were thresholded on a frame by frame basis and morphological opening operations carried out. Area thresholding was carried out to remove noise. Morphological erosion followed by a distance transform was carried out on the fibroblast channel in order to separate shallowly touching fibroblasts into separate cells. The algorithm to separate cytoplasmic objects then worked in two stages. Initially, on a frame by frame basis where objects were labelled, reclassified or removed from just the single frame under consideration. Following the frame processing stage, cells were then relabelled such that each tracked cell was labelled the same throughout the cell tracking process in a frame joining phase.

In the frame processing stage, distance transforms were frequently employed. This allowed single cytoplasmic objects to be broken into multiple cells, based on the distance of pixels in that object to nuclei or previously defined overlapping cells in a neighbouring frame. For each frame, single cytoplasmic objects composed, in fact, of multiple cells, were detected and either transformed back into multiple cells in straightforward cases or removed otherwise. This detection and transformation was based on overlapping cytoplasmic objects in the previous frame (that would be well defined as individual cells), overlapping nuclear objects in the current frame and the distance transforms of these objects. Cell division was accounted for by splitting single cytoplasmic objects that overlapped with two nuclei, based on a distance transform of the nuclei. Cytoplasmic objects with no correpsonding nucleus in a given frame were removed from that single frame. Cytoplasmic objects that touched the boundary of the the frame were removed in that frame, as morphological metrics could not be quantified accurately. Cells that were removed in a single frame could be tracked as a new cell in the subsequent frames.

In the frame joining phase, overlapping cytoplasmic objects in consecutive frames were given the same label. Cytoplasmic objects were relabelled such that they had the same label as the cytoplasmic object, overlapping from the preceding frame. Care was taken to account for the fact that not all overlaps would have one-to-one correspondence, some had one-to-many and others many-to-one. Objects that had no overlap with objects from the previous frame (due to object removal in the frame processing stage or noise) were given a new identity label. Objects that could not be tracked successfully for two hours or more (at least 24 frames) were then removed.

The overall process removed cells where we could not confidently determine their cell boundaries and dynamic data was recorded only for cells that could be tracked for at least two hours, making the dynamic analysis of these cells quite robust.

The morphological properties of both the nucleus and cytoplasm of each cell were calculated at each timepoint using MATLAB's regionprops command in the Image Processing Toolbox and circularity defined as in the section on Import and Export versus Cellular Morphology. Again, this measure suffers from the same numerical perimeter approximation issues that led to cases of circularity greater than one. The median intensities of each compartment at each timepoint were recorded to provide the nuclear-to-cytoplasmic ratio. The derivatives of all of these properties alongside cell speed and cell acceleration were also calculated.

The mean-value was subtracted from each signal and autocorrelations and cross-correlations calculated, on these resultant signals using the xcorr function in MATLAB's Signal Processing Toolbox. The results were normalised such that Autocorrelation Functions at zero lag equal one (and all signals fall between plus and minus one). The mean subtraction and normalisation procedure results in close agreement between the cross-correlations at zero lag and Pearson correlation. When carrying out correlation between signals of different length (raw signal, first derivatives and second derivatives) signals were interpolated.

### Quantification and Statistical Analysis

Statistical analyses were performed using Prism software (GraphPad Software). p values were obtained using Mann-Whitney unpaired t–test, with significance set at p < 0.05. Graphs show symbols describing p values: ^∗^, p < 0.05; ^∗∗^, p < 0.01; ^∗∗∗^, p < 0.001, ^∗∗∗∗^, p<0.0001. Unless otherwise stated, box plots show the median, upper and lower quartiles, and maximum and minimum values.

For all localization, FRAP, and FLIP data the experimental unit was considered as the individual cell. The figure legends state the number of cells used in the data presented. In addition, the number of different experiments from which the cell measurements were collated is stated. For gene expression analysis (QRT-PCR and luciferase assays), the experimental unit was considered as the plate or well. Technical replicates were performed either by splitting the material from a single or setting up duplicate wells. Biological replicates were performed across different days and weeks. The figure legends state both the number of technical and biological replicates.

### Data and Software Availability

The core modules of the MATLAB code are available at https://github.com/RobertPJenkins/FLIP-MATLAB-Skeleton-Scripts. Implementation will require user specific modification to account for the precise imaging context and acquisition settings. The full code is available on request.

## Supplemental Information

Document S1. Figures S1–S12 and Tables S2–S4

Table S1. Table Compiling Output of Mathematical Models Fitting Experimental Data for Nuclear FRAP of NF1 and CAF1 Expressing EYFP-YAP1, Related to Figure 2

Video S1. Nuclear FRAP of CAF1 Expressing H2B-GFP and EGFP, Related to Figure 2 Scale, 4 μm.

Video S2. Nuclear FRAP of NF1 and CAF1 Expressing EYFP-YAP1, Related to Figure 2 Scale, 4 μm.

Video S3. Cytoplasmic FRAP in NF1 and CAF1 Expressing EYFP-YAP1, Related to Figure 2 Scale, 4 μm.

Video S4. Nuclear FRAP of NF1 and CAF1 Expressing Either EYFP-YAP1_5SA or EYFP-YAP1_S94A, Related to Figure 3 Scale, 4 μm.

Video S5. Nuclear FRAP of NF1 and CAF1 Expressing TEAD1-mCherry, Related to Figure 3 Scale, 4 μm.

Video S6. FLIP of NF1 and CAF1 Expressing EYFP-YAP1, Related to Figure 4 Scale, 10 μm.

Video S7. FLIP of NF1 and CAF1 Expressing Either EYFP-YAP1_5SA or EYFP-YAP1_S94A, Related to Figure 4 Scale, 10 μm.

Video S8. Representative Videos of NF1 EYFP-YAP1_WT and H2B-Turquoise (Nuclear Marker) Cell Lines Used for Analysis in Figures 5 and S5, Related to Figure 5 Scale, 50 μm.

Video S9. Representative Videos of NF1 EYFP-YAP1_WT and H2B-Turquoise (Nuclear Marker) Cell Lines Used for Analysis in Figures 5 and S5, Related to Figure 5 Scale, 50 μm.

Video S10. FRAP of CAF1 Expressing EYFP-YAP1_Y357F or EYFP-YAP1 Treated with 100 nM Latrunculin B and 300 nM Dasatinib, Related to Figure 6 Scale, 4 μm.

Video S11. FLIP of CAF1 Expressing EYFP-YAP1_Y357F or EYFP-YAP1 Treated with 100nM Latrunculin B and 300 nM Dasatinib, Related to Figure 6 Scale, 10 μm.

Data S1. MATLAB FLIP Model Fitting Scripts, Related to STAR Methods Skeleton MATLAB scripts illustrate the image processing and FLIP PDE nonlinear model fitting code used to analyze FLIP image data. (A) Image processing and PDE model fitting MATLAB script includes example code used to convert the cell to a coarse PDE, extract the spatial intensity profile and nonlinearly fit the system of PDEs to these data. (B) FLIP PDE MATLAB Script demonstrates how to build up a system of PDEs to fit to the experimental data. The full code is available on request.

Document S2. Article plus Supplemental Information
