# Supplementary material for: Quantitative Analysis Reveals that Actin and Src-Family Kinases Regulate Nuclear YAP1 and Its Export
Source: Cell Syst. 2018 Jun 27;6(6):692–708.e13. doi: 10.1016/j.cels.2018.05.006 (PMC6035388; doi:10.1016/j.cels.2018.05.006)
Supplement: Document S1. Figures S1–S12 and Tables S2–S4 [file mmc1.pdf]

Cell Systems, Volume *6*

Supplemental Information

Quantitative Analysis Reveals that Actin and Src-Family Kinases Regulate Nuclear YAP1 and Its Export

Nil Ege, Anna M. Dowbaj, Ming Jiang, Michael Howell, Steven Hooper, Charles Foster, Robert P. Jenkins, and Erik Sahai

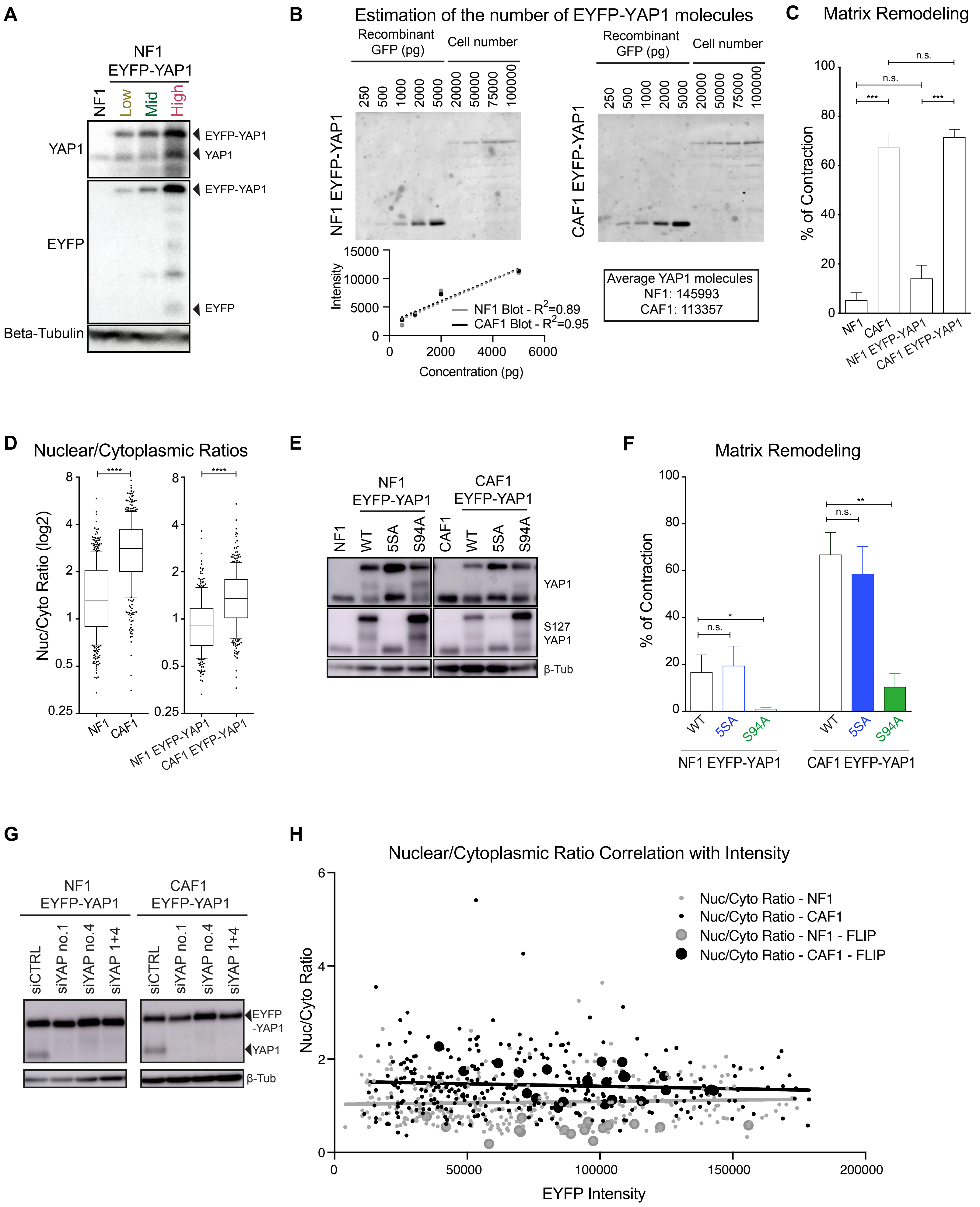

Supplemental Figure S1: Overexpression of EYFP-YAP1 maintains fibroblasts phenotype. Related to Figure1: Legends next page.

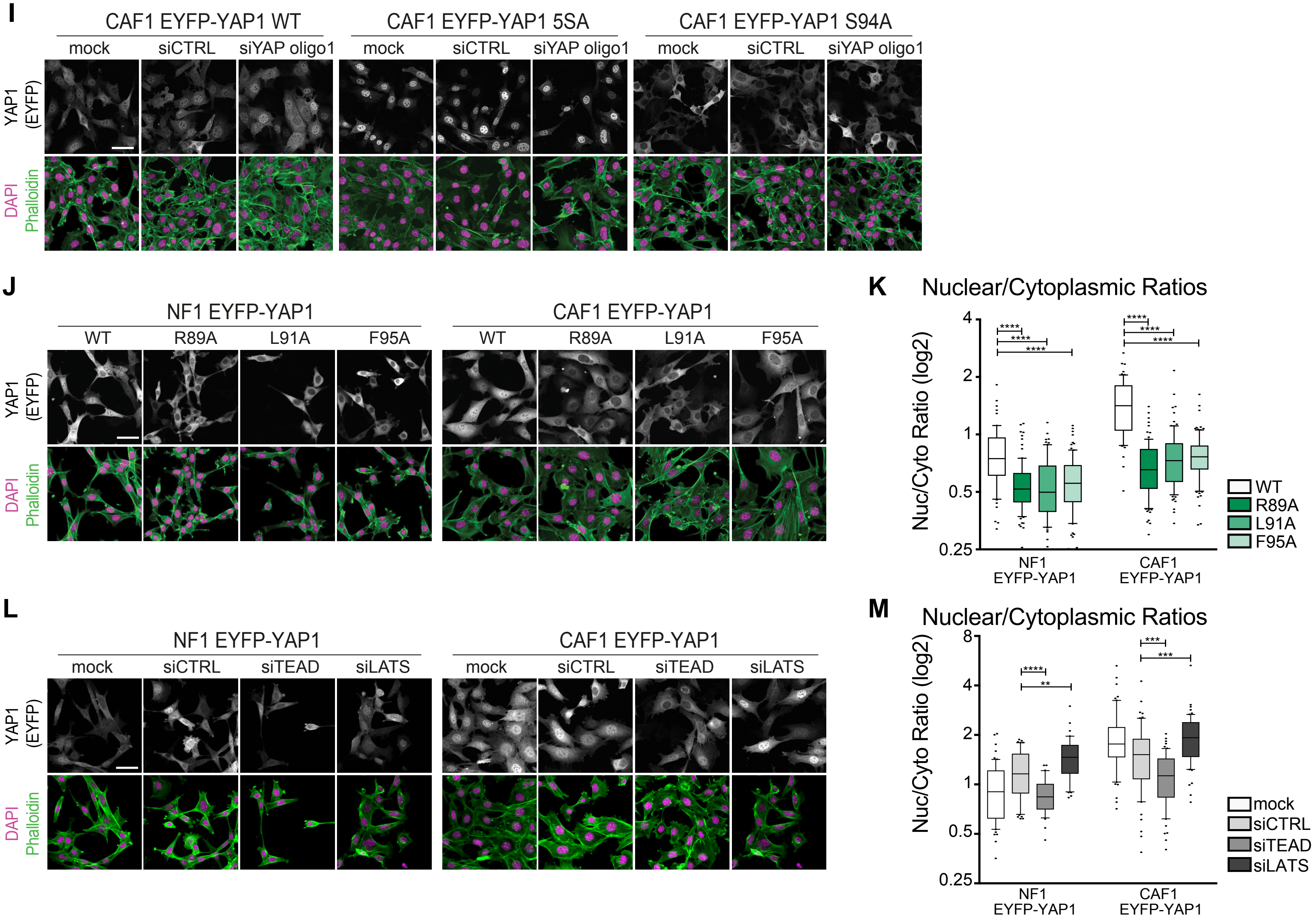

Supplemental Figure S1: Overexpression of EYFP-YAP1 maintains fibroblasts phenotype. Related to Figure1: (**A**) Western blot showing the expressions of EYFP-YAP1 (92kDa) versus endogenous YAP1 (61kDa), in NF1 WT and in three different FACS-sorted populations (Low-Mid-High) of NF1 EYFP-YAP1. EYFP (27kDa) and Beta-Tubulin (42kDa) are also presented. (**B**) Western Blot and linear regression analyses to assess the number of EYFP-YAP1 molecules in both NF1 and CAF1, using different concentration of GFP recombinant proteins. (**C**) Contraction assay of WT and EYFP-YAP1 expressing NF1 and CAF1 in normal media (NM). Bars represent mean ± s.e.m. of 5 independent experiments. (**D**) Box-plot (10&90) of nuclear-to-cytoplasmic ratio (log2 scale) of endogenous YAP1 (left) and EYFP-YAP1 (right) in NF1 and CAF1. n>170 cells for each condition from at least 3 independent experiments. (**E**) Western Blot showing the expressions of EYFP-YAP1_WT, 5SA, S94A (92kDa) versus endogenous YAP1 (61kDa), in NF1 and CAF1. Phosphorylation of Serine 127 and Tubulin (42kDa) are also presented. (**F**) Contraction assay of EYFP-YAP1, EYFP-YAP1_5SA and EYFP-YAP1_S94A expressing NF1 and CAF1. Bars represent mean ± s.e.m. of 5 independent experiments. (**G**) Western blot showing the expression of EYFP-YAP1_WT (92kDa) and endogenous YAP1 (61kDa) after the siRNA depletion of endogenous YAP1 protein. Beta-tubulin (42kDa) loading control is also presented. (**H**) Scatterplot showing the relative expression level of EYFP-YAP1 and its nuclear-to-cytoplasmic distribution. Grey dots are NF1 data points and black dots are CAF1 data points – in both cases larger dots indicate data from cells used in the FLIP analysis in Figure4. Horizontal trend lines indicate that expression level is not correlated with localization (linear regression slope non-significant, Pearson and Spearman correlation coefficients non-significant). (**I**) Representative images of EYFP-YAP1 localization in CAF1 EYFP-YAP1_WT, CAF1 EYFP-YAP1_5SA and CAF1 EYFP-YAP1_S94A after siRNA knockdown of endogenous YAP1. Scale bar, 50µm. (**J**) Representative images of EYFP-YAP1 localization in NF1 and CAF1 cell lines expressing EYFP-YAP1_WT vs TEAD-binding mutants EYFP-YAP1_R89A, EYFP-YAP1_L91A, and EYFP-YAP1_F95A. Scale bar, 50µm. (**K**) Boxplot (10&90) of nuclear-to-cytoplasmic ratio (log2 scale) corresponding to quantification of (J). n>90 cells for each condition from at least 3 independent experiments. (**L**) Representative images of EYFP-YAP1 localization in NF1 and CAF1 cell lines expressing EYFP-YAP1_WT after siRNA knockdown of TEAD1-4 or LATS1-2. Scale bar, 50µm. (**M**) Box-plot (10&90) of nuclear-to-cytoplasmic ratio (log2 scale) corresponding to quantification of (L). n>36 cells for each condition from at least 2 independent experiments. Mann-Whitney U-test, n.s., non-significant, * p≤0.05,** p≤0.01, *** p≤0.001 **** p≤0.0001.

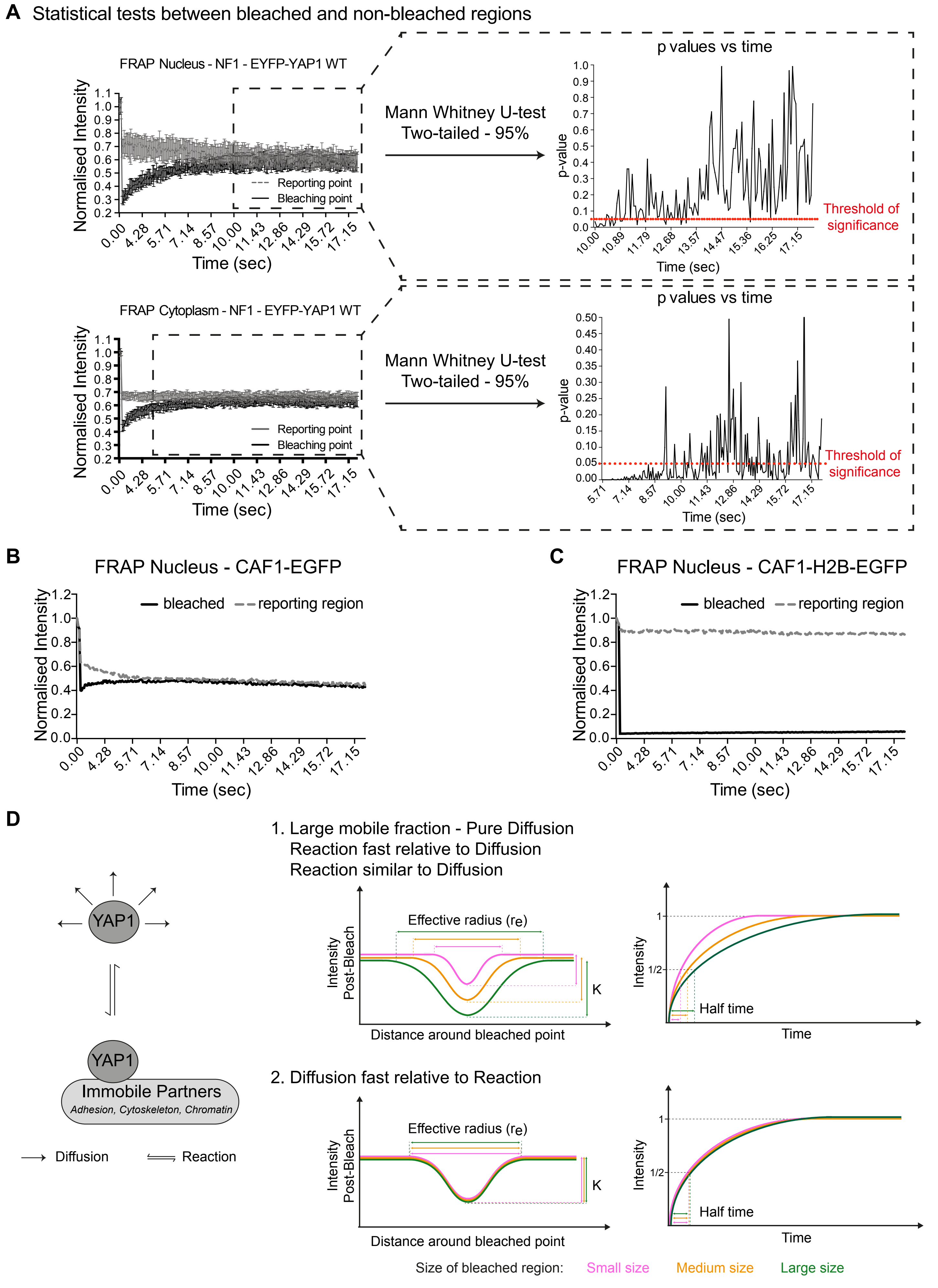

Supplemental Figure S2: FRAP experiments to identify diffusion and nuclear dissociation rates. Related to Figure2: Legends next page.

Supplemental Figure S2: FRAP experiments to identify diffusion and nuclear dissociation rates.

Related to Figure2: (**A**) Graphs showing the evolution of the p values assessing the statistical differences between the distribution of bleached and reporting EYFP-YAP1 intensities upon nuclear and cytoplasmic FRAP in NF1. FRAP graphs (left) represent median with 95%CI. (**B**) Graph showing the median of EGFP intensities from bleached (plain line) and reporting (dotted line) regions in 5 representative cells upon nuclear FRAP in CAF1. (**C**) Graph showing the median of H2B-EGFP intensities from bleached (plain line) and reporting (dotted line) regions in 5 representative cells upon nuclear FRAP in CAF1. (**D**) Schematic showing effective radius (re), bleach-depth (K) and half-time plots in two contexts: 1. When diffusion is quantifiable or 2. When diffusion is too fast to be estimated. For more details, refer to Mathematical Methods. Mann-Whitney U-test, n.s., non-significant.

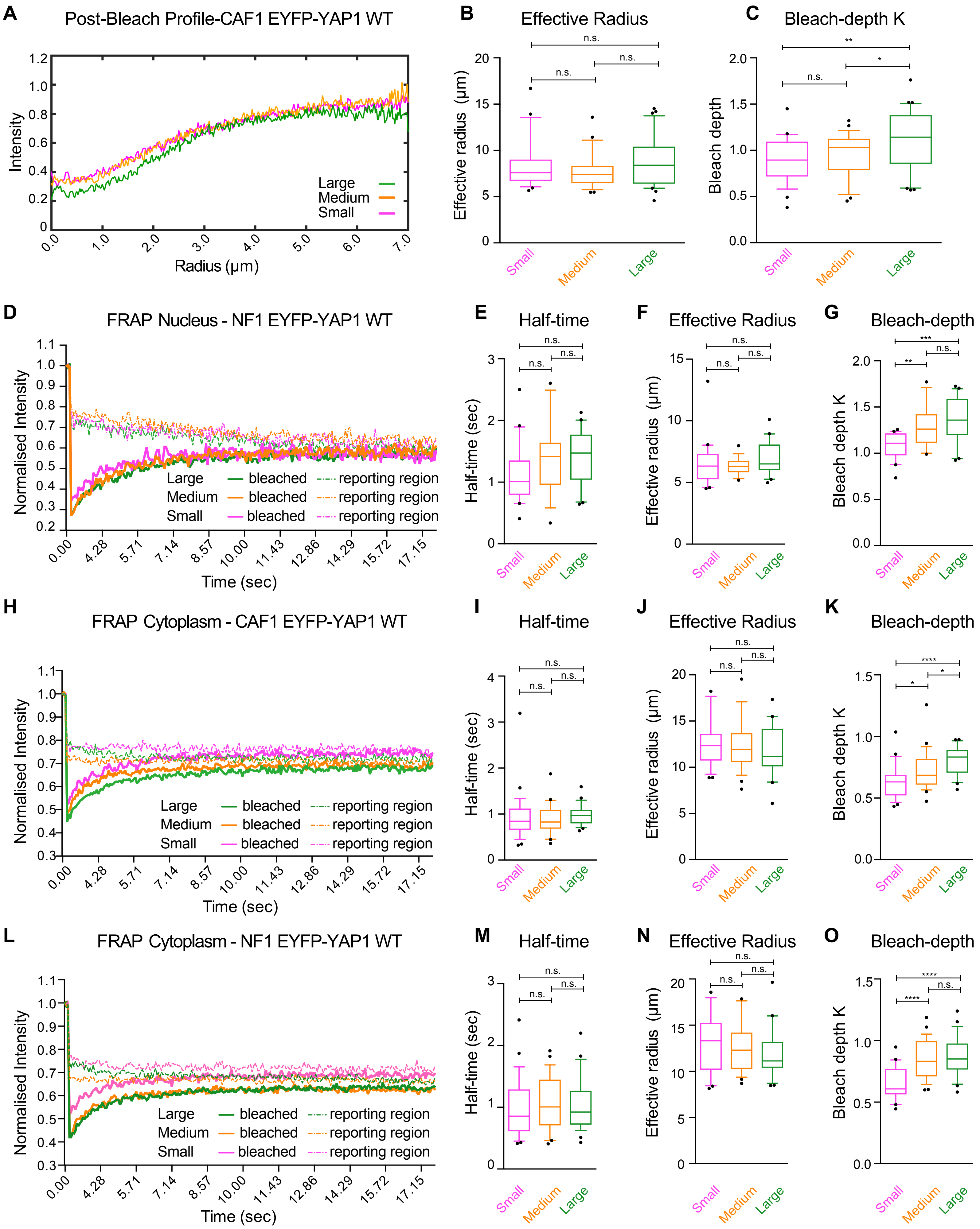

Supplemental Figure S3: Diffusion is fast and does not take part in the recoveries observed upon FRAP. Related to Figure2: Legends next page.

Supplemental Figure S3: Diffusion is fast and does not take part in the recoveries observed upon FRAP. Related to Figure2: (**A**) Post-bleach profiles corresponding to (Figure2B) recoveries of intensities in CAF1 EYFP-YAP1 nuclear FRAP. (B) Box-plot (10&90) showing effective radius corresponding to (Figure2B) recoveries of intensities. (**C**) Box-plot (10&90) showing bleach-depth K corresponding to (Figure2B) recoveries of intensities. (**D**) Graph showing the median intensities of EYFP-YAP1 for three different sized bleached (plain line) and reporting (dotted line) regions upon nuclear FRAP in NF1 EYFP-YAP1. n= 20 cells for each of the sizes from 3 biological replicates. (**E-G**) Box-plot (10&90) showing half-time, effective radius and bleach-depth corresponding to (D) recoveries of intensities. (**H**) Equivalent graph to (D) upon cytoplasmic FRAP in CAF1, n= 30 cells for each small and medium sizes, 25 cells for large size from 3 biological replicates. (**I-K**) Box-plot (10&90) showing half-time, effective radius and bleach-depth corresponding to (H) recoveries of intensities. (**L**) Equivalent graph to (D) upon cytoplasmic FRAP in NF1 EYFP-YAP1. n= 30 cells for each of the sizes and from 3 biological replicates. (**M-O**) Box-plot (10&90) showing half-time, effective radius and bleach-depth corresponding to (L) recoveries of intensities. Mann-Whitney U-test, n.s., non-significant, * $p \leq 0.05$, ** $p \leq 0.01$, *** $p \leq 0.001$, **** $p \leq 0.0001$.

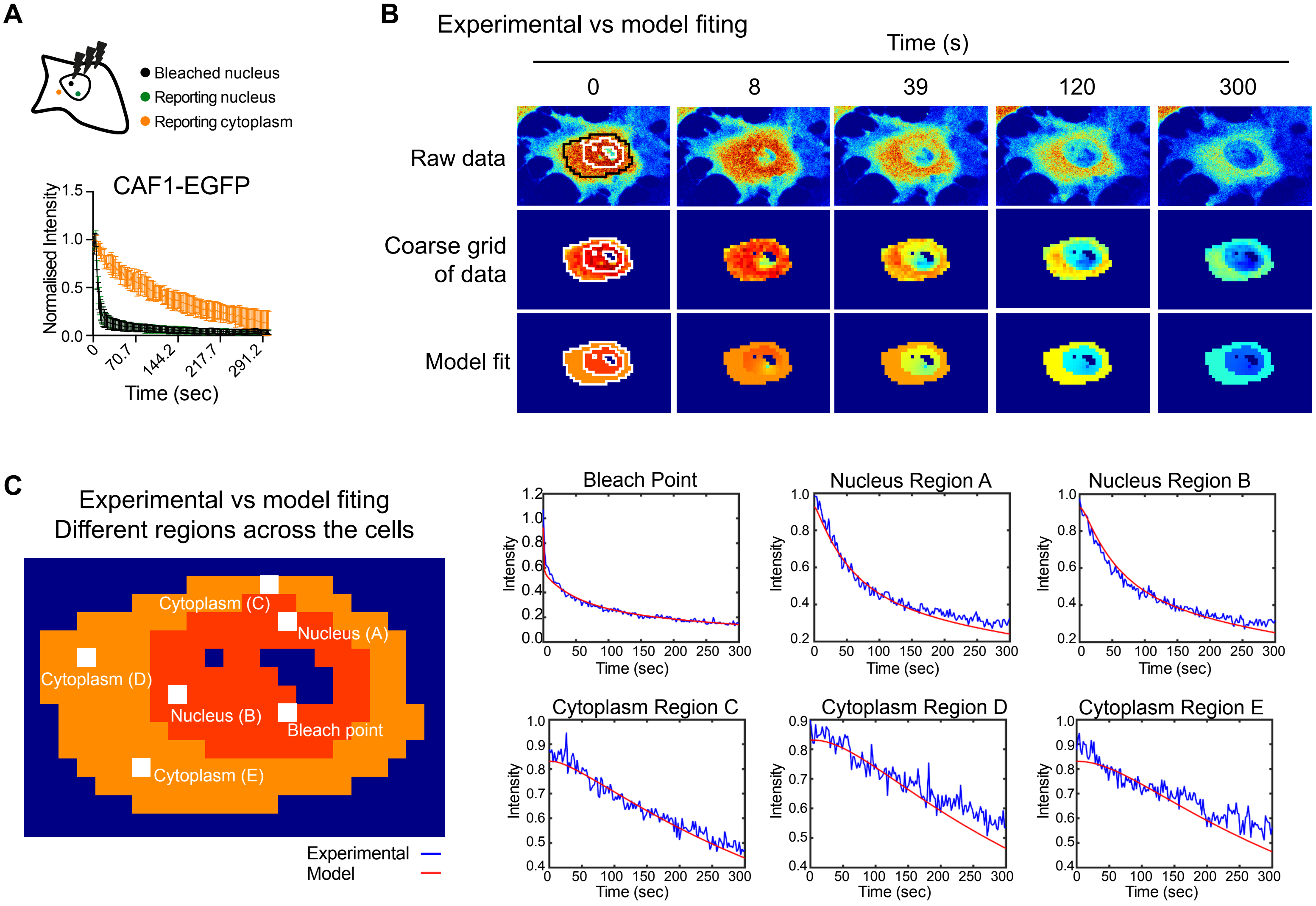

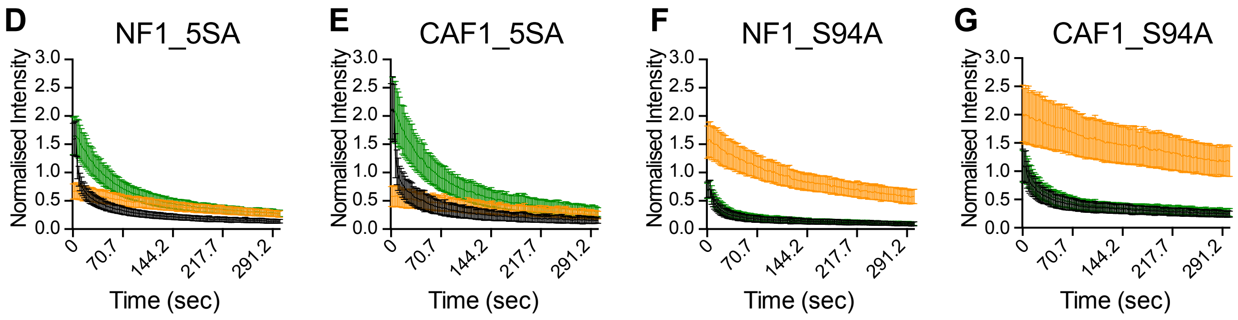

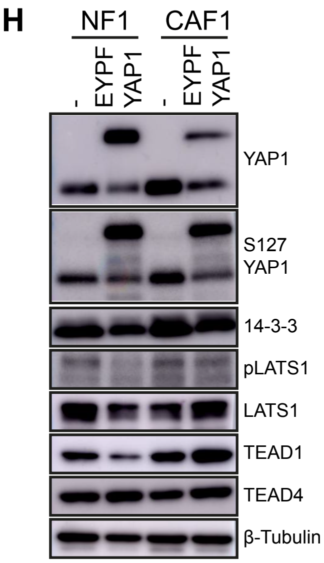

I

YAP1 steady state distribution

	NF1		CAF1	
	**5SA**	**S94A**	**5SA**	**S94A**
**Association (Nucleus)**	0.2078	0.4671	0.1323	0.4819
**Dissociation (Nucleus)**	0.3	0.95	0.2	1.3
**Export**	0.0118	0.0505	0.0127	0.0399
**Import**	0.0175	0.0211	0.0265	0.0155

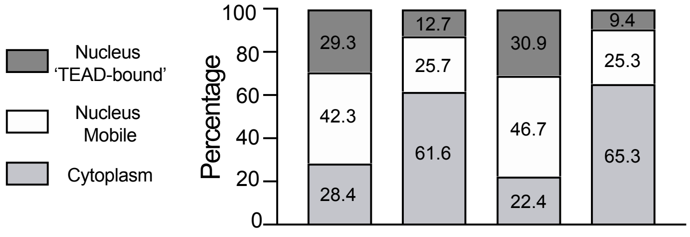

Supplemental Figure S4: Mathematical modeling allows spatial analyses during FLIP experiments. Related to Figure4: Legends next page.

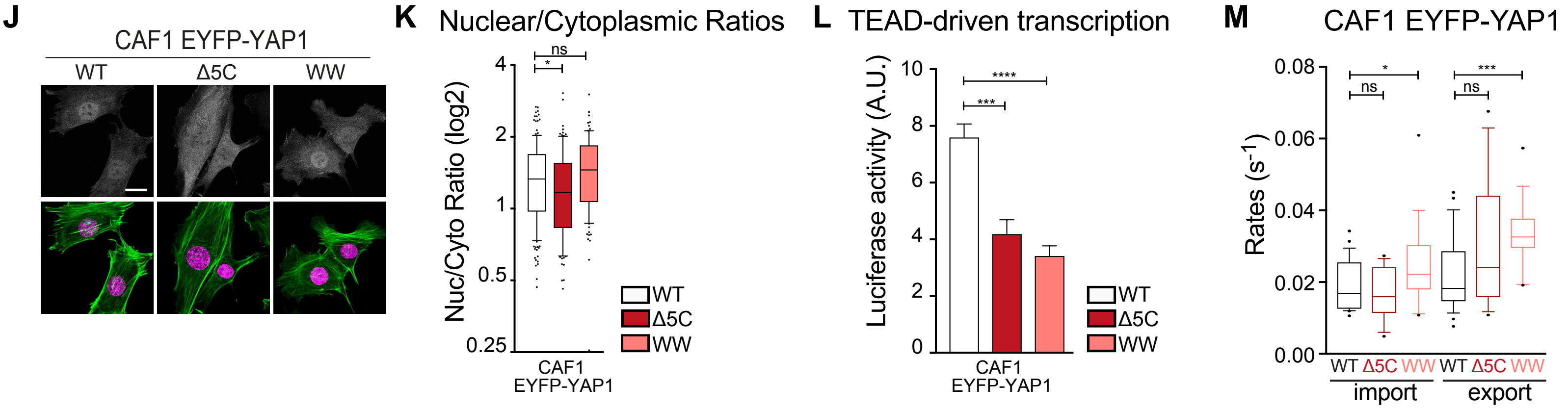

Supplemental Figure S4: Mathematical modeling allows spatial analyses during FLIP experiments.
Related to Figure4: (**A**) Graph showing the evolution of EGFP intensities from bleached (black), nuclear reporting (green) and cytoplasmic reporting (orange) upon nuclear FLIP in 5 representative CAF1 cells. Graph represents mean with 95%CI. (**B**) Comparison of experimental data to FLIP model fitting for a single CAF1 cell undergoing FLIP in the nucleus. The raw experimental data, the coarse-gridded discretization of the experimental data and FLIP PDE model fit to the coarse-gridded discretization are compared for various time-points (0s, 8s, 39s, 120s and 300s). The manually determined boundaries of the cytoplasm, nucleus and nucleoli are illustrated at 0s (see Mathematical Methods). (**C**) Plots of intensity versus time at the bleached point and various labelled points in the nucleus and cytoplasm for the same cell as in (B). Together B and C illustrate both good spatial and temporal fits to the experimental data. (**D**) Equivalent graph to (A) showing the evolution of EYFP-YAP1 5SA intensities upon nuclear FLIP in CAF1, n=26 cells from 3 biological replicates. (**E**) Equivalent graph to (A) showing the evolution of EYFP-YAP1_5SA intensities upon nuclear FLIP in NF1, n=30 cells from 3 biological replicates. (**F**) Equivalent graph to (A) showing the evolution of EYFP-YAP1_S94A intensities upon nuclear FLIP in NF1, n=27 cells from 3 biological replicates. (**G**) Equivalent graph to (A) showing the evolution of EYFP-YAP1_S94A intensities upon nuclear FLIP in CAF1, n=25 cells from 3 biological replicates. (**H**) Western blot of both WT and EYFP-YAP1 expressing NF1 and CAF1. YAP1 (61 and 92kDa), S127 YAP1 (61 and 92kDa), 14-3-3 (27kDa), pLATS1 (140kDa), LATS1 (140kDa), TEAD1 (50kDa), TEAD4 (48kDa), and Beta-Tubulin (42kDa) are represented. (**I**) Mathematical estimation of YAP1 steady-state distribution in NF1 and CAF1 expressing 5SA and S94A mutants. (**J**) Representative images of EYFP-YAP1 localization in CAF1 cell lines expressing EYFP-YAP1_WT vs EYFP-YAP1_ Δ5C or EYFP-YAP1_WW. Scale bar, 20μm. (**K**) Boxplot (10&90) of nuclear-to-cytoplasmic ratio (log2 scale) corresponding to quantification of (J). n>90 cells for each condition from at least 3 independent experiments. (**L**) Luciferase assay of CAF1 cells expressing EYFP-YAP1_WT vs EYFP-YAP1_ Δ5C or EYFP-YAP1_WW. Bars represent mean ± s.e.m. of 3 independent experiments, each with 3 technical replicates. Data are normalised to NF1 EYFP-YAP1_WT. (**M**) Box-plot (10&90) showing different import and export rates in CAF1 EYFP-YAP1_WT vs CAF1 EYFP-YAP1_WW and EYFP-YAP1_ Δ5C cell lines. CAF1 EYFP-YAP1_WT cell line values are reproduced from Figure4C&D. Mann-Whitney U-test, n.s., non-significant, * p≤0.05,¬¬¬ ** p≤0.01, *** p≤0.001, **** p≤0.0001.

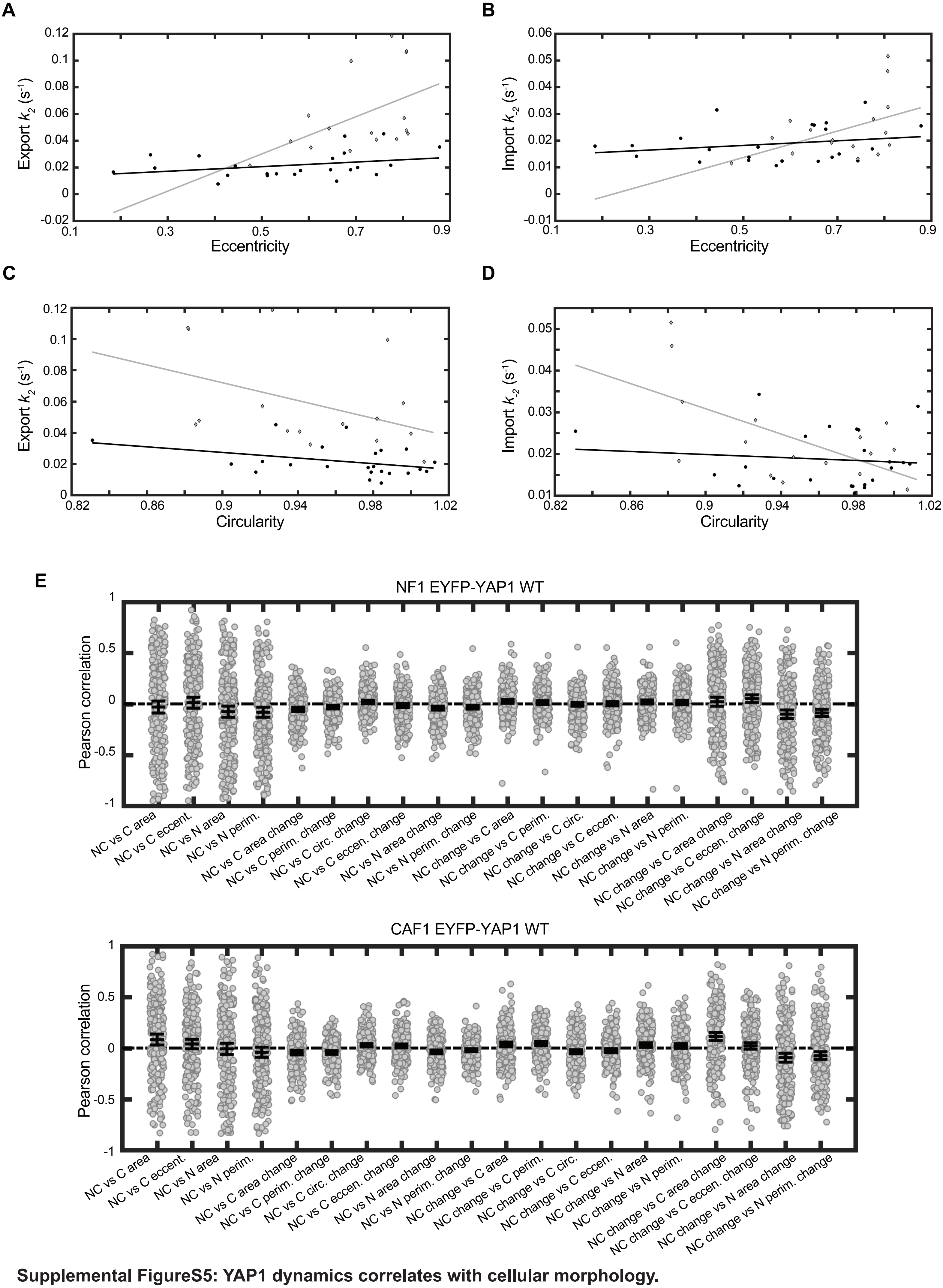

Supplemental FigureS5: YAP1 dynamics correlates with cellular morphology.
Related to Figure5: Legends next page.

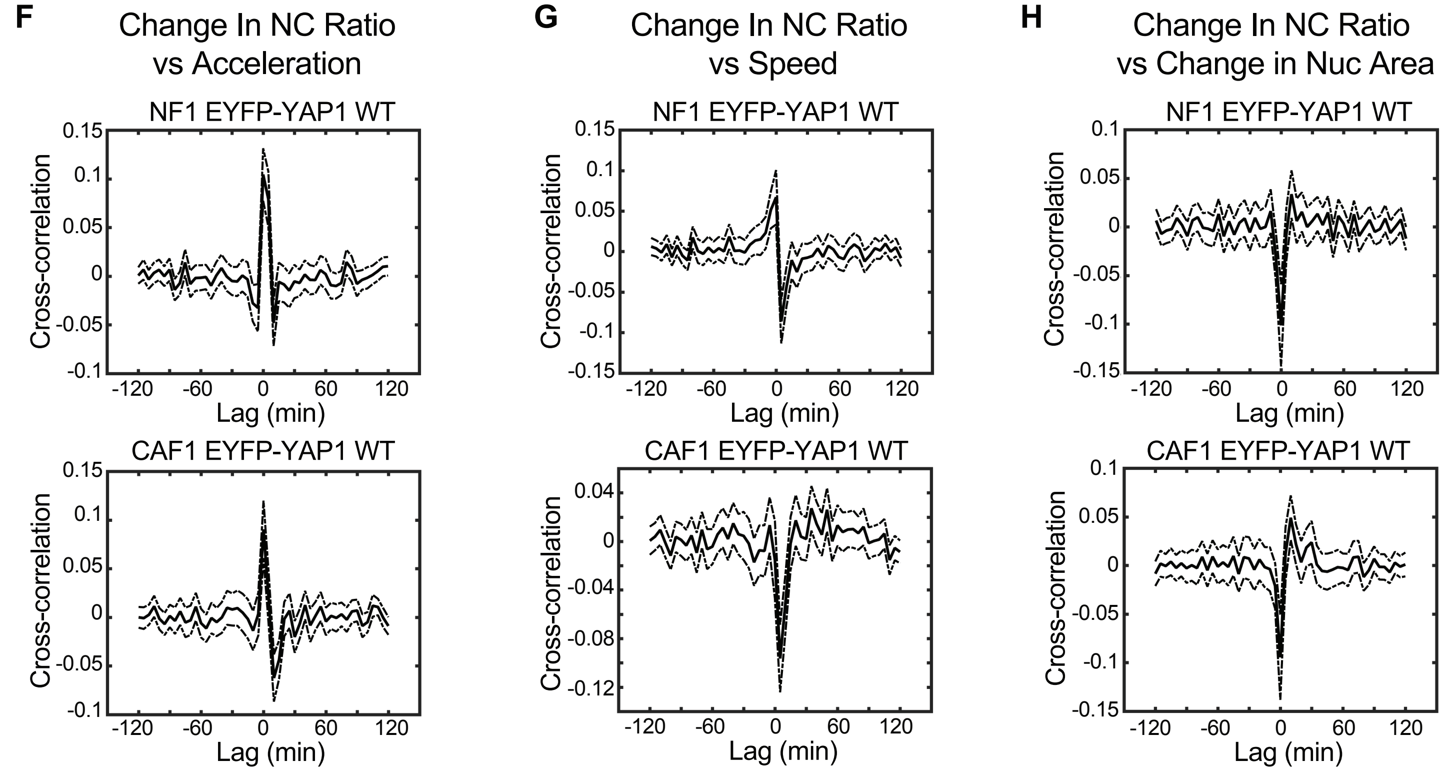

Supplemental FigureS5: YAP1 dynamics correlates with cellular morphology.
Related to Figure5: (**A**) Scatter plot of nuclear eccentricity vs. export and lines of best fit for NF1 EYFP-YAP1_WT (grey) and CAF1 EYFP-YAP1_WT (black). (**B**) Scatter plot of nuclear eccentricity vs. import and lines of best fit for NF1 EYFP-YAP1_WT (grey) and CAF1 EYFP-YAP1_WT (black). (**C**) Scatter plot of nuclear circularity vs. export and lines of best fit for NF1 EYFP-YAP1_WT (grey) and CAF1 EYFP-YAP1_WT (black). (**D**) Scatter plot of nuclear circularity vs. import and lines of best fit for NF1 EYFP-YAP1_WT (grey) and CAF1 EYFP-YAP1_WT (black). (**E**) Scatter plots and 95% CI for additional Pearson correlations of nuclear-to-cytoplasmic ratio, cellular morphology, cell speed and their derivatives for NF1 EYFP-YAP1_WT and CAF1 EYFP-YAP1_WT. (**F-H**) Cross-correlations of change in nuclear-to-cytoplasmic ratio with acceleration (F), speed (G) and change in nuclear area (H) for NF1 EYFP-YAP1_WT and CAF1 EYFP-YAP1_WT. Mean of all cells – solid line, 95% CI – dot-dash line. Mann-Whitney U-test, n.s., non-significant, ** p ≤0.01.

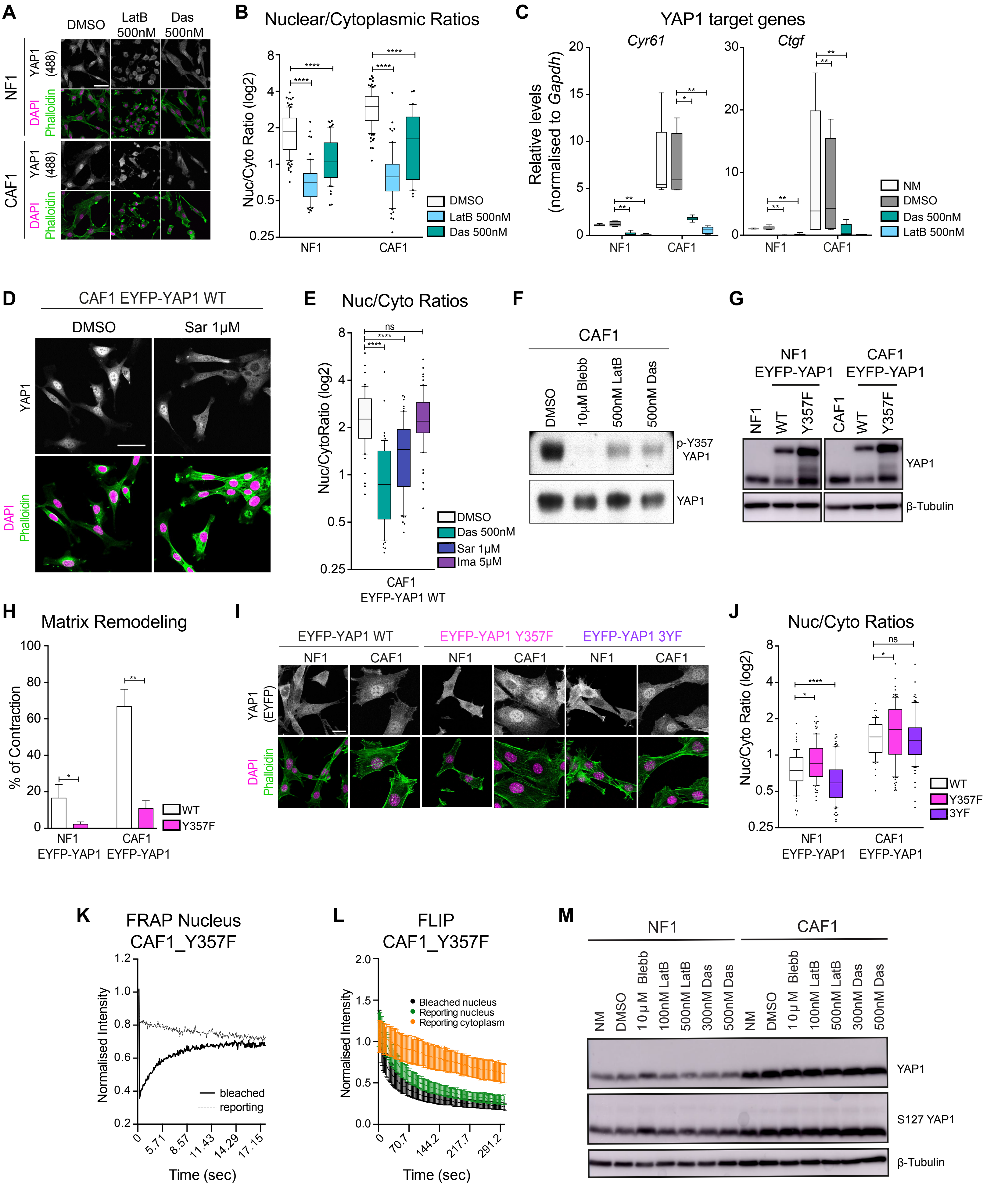

Supplemental Figure S6: Effect of treatment with latrunculin B and dasatinib and of Y357F mutation. Related to Figure6: Legends next page.

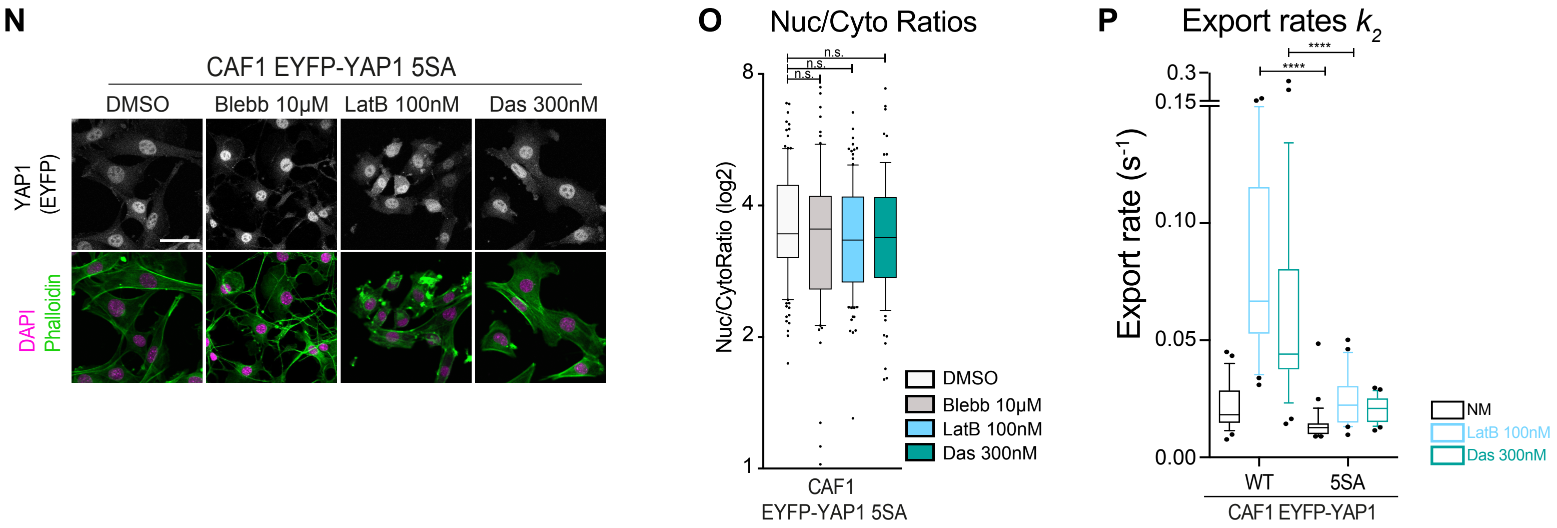

Supplemental Figure S6: Effect of treatment with latrunculin B and dasatinib and of Y357F mutation. Related to Figure6: (**A**) Representative images of endogenous YAP1 localization in NF1 and CAF1 treated with DMSO or 500nM latrunculin B and 500nM dasatinib. Scale bar, 50µm. (**B**) Box-plot (10&90) of nuclear-to-cytoplasmic ratio (log2 scale) of endogenous YAP1 in NF1 and CAF1 treated with DMSO or 500nM latrunculin B and 500nM dasatinib, n>30 cells from at least 2 independent experiments. (**C**) Boxplot (Min to Max) of qRT-PCR of two YAP1 target genes normalised to GAPDH in NF1 and CAF1 cell lines in normal media (NM), or treated with 10µM DMSO, 500nM latrunculin B or 500nM dasatinib. Data summary of 3 independent experiments, each with 2 technical replicates. (**D**) Representative images of EYFP-YAP1 localization in CAF1 after treatment with 1µM saracatinib. Scale bar, 50µm. (**E**) Box-plot (10&90) of nuclear-to-cytoplasmic ratio (log2 scale) of EYFP-YAP1 localization in CAF1 after treatment with DMSO, 500nM dasatinib, 5µM imatinib or 1µM saracatinib. n>60 cells from 2 independent experiments. (**F**) Western blot of YAP1 Immunoprecipitation showing the effects of 10µM blebbistatin, 500nM latrunculin B and 500nM dasatinib treatment on Y357 YAP1 phosphorylation in CAF1 WT. Total YAP1 levels also shown. (**G**) Western Blot showing the expressions of EYFP-YAP1 and EYFP-YAP1_Y357F (92kDa) versus endogenous YAP1 (61kDa), in NF1 and CAF1. Phosphorylation of Serine 127 and Tubulin (42kDa) are also presented. (**H**) Contraction assay of NF1 and CAF1 expressing EYFP-YAP1 vs EYFP-YAP1_Y357F. Bars represent mean ± s.e.m. of 5 independent experiments. (**I**) Representative images of EYFP-YAP1_Y357F and EYFP-YAP1_3YF localization in NF1 and CAF1. Scale bar, 20µm. (**J**) Box-plot (10&90) of nuclear-to-cytoplasmic ratio (log2 scale) of EYFP-YAP1, EYFP-YAP1_Y357F and EYFP-YAP1_3YF localization in NF1 and CAF1, n>100 cells from at least 3 independent experiments. (**K**) Graph showing the median intensities of EYFP-YAP1_Y357F from bleached (plain line) and reporting (dotted line) regions upon nuclear FRAP in CAF1, n= 30 cells from 3 biological replicates. (**L**) Graph showing the intensities of EYFP-YAP1_Y357F from bleached (black), nuclear reporting (green) and cytoplasmic reporting (orange) regions upon nuclear FLIP in CAF1, n= 28 cells from 3 biological replicates. Graph represents mean with 95%CI. (**M**) Western blot showing the effects of 10µM blebbistatin, 100/500nM latrunculin B and 300/500nM dasatinib treatment on S127 YAP1 in NF1 and CAF1 WT. (**N**) Representative images of EYFP-YAP1_5SA in CAF1 treated with DMSO or 10µM blebbistatin, 100nM latrunculin B and 300nM dasatinib. Scale bar, 50µm. (**O**) Box-plot (10&90) of nuclear-to-cytoplasmic ratio (log2 scale) of endogenous EYFP-YAP1_5SA in CAF1 treated with DMSO or 10µM blebbistatin, 100nM latrunculin B and 300nM dasatinib. n>90 cells from 3 independent experimental repeats. (**P**) Box-plot (10&90) showing different export rates in CAF1 EYFP-YAP1_WT and EYFP-YAP1_5SA cell lines upon treatment with 100nM latrunculin B and 300nM dasatinib. Rates of EYFP-YAP1_WT are reproduced from Fig2H for representation Mann-Whitney U-test, n.s., non significant. Mann-Whitney U-test, n.s., non significant, * p ≤0.05, ** p≤0.01, **** p≤0.0001.

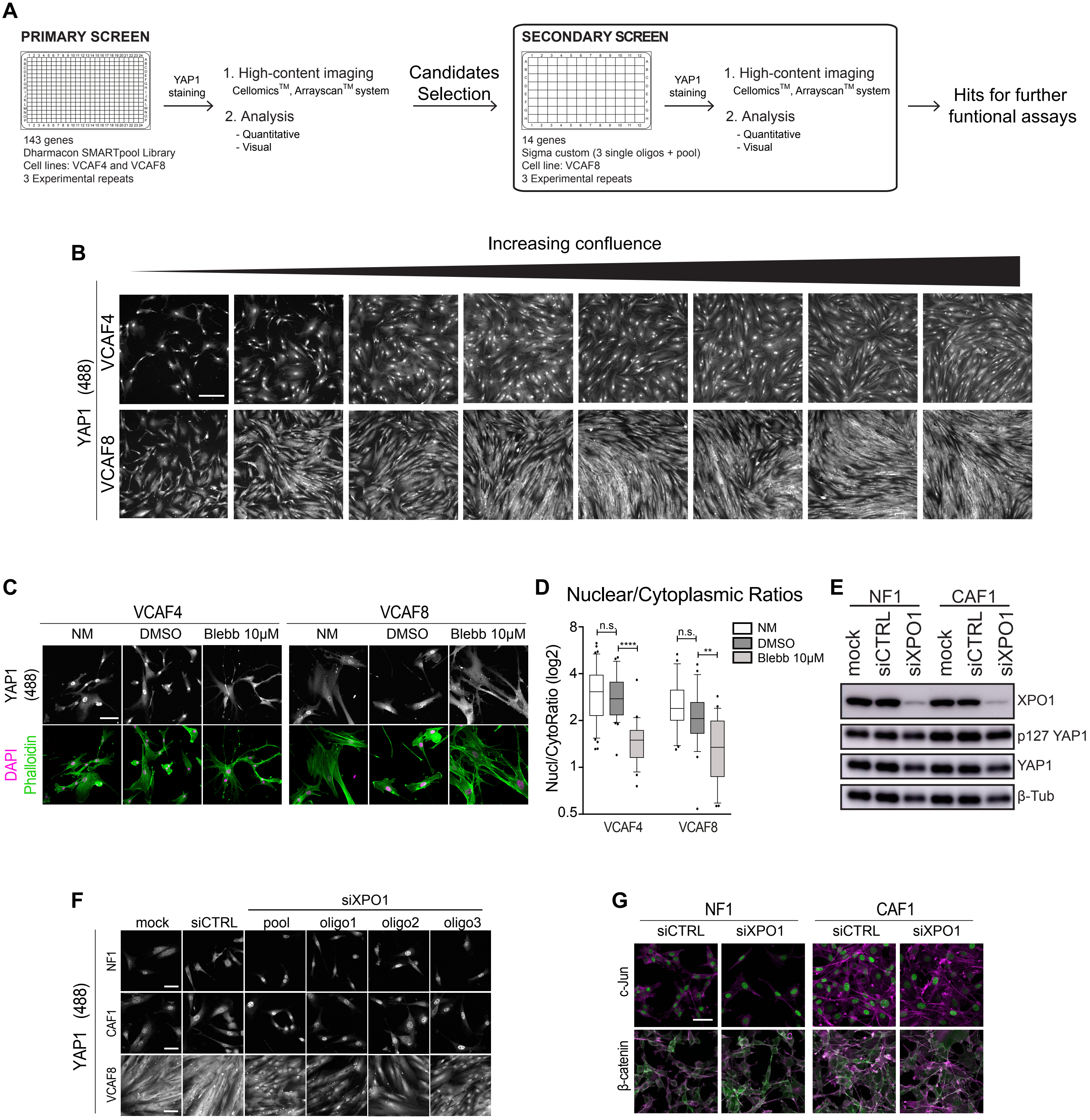

Supplemental Figure S7: Validation of conditions and reagents for the siRNA screen.
Related to Figure7: Legends next page.

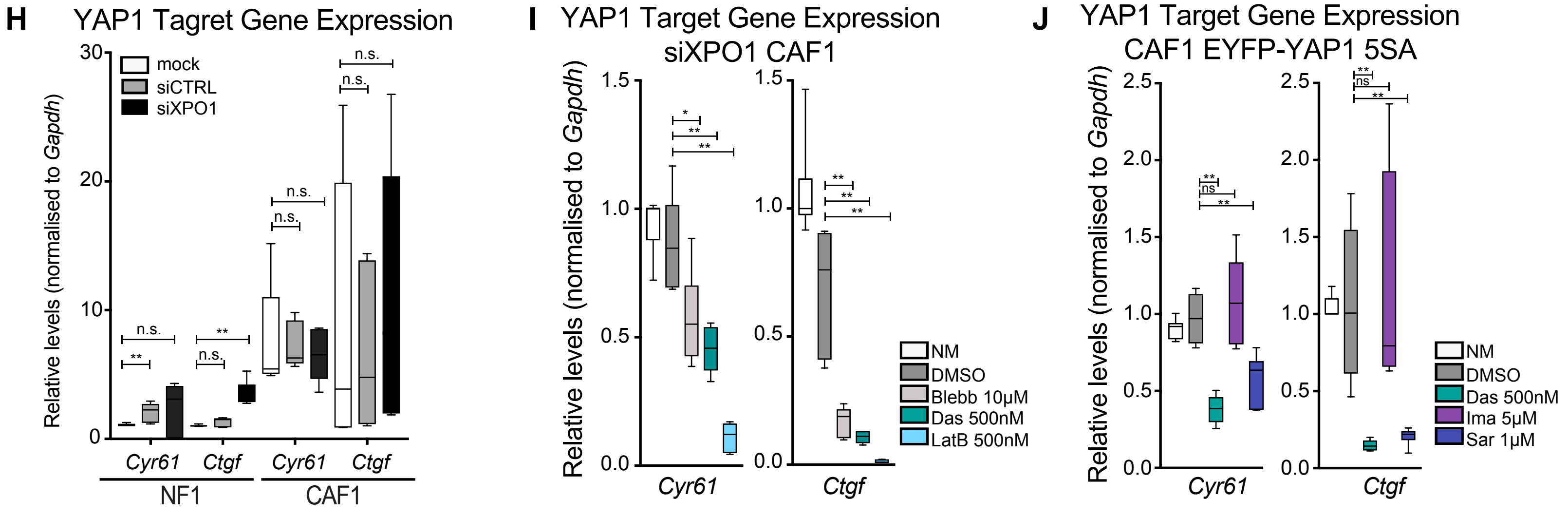

Supplemental Figure S7: Validation of conditions and reagents for the siRNA screen.
Related to Figure7: (**A**) Schematic representation of the screen experimental pipeline. (**B**) Representative images of endogenous YAP1 staining in human VCAF4 and VCAF8 cell lines with increasing cell confluence. Scale bar, 200μm. (**C**) Representative images showing endogenous YAP1 localization in human VCAF4 and VCAF8 cell line treated with 10μM blebbistatin. Scale bar, 100μm. (**D**) Box-plot (10&90) of nuclear-to-cytoplasmic ratio (log2 scale) corresponding to data in n > 21 cells from one experimental repeat. (**E**) Western blot showing depletion of XPO1 (123kDa) in NF1 and CAF1 upon XPO1 depletion. Beta-Tubulin (42kDa) is also presented. (**F**) Representative images showing endogenous YAP1 localization in NF1, CAF1 and VCAF8 upon XPO1 pool deconvolution to single oligos. Scale bar, 50μm. (**G**) Representative images showing endogenous c-Jun or beta-catenin (green) staining upon depletion of XPO1. Actin staining also shown (magenta). Scale bar, 50 μm. (**H**) Boxplot (Min to Max) of qRT-PCR of two YAP1 target genes normalised to GAPDH in NF1 and CAF1 cell lines after depletion of XPO1. Data summary of 3 independent experiments, each with 2 technical replicates. (**I**) Boxplot (Min to Max) of qRT-PCR of two YAP1 target genes normalised to GAPDH in CAF1 cell lines after depletion of XPO1 in normal media (NM), or treated with DMSO, 10μM blebbistatin, 500nM dasatinib or 500nM latrunculin B. Data summary of 3 independent experiments, each with 2 technical replicates. (**J**) Boxplot (Min to Max) of qRT-PCR of two YAP1 target genes normalised to GAPDH in CAF1 EYFP-YAP1_5SA cell line in normal media (NM), or treated with DMSO, 500nM dasatinib, 5μM imatinib or 1μM saracatinib. Data summary of 3 independent experiments, each with 2 technical replicates. Mann-Whitney U-test, n.s., non significant, * p≤0.05, ** p≤0.01, **** p≤0.0001.

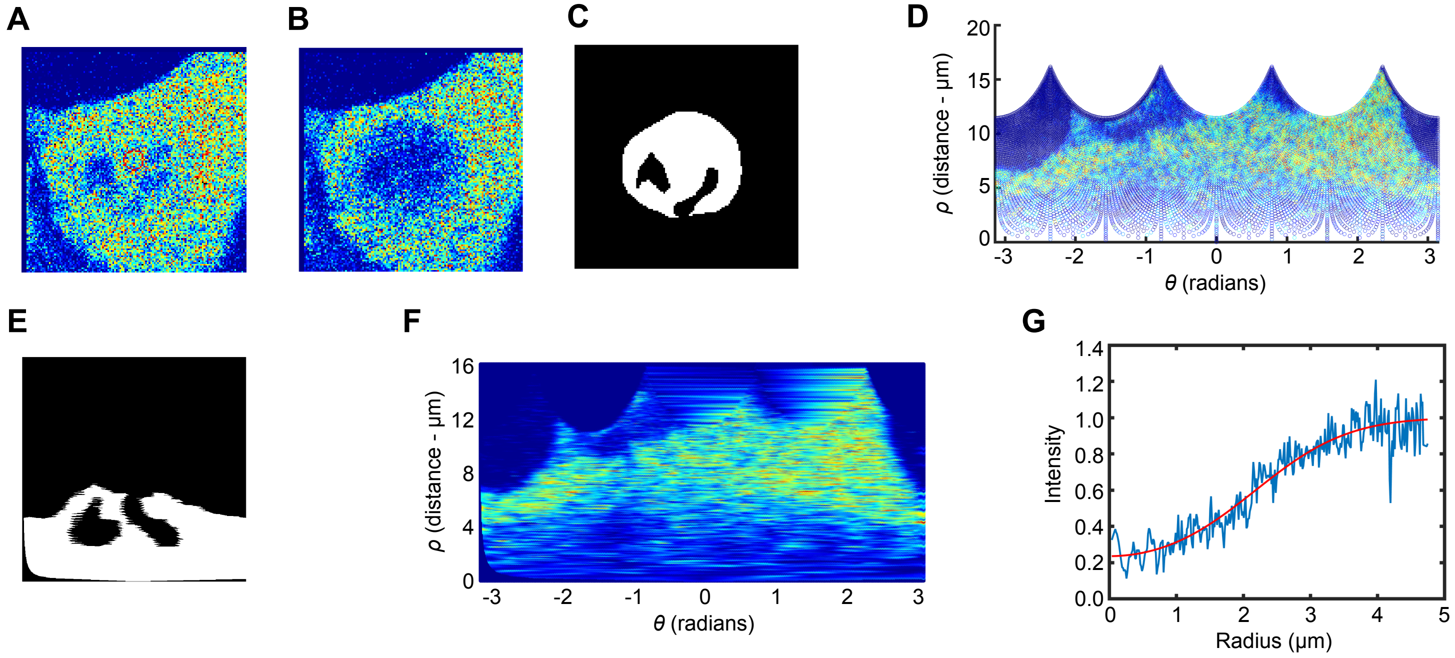

Supplemental Figure S8: FRAP postbleach profile processing and analysis.
Related to STAR Methods - Mathematical Methods: Walkthrough of analysis of the postbleach profile of a single CAF WT cell undergoing FRAP. (**A**) Pre-bleach profile (frame prior to bleach process) re-centred such that the bleach-point (red circle) is in the centre of the image. Nucleoli are observable as regions of low intensity within the nucleus. (**B**) Post-bleach profile (first frame captured upon completion of bleach process) re-centred around bleach-point (red circle in (A)). (**C**) Image mask (re-centred around the bleach-point) outlining the manually determined boundaries of the nucleus and nucle- oli. (**D**) Post-bleach frame in (B) transformed from Cartesian to polar coordinates. (**E**) Image mask in (C) transformed from Cartesian to polar coordinates. (**F**) Result of interpolation of data in (D). (**G**) Model fit of exponen- tial of a Gaussian (Equation (1.1)) (red curve) to median intensity versus distance from bleach-point (in microns) derived from (F) (blue curve).

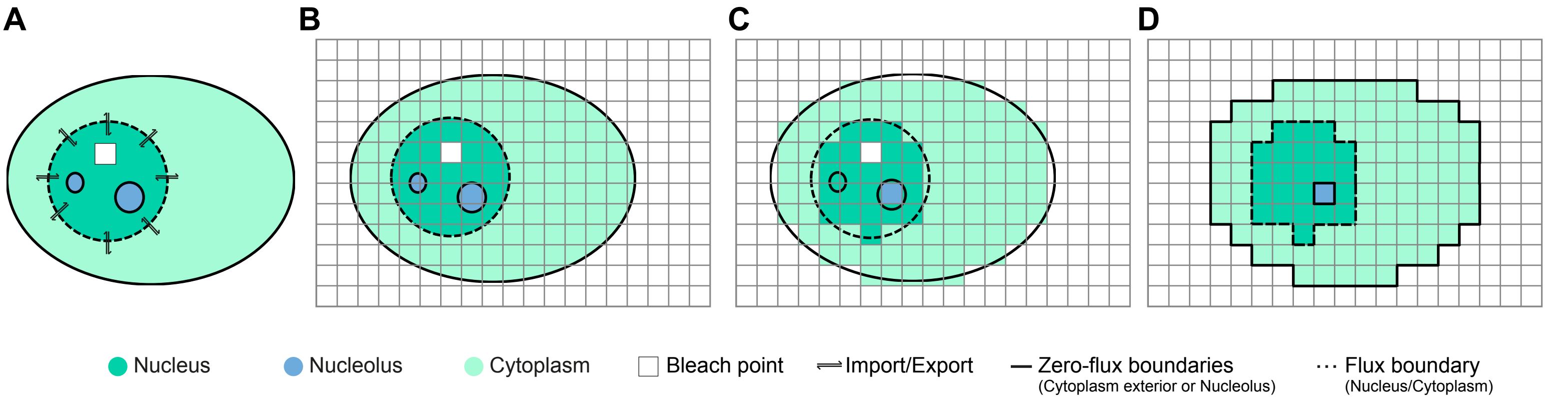

Supplemental Figure S9: FLIP image analysis.
Related to STAR Methods - Mathematical Methods: Walkthrough of PDE model fitting to FLIP imaging data. (**A**) Schematic of Partial Differen- tial Equation (PDE) model approximation to FLIP system. (**B**) Illustration of coarse-grid discretization of cell for numerically solving PDE and fitting to data. (**C**) Lattice-sites in the grid of (B) are determined as nuclear or cytoplasmic if at least 50% of that lattice site is composed of that cellular compartment. (**D**) Final coarse-grid- ded approximation to the original cell. Each grid-point intensity is set at the median intensity of that grid-point. Only pixels from the cellular compartment (nucleus or cytoplasm) that defines the grid-point are used in this calculation. This coarse grid is then used to fit the numerical solution of our PDE to the imaging data.

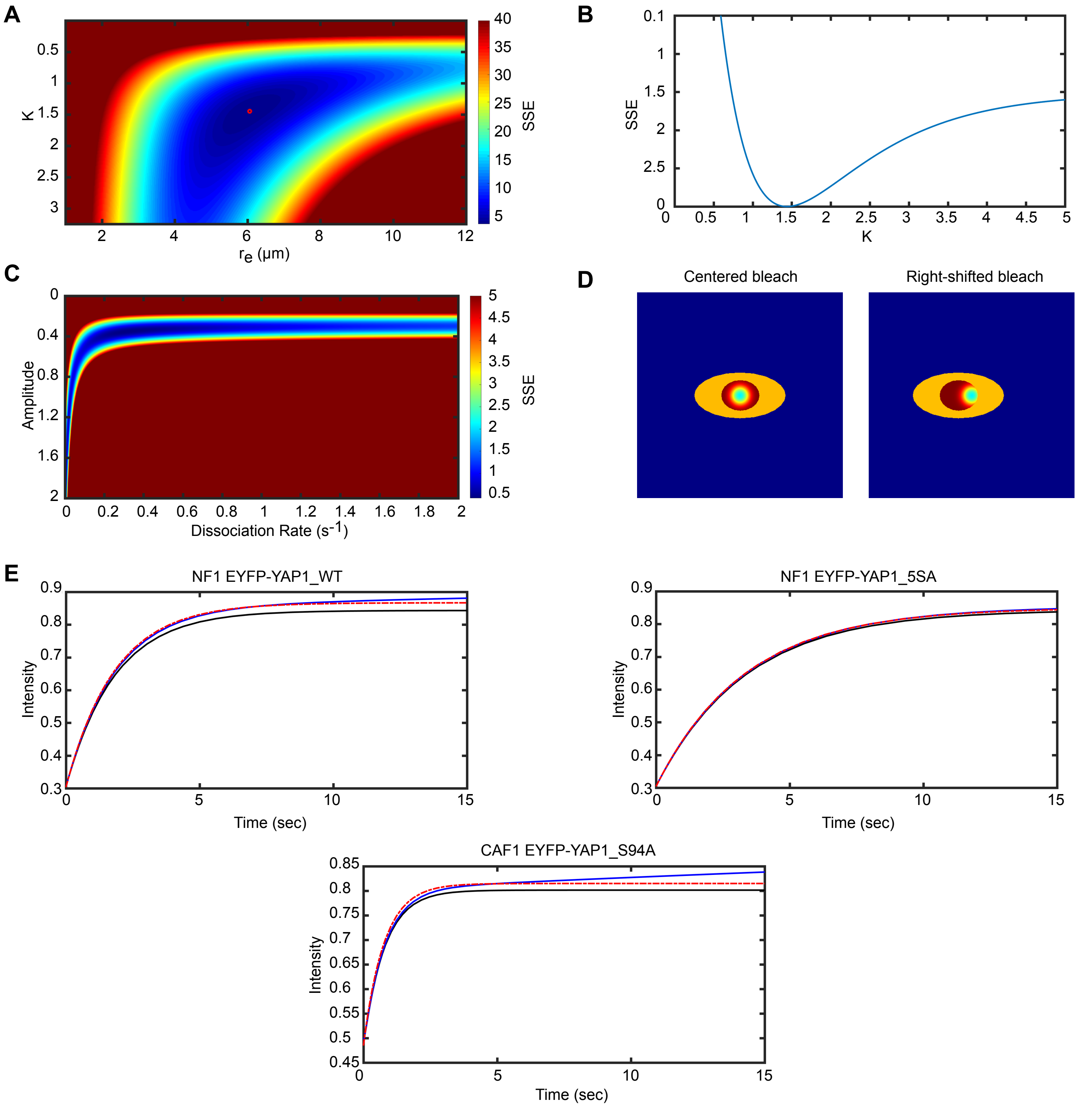

Supplemental Figure S10: FRAP model sensitivity analysis.

Related to STAR Methods - Mathematical Methods: (**A**) Heatmap of Sum of Squares due to Error (SSE) of the exponential of a Gaussian fit (Equation (1.1)) to the median intensity (Supplemental Figure S8G) for varying bleach-depth and effective radius. The red dot signifies the global minimum. (**B**) Plot of SSE as the bleach-depth parameter is varied, when de- riving the bleach-depth parameter from the recovery curve (Equation (1.2)) as opposed to the post-bleach profile. (**C**) Heatmap of SSE of the single reaction model (Equation (1.5)) fit to the recovery curve of a single cell for varying am- plitude and rate of dissociation. (**D**) Initial protein concentrations of the nucleus and cytoplasm (red high, blue low) for sensitivitiy analysis of the assumption of zero import/export for CAF WT with bleach at the centre of the nucleus (left panel) and righthand side of the nucleus (right panel). (**E**) Bleach region recovery comparing the FLIP simulated recovery with non-zero import/export (blue), zero import/export (black) and single reaction FRAP equation (1.5) with dissociation fixed at FRAP quantified levels and other parameters fitted to the recovery with non-zero import/-export (red dot-dash line). The top-left panel recovery is for NF WT with a bleach at the centre of the nucleus, top-right panel for NF 5SA with bleach at the centre of the nucleus and bottom panel is for CAF S94A with bleach on the righthand side of the nucleus.

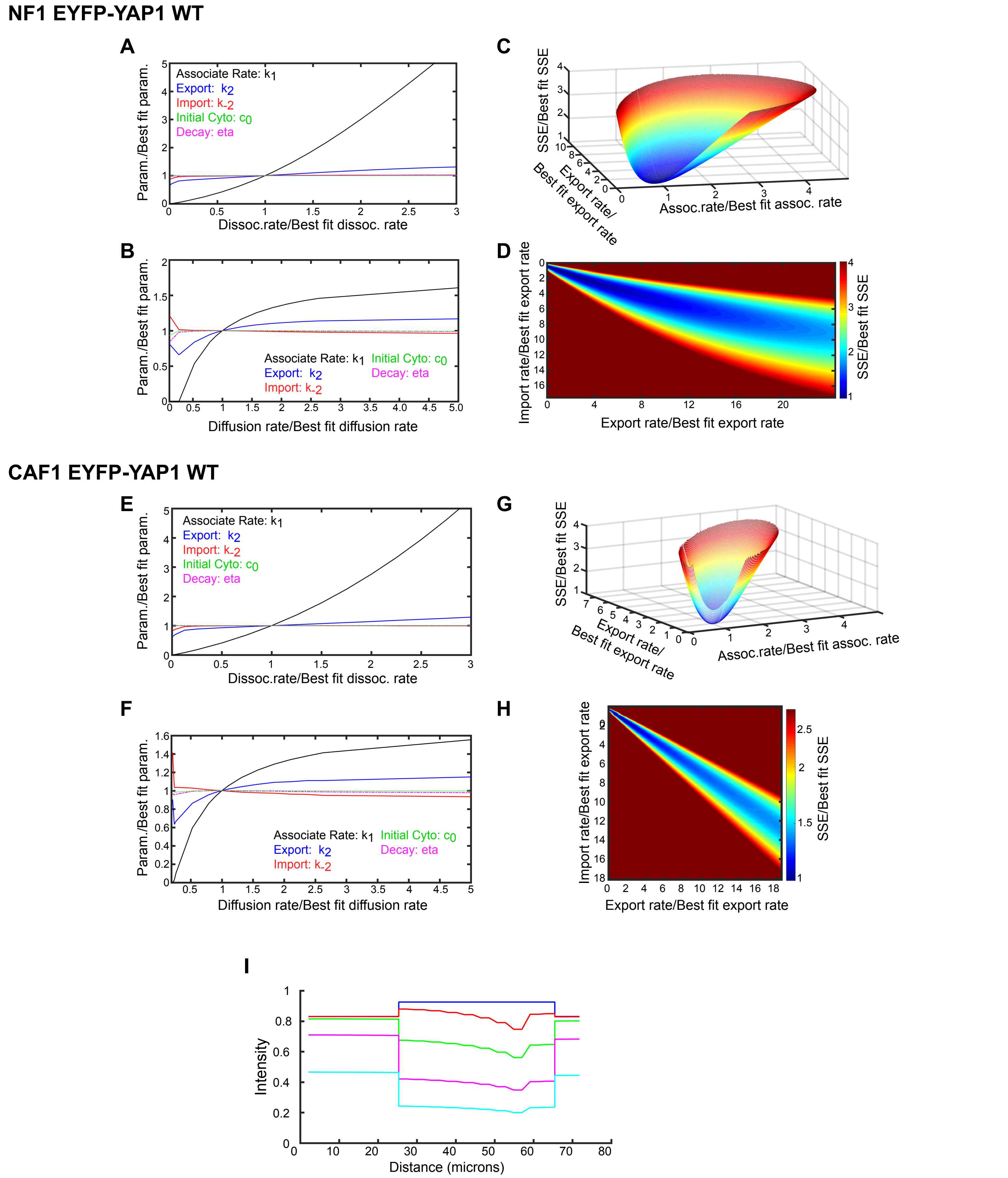

Supplemental Figure S11: FLIP model sensitivity analysis.

Related to STAR Methods - Mathematical Methods: FLIP model sensitivity analysis. Sensitivity analysis of model parameters for a single NF1 EYFP-YAP1 WT cell (Plots A-D) and a single CAF1 EYFP-YAP1 WT cell (E-H). (**A-E**) Plots of sensitivity of association rate, export, import, predicted initial cytoplasmic concentration and decay rate due to bleaching as the fixed dissociation rate is varied. The scales are in terms of ratios of the derived parameter values for a given dissociation rate to the optimum model fit parameter values.

(**B&F**) Plots of sensitivity of association rate, export, import, predicted initial cytoplasmic concentration and decay rate due to bleaching as the fixed rate of diffusion is varied. The scales are in terms of ratios of the derived parameter values for a given rate of diffusion to the optimum model fit parameter values. (**C&G**) Surface plots of the weighted-SSE (Equation (1.29)) for varying association rate and export. Scales are presented in terms of ratio of parameter values to optimum fit parameter values and ratio of weighted-SSE for those parameter values to the weighted-SSE for optimum parameter values. (**D&H**) Heatmaps of the weighted-SSE and the overall best-fit weighted-SSE for varying import and export. Scales are again in terms of ratios of specific parameter values to optimum fit parameter values. (**I**) Horizontal linescan of complete cell for FLIP mathematical solution in Figure S4B at timepoints 0s (blue), 8s (red), 39s (green), 120s (magenta) and 300s (cyan). The discontinuous jumps in intensity illustrate the boundary of the cytoplasm and nucleus.

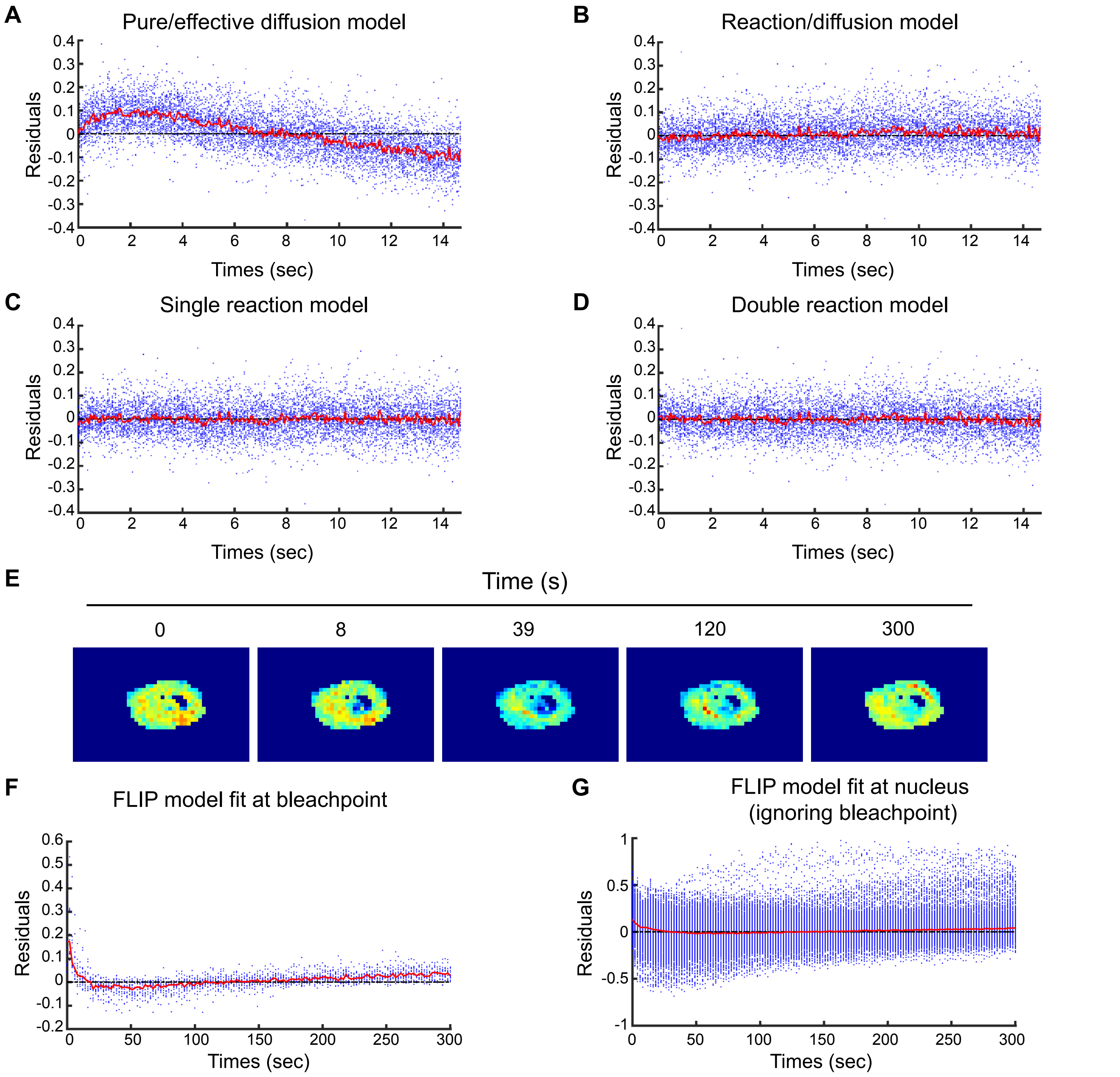

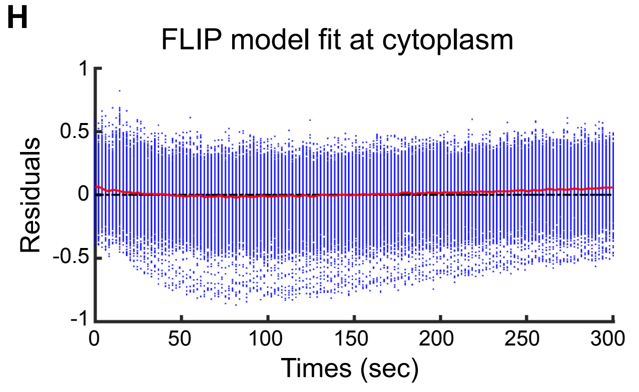

Supplemental Figure S12: FRAP and FLIP residual analysis. Related to STAR Methods - Mathematical Methods: Residuals (observed minus predicted values) versus time for FRAP recovery curve model fitting of 30 NF1 EYFP-YAP1 WT cells (ten cells with each of small, medium and large nominal bleach regions) for: (**A**) pure/effective diffusion model, (**B**) reaction-diffusion model, (**C**) single reaction model and (**D**) double reaction model. Figures E-H constitute a residual analysis of FLIP model fitting to CAF1 EYFP-YAP1 WT cells. (**E**) Spatial residuals at timepoints 0s, 8s, 39s, 120s and 300s for FLIP model-fitting of a CAF WT cell (see Supplementary Figure S4B). (**F**) Residuals versus time of FLIP model fit at the nuclear bleachpoint of 15 CAF WT cells. (**G**) Residuals versus time of FLIP model fit at all nuclear lattice- sites (ignoring bleach-points) for 15 CAF WT cells. (**H**) Residuals versus time of FLIP model fit at all cytoplasmic lattice-sites of 15 CAF WT cells.

Table S1: Table compiling output of mathematical models fitting experimental data for nuclear FRAP of NF1 and CAF1 expressing EYFP-YAP1. Related to Figure 2.

See excel file called Table S1.

Table S2: Result of the primary siRNA screen in human CAFs. Related to Figure 6.

Targets		VCAF8			VCAF4		Selected for secondary screen
		Repeat1	Repeat2	Repeat3	Repeat1	Repeat2	
Risk-Free	rf	ND	ND	ND	ND	ND	
	rf	ND	ND	ND	ND	ND	
	rf	ND	ND	ND	ND	ND	
	rf	ND	ND	ND	ND	ND	
	rf	ND	MC	MC	ND	ND	
	rf	ND	ND	ND	ND	ND	
	rf	ND	ND	ND	MC	ND	
	rf	ND	ND	ND	ND	ND	
Controls targets	ubb	N/A	N/A	N/A	N/A	N/A	
	ubb	N/A	N/A	N/A	N/A	N/A	
	ubb	N/A	N/A	N/A	N/A	N/A	
	ubb	N/A	N/A	N/A	N/A	N/A	
	yap	N/A	N/A	N/A	N/A	N/A	
	yap	N/A	N/A	N/A	N/A	N/A	
	yap	N/A	N/A	N/A	N/A	N/A	
	yap	N/A	N/A	N/A	N/A	N/A	
	yap	N/A	N/A	N/A	N/A	N/A	
	yap	N/A	N/A	N/A	N/A	N/A	
	yap	N/A	N/A	N/A	N/A	N/A	
	yap	N/A	N/A	N/A	N/A	N/A	
	lat	MN	VN	VN	ND	MN	
	lat	MN	VN	VN	ND	MN	
	lat	MN	VN	VN	ND	MN	
	lat	MN	VN	VN	ND	MN	
	lat	VN	VN	VN	ND	MN	
	lat	VN	VN	VN	ND	MN	
	taz	ND	ND	ND	ND	ND	
	taz	ND	ND	ND	ND	MC	
	taz	ND	ND	ND	ND	ND	
	taz	ND	ND	ND	ND	ND	

Targets		VCAF8			VCAF4		Selected for secondary screen
		Repeat1	Repeat2	Repeat3	Repeat1	Repeat2	
No treatment	empty well	ND	ND	ND	ND	ND	
	empty well	ND	ND	ND	ND	ND	
	empty well	ND	ND	ND	ND	ND	
	empty well	ND	ND	ND	ND	ND	
	empty well	ND	ND	ND	ND	ND	
	empty well	ND	ND	ND	ND	ND	
	empty well	ND	ND	ND	ND	ND	
	empty well	ND	ND	ND	ND	ND	
	empty well	ND	ND	ND	ND	ND	
	empty well	ND	ND	ND	ND	ND	
	empty well	ND	ND	ND	ND	ND	
	empty well	MN	ND	ND	ND	MN	
	empty well	ND	ND	ND	MC	MC	
	empty well	ND	ND	ND	ND	MC	
	empty well	ND	ND	ND	ND	ND	
	empty well	ND	ND	ND	ND	MC	
	empty well	ND	ND	ND	ND	ND	
	empty well	ND	ND	ND	ND	ND	
	empty well	ND	ND	ND	ND	ND	
	empty well	ND	ND	ND	ND	ND	
	empty well	ND	ND	ND	ND	ND	
	empty well	ND	ND	ND	ND	ND	
	empty well	ND	ND	ND	ND	MC	
	empty well	ND	ND	ND	ND	ND	
	empty well	ND	ND	ND	ND	ND	
	empty well	ND	ND	ND	ND	ND	
	empty well	ND	ND	ND	ND	ND	
	empty well	ND	ND	ND	ND	ND	
	empty well	ND	ND	ND	ND	ND	
	empty well	ND	ND	ND	ND	ND	
	empty well	ND	ND	ND	ND	ND	
	empty well	ND	ND	ND	ND	ND	
	empty well	ND	ND	ND	ND	ND	
	empty well	ND	ND	ND	ND	ND	
	empty well	ND	ND	ND	ND	ND	
	empty well	ND	ND	ND	ND	ND	
	empty well	ND	ND	ND	ND	ND	
	empty well	ND	ND	ND	ND	ND	
	empty well	ND	ND	ND	ND	ND	
	empty well	ND	ND	ND	ND	ND	
	empty well	ND	ND	ND	ND	ND	
	empty well	ND	ND	ND	ND	ND	
	empty well	ND	ND	ND	ND	ND	
	empty well	ND	ND	ND	ND	ND	
	empty well	ND	ND	ND	ND	ND	
	empty well	ND	ND	ND	ND	ND	
	empty well	ND	ND	ND	ND	ND	
	empty well	ND	ND	ND	ND	MN	
	empty well	ND	ND	ND	ND	ND	
	empty well	ND	ND	ND	ND	ND	
	empty well	ND	ND	ND	ND	ND	
	empty well	ND	ND	ND	ND	ND	
	empty well	ND	ND	ND	ND	ND	
	empty well	ND	ND	ND	ND	ND	
	empty well	ND	ND	ND	ND	MC	
	empty well	ND	ND	ND	ND	ND	
	empty well	ND	ND	ND	ND	ND	
	empty well	ND	ND	ND	ND	ND	
	empty well	ND	ND	ND	ND	ND	
	empty well	ND	ND	ND	ND	ND	
	empty well	ND	ND	ND	ND	ND	
	empty well	ND	ND	ND	ND	ND	
	empty well	ND	ND	ND	ND	ND	
	empty well	ND	ND	ND	ND	ND	
	empty well	ND	ND	ND	ND	ND	
	empty well	ND	ND	ND	ND	ND	
	empty well	ND	ND	ND	ND	ND	
	empty well	ND	ND	ND	ND	ND	
	empty well	ND	ND	ND	ND	ND	
	empty well	MC	ND	ND	ND	ND	

Targets		VCAF8			VCAF4		Selected for secondary screen
		Repeat1	Repeat2	Repeat3	Repeat1	Repeat2	
Targets	AKAP6	MN	ND	MN	ND	ND	
	LOC340312	ND	ND	ND	ND	ND	
	AKAP8	MN	ND	ND	ND	ND	
	LOC401391	ND	MC	ND	MC	ND	
	ANXA11	MN	ND	MN	ND	ND	
	LOC402569	ND	ND	ND	MC	ND	
	BDP1	ND	ND	ND	ND	ND	
	LOC402715	MN	ND	ND	ND	ND	
	C14ORF1	MN	ND	MN	ND	ND	
	PTTG1IP	MN	MN	MN	ND	ND	
	C20ORF77	ND	ND	MN	ND	ND	
	CHC1	MN	MN	ND	ND	ND	
	C9ORF126	MN	ND	MN	ND	ND	
	CSE1L	MN	MN	MN	ND	ND	
	C9ORF67	MN	ND	MN	ND	ND	
	GLE1L	ND	MN	ND	ND	ND	
	CGI-49	ND	ND	ND	ND	ND	
	**HRB**	**MN**	**VN**	**MN**	**MN**	**MN**	2nd screen
	DKFZP434F2021	MN	VN	MN	MN	MN	
	KPNA1	ND	ND	ND	ND	MC	
	DKFZP564C186	ND	ND	ND	ND	ND	
	KPNB1	ND	MC	MC	ND	MC	
	DKFZP586B1621	MN	MN	MN	ND	MN	
	KPNA2	MN	ND	MN	MC	ND	
	DKFZP586J0619	MN	ND	ND	MC	ND	
	KPNA3	ND	ND	ND	ND	ND	
	DULLARD	ND	ND	ND	ND	ND	
	KPNA4	ND	ND	ND	ND	ND	
	EMD	ND	ND	ND	ND	ND	
	KPNA5	MN	MN	MN	ND	ND	
	FAM34A	ND	ND	ND	ND	ND	
	TNPO1	ND	MC	ND	MC	MC	
	**FLJ10330**	**MN**	**MN**	**MN**	**MN**	**MN**	2nd screen
	KPNB3	ND	ND	ND	ND	ND	
	FLJ10407	ND	ND	ND	ND	ND	
	NUP88	MN	MN	ND	ND	ND	
	FLJ10637	ND	ND	ND	ND	ND	
	NUP98	MN	MN	ND	ND	MN	
	FLJ10774	MN	ND	ND	MN	MN	
	RAN	ND	MN	ND	MC	MC	
	FLJ11127	ND	MN	MN	ND	ND	
	RANBP1	ND	ND	ND	ND	ND	
	FLJ12519	ND	ND	ND	ND	ND	
	RANBP2	ND	ND	ND	MC	ND	
	FLJ14803	ND	ND	ND	ND	ND	
	**RANGAP1**	**MN**	**MN**	**MN**	**MN**	**VN**	2nd screen
	FLJ20273	MN	ND	ND	MC	ND	
	SEC13L1	MN	MN	MN	ND	MN	
	FLJ20297	MN	MN	MN	ND	ND	
	TPR	MN	MN	MN	ND	ND	
	FLJ22353	MN	MN	MN	ND	MN	
	**XPO1**	**ND**	**ND**	**ND**	**MN**	**VN**	2nd screen
	FLJ23323	MN	MN	MN	ND	ND	
	**ZFP36**	**MN**	**MN**	**MN**	**VN**	**MN**	2nd screen
	FLJ30668	ND	ND	ND	ND	ND	
	**NUP214**	**MN**	**MN**	**MN**	**VN**	**MN**	2nd screen
	FLJ39369	MN	MN	MN	ND	ND	
	AAAS	MN	MN	MN	ND	ND	
	GNAZ	ND	ND	ND	MC	ND	
	RAE1	MN	MN	ND	ND	ND	
	HAX1	ND	ND	ND	MC	MC	
	**RANBP3**	**MN**	**MN**	**VN**	**MN**	**MN**	2nd screen
	JMJD1B	MN	MN	MN	MC	MC	
	NUP155	ND	MN	MN	MN	MN	
	KIAA0007	ND	ND	ND	ND	MC	
	IPO13	ND	ND	ND	ND	MC	
	KIAA0133	MN	MN	MN	ND	ND	
	NUP93	ND	ND	ND	ND	MN	
	KIAA1161	ND	ND	ND	MN	ND	
	NUPL1	ND	MN	ND	ND	ND	
	LAP1B	MN	ND	ND	ND	ND	
	POM121	ND	ND	ND	ND	MC	
	LBR	ND	MC	VC	ND	ND	
	NUP153	MN	MN	MN	ND	ND	
	LEMD2	MN	ND	ND	ND	ND	
	RANBP9	VC	MC	VC	VC	ND	
	LOC163590	ND	MN	ND	ND	MN	
	NUTF2	MN	ND	ND	MN	MN	
	LOC375616	MN	MN	MN	MN	MN	
	NXF1	VC	MC	MC	VC	VC	
	MAN1	MN	ND	VN	MN	MN	
	IPO8	ND	MC	ND	MC	ND	
	MGC3162	MN	ND	ND	ND	MC	
	IPO7	MN	MN	ND	ND	ND	
	MGC33329	MN	ND	ND	ND	ND	
	NUP50	MN	MN	MN	ND	ND	
	MGC45780	MN	MN	MN	MN	MN	
	NUPL2	ND	ND	ND	MN	ND	
	NRM	VC	MC	VC	MC	MC	
	XPOT	MN	ND	MN	MN	ND	
	P15RS	ND	ND	ND	ND	ND	
	DDX19	ND	ND	MN	ND	ND	
	PSFL	ND	ND	ND	ND	ND	
	CASC3	ND	ND	ND	ND	MC	
	PTGDS	ND	ND	ND	ND	MC	
	XPO7	VC	VC	VC	ND	MC	
	RTN4	ND	ND	ND	ND	MC	
	NUP205	MC	MC	VC	MC	MC	
	S100A6	ND	ND	ND	MN	ND	
	XPO6	ND	ND	ND	ND	ND	
	SLC25A22	ND	MN	MN	ND	ND	
	NUP210	ND	ND	ND	ND	ND	
	SLC39A14	ND	ND	MN	ND	MN	
	NUP160	ND	ND	ND	ND	ND	
	SP140	MC	MC	MC	MC	MC	
	**NUP188**	**MN**	**MN**	**MN**	**ND**	**ND**	2nd screen
	SYNE1	ND	ND	ND	ND	ND	
	TNPO3	MN	ND	MN	ND	MN	
	SYNE2	MN	MN	MN	ND	ND	
	KPNA6	ND	ND	ND	ND	ND	
	TMPO	ND	ND	ND	ND	ND	
	NUP62	MC	MC	MC	MC	MC	
	TREX1	ND	ND	ND	ND	MC	
	POM121L1	MN	VN	MN	MN	MN	
	UNC84B	ND	ND	MC	ND	MC	
	**RANBP6**	**VN**	**MN**	**MN**	**MN**	**MN**	2nd screen
	WDR33	ND	ND	ND	ND	ND	
	RANGNRF	ND	ND	ND	ND	ND	
	UNC84A	ND	ND	ND	MC	MC	
	NXT1	ND	ND	ND	ND	MC	
	RP13-15M17.2	ND	ND	ND	ND	ND	
	TNPO2	ND	ND	ND	MC	MC	
	IPO11	ND	ND	ND	ND	MC	
	NUP54	ND	ND	ND	ND	ND	
	IPO9	MN	ND	MN	ND	ND	
	NUP133	MC	VC	MC	ND	MC	
	**NXF5**	**VN**	**MN**	**VN**	**MN**	**MN**	2nd screen
	**NXF3**	**VN**	**MN**	**VN**	**MN**	**VN**	2nd screen
	NXF2	ND	ND	ND	ND	ND	
	EIF4ENIF1	MN	MN	ND	MN	MN	
	NUP107	MN	MN	MN	ND	ND	
	XPO5	ND	ND	ND	ND	ND	
	RANBP10	MN	MN	MN	ND	ND	
	XPO4	ND	ND	ND	MC	ND	
	**RANBP17**	**VN**	**MN**	**MN**	**MN**	**MN**	2nd screen
	NUP37	ND	ND	ND	ND	ND	
	IPO4	MN	MN	MN	MC	MC	
	PCNT1	MN	MN	MN	MN	ND	
	SEC13L	ND	ND	ND	MN	ND	
	**RANBP2L1**	**MN**	**MN**	**ND**	**ND**	**ND**	2nd screen
	**THOC3**	**MN**	**MN**	**MN**	**MN**	**MN**	2nd screen
	LOC129401	MC	MC	MC	ND	ND	
	NUP43	MN	MN	ND	MC	MC	

Table S3: siRNA oligonucleotide sequences. Related to STAR Methods.

siRNAs	company	Cat. No.	Sequence	Species
CTRL Allstars Negative Control siRI	Qiagen	1027280	Proprietary	human, mou
YAP1 oligo1	Dharmacon	D-046247-01	ACAGGUGGCUCAAUUCUUG	mouse
YAP1 oligo4	Dharmacon	D-046247-04	GCCGAGAAGUGCAGUCCAA	mouse
YAP1 oligo1	Dharmacon	D-012200-01	GGUCAGAGAUACUUCUUA	human
YAP1 oligo2	Dharmacon	D-012200-02	CCACCAAGCUAGAUAAAGA	human
YAP1 oligo3	Dharmacon	D-012200-03	GAACAUAGAAGGAGAGGAG	human
YAP1 oligo4	Dharmacon	D-012200-04	GCACCUAUCACUCUCGAGA	human
MST1 oligo1	Dharmacon	D-004157-01	CCAGAGCUAUGGUCAGAUA	human
MST1 oligo2	Dharmacon	D-004157-02	GUGAAACAGUGUCUUGUAA	human
MST1 oligo3	Dharmacon	D-004157-03	GAUGGGCACUGUCCGAGUA	human
MST1 oligo4	Dharmacon	D-004157-05	GCAGGUCAACUUACAGAUA	human
MST2 oligo1	Dharmacon	D-004874-01	GCCCAUAUGUUGUAAAGUA	human
MST2 oligo2	Dharmacon	D-004874-04	ACAAGUACCUGUUGAAUCA	human
MST2 oligo3	Dharmacon	D-004874-05	CCACAAGCACGAUGAGUGA	human
MST2 oligo4	Dharmacon	D-004874-18	CGGUCAAGUUGUCGCAAUU	human
LATS1 pool	Dharmacon	M063467010005	GCAGAGUACUAGCAAAUUU, GCAGCUGC	mouse
LATS2 pool	Dharmacon	M044602010005	GCGGCAAUAUUUAGACUUU, GAAAUAGC	mouse
TEAD1 pool	Dharmacon	M048419010005	AUAAACCGCUCGCCAAUGU, GGAAAACUU	mouse
TEAD2 pool	Dharmacon	M060552000005	CGAGAGAAAUUCAGUCCAA, UGAUAGAGU	mouse
TEAD3 pool	Dharmacon	M044127010005	GCGAGUACAUCAUCAAUUU, CGACAACG	mouse
TEAD4 pool	Dharmacon	M057322010005	CCAAAUCUAUGACAAGUUC, GAGUACAU	mouse
HRB oligo1	Sigma	SASI_Hs01_00169464	CAGCUUUAGUGGCAGCUUU	human
HRB oligo2	Sigma	SASI_Hs01_00169465	GUCAACAGCUACAGCCAAU	human
HRB oligo3	Sigma	SASI_Hs02_00337763	CCAAAGUCGUGGCAUCAGU	human
ZFP36 oligo1	Sigma	SASI_Hs02_00335603	CCACUUCGCCCACUGCAAC	human
ZFP36 oligo2	Sigma	SASI_Hs01_00030373	CUAUGUCGGACCUUCUCAG	human
ZFP36 oligo3	Sigma	SASI_Hs01_00030376	CGCUACAAGACUGAGCUAU	human
RANBP3 oligo1	Sigma	SASI_Hs01_00176010	GCGAGAAGAGCAUUCGCAU	human
RANBP3 oligo2	Sigma	SASI_Hs01_00176011	CCGAUGACGGCACACUACA	human
RANBP3 oligo3	Sigma	SASI_Hs01_00176012	GACUGAUCCUCAACACCAA	human
NXF3 oligo1	Sigma	SASI_Hs01_00124616	GAUACACUCCCUAUACUAU	human
NXF3 oligo2	Sigma	SASI_Hs02_00355416	CUCUUUGUGCGGGAUACCA	human
NXF3 oligo3	Sigma	SASI_Hs01_00124617	CCUAUACUAUUUCACCCUA	human
THOC3 oligo1	Sigma	SASI_Hs01_00141598	GUGGUUAGUUUGGUCUGUU	human
THOC3 oligo2	Sigma	SASI_Hs01_00141599	GAUAGUGUGGACCAGCUUU	human
THOC3 oligo3	Sigma	SASI_Hs01_00141600	CAGCUUUGUUGGCAUCCAA	human
XPO1 oligo1	Sigma	SASI_Hs01_00084184	CUUAUGGAGGCCCUUCAUU	human
XPO1 oligo2	Sigma	SASI_Hs02_00335588	CAGAUAACCCAAGUCAAAU	human
XPO1 oligo3	Sigma	SASI_Hs01_00084185	CAGCUAUAUUUGCCCAUG	human
Xpo1 oligo1	Sigma	SASI_Mm02_00289246	GCCAAUAUGAGGAACAAUU	mouse
Xpo1 oligo2	Sigma	SASI_Mm02_00289247	GCAUCAAUUCUUGCAUAUA	mouse
Xpo1 oligo3	Sigma	SASI_Mm02_00289248	CAACCAAAUGUUUAUUCAA	mouse
Hrb oligo1	Sigma	SASI_Mm01_00069742	GUGAUCAAGGGAGUGGUUU	mouse
Hrb oligo2	Sigma	SASI_Mm01_00069743	CAGAUAUGGCUAGGAUUAU	mouse
Hrb oligo3	Sigma	SASI_Mm01_00069744	GGGUAAAGCUCCUGUUGGU	mouse
Thoc3 oligo1	Sigma	SASI_Mm02_00334077	GCUGUUUGGACUUCCCAAU	mouse
Thoc3 oligo2	Sigma	SASI_Mm02_00334078	GAAGGACCGGCUGGUCAAA	mouse
Thoc3 oligo3	Sigma	SASI_Mm01_00086842	GAGUUAGUGUGCGUGCGGU	mouse
Zfp36 oligo1	Sigma	SASI_Mm01_00178605	GCUUUGAACUCAAUAUAAU	mouse
Zfp36 oligo2	Sigma	SASI_Mm02_00321352	GUAUGGAUCAGCUAGAUCU	mouse
Zfp36 oligo3	Sigma	SASI_Mm01_00178606	CUGGUCUCCUGGAAUCUUA	mouse
Ranbp3 oligo1	Sigma	SASI_Mm01_00118034	GACUACACAUGCCCAGUCA	mouse
Ranbp3 oligo2	Sigma	SASI_Mm01_00118035	GCAAUGUGCUGCAGAUCCA	mouse
Ranbp3 oligo3	Sigma	SASI_Mm01_00118036	GAUGGAUAAGGCCAGUGAA	mouse
Nxf3 oligo1	Sigma	SASI_Mm01_00191283	GAUCCAAGAUCCUAAGAAU	mouse
Nxf3 oligo2	Sigma	SASI_Mm01_00191284	GAGGAUAUGACCCAGGUGA	mouse
Nxf3 oligo3	Sigma	SASI_Mm01_00191285	CUACUUCUCAGCGAGUUCU	mouse
Xpo1 oligo1	Sigma	SI02746044	CAGGATATTCCTGCTTTCAAA	mouse
Xpo1 oligo2	Sigma	SI02720704	CAGGTTCAAGTTGGAGAAGTA	mouse
Xpo1 oligo3	Sigma	SI02676625	CACATTGAATATGATAAATAA	mouse
Xpo1 oligo4	Sigma	SI00233828	TACGGAAATAATAATGACTAA	mouse

Table S4: Sensitivity analysis of the zero import/export assumption in FRAP model fitting. Related to STAR Methods: Table comparing recovery rates from FRAP experimental data (FRAP), the FLIP approximation to FRAP recovery in the presence (IE) and absence (No IE) of import and export and the ratio of the two FLIP model approximations (IE/(No IE)) for a bleach-point in the centre of the nucleus and right-shifted bleach point.

		Centred bleach			Right-shifted bleach		
Cell	FRAP	IE	No IE	IE/(No IE)	IE	No IE	IE/(No IE)
NF_WT	0.550	0.523	0.547	0.956	0.525	0.541	0.970
NF_5SA	0.300	0.295	0.298	0.990	0.295	0.297	0.993
NF_S94A	0.950	0.818	0.941	0.870	0.850	0.912	0.931
CAF_WT	0.400	0.387	0.399	0.971	0.387	0.394	0.983
CAF_5SA	0.200	0.198	0.200	0.991	0.197	0.198	0.996
CAF_S94A	1.300	1.014	1.283	0.790	1.103	1.225	0.901
