# Supplementary material for: Quantitative Analysis Reveals that Actin and Src-Family Kinases Regulate Nuclear YAP1 and Its Export
Source: Cell Syst. 2018 Jun 27;6(6):692–708.e13. doi: 10.1016/j.cels.2018.05.006 (PMC6035388; doi:10.1016/j.cels.2018.05.006)
Supplement: Data S1. MATLAB FLIP Model Fitting Scripts, Related to STAR Methods — Skeleton MATLAB scripts illustrate the image processing and FLIP PDE nonlinear model fitting code used to analyze FLIP image data. (A) Image processing and PDE model fitting MATLAB script includes example code used to convert the cell to a coarse PDE, extract the spatial intensity profile and nonlinearly fit the system of PDEs to these data. (B) FLIP PDE MATLAB Script demonstrates how to build up a system of PDEs to fit to the experimental data. The full code is available on request. [file mmc14.zip › DataS1.docx]

### Data S1: MATLAB FLIP Model Fitting Scripts related to STAR Methods. Skeleton MATLAB scripts illustrate the image processing and FLIP PDE nonlinear model fitting code used to analyse FLIP image data. (A) Image Processing and PDE Model Fitting MATLAB Script includes example code used to convert the cell to a coarse PDE, extract the spatial intensity profile and nonlinearly fit the system of PDEs to this data. (B) FLIP PDE MATLAB Script demonstrates how to build up a system of PDEs to fit to the experimental data. The full code is available on request.

### (A) Image Processing and PDE Model Fitting MATLAB Script

%Skeleton MATLAB scripts to illustrate the image processing and PDE model
%fitting algorithms used in 'Quantitative analysis reveals that Actin and
%Src-family kinases regulate nuclear YAP1 and its export' by Ege et al.
%Please reference 'Quantitative analysis reveals that Actin and
%Src-family kinases regulate nuclear YAP1 and its export' by Ege et al. if
%using or copying any part of this code


%%%%%%%%%%%%%%%%%%%%%%%%%%%%%%%%%%%%%%%%%%%%%%%%%%%%%%%%%%%%%%%%%%%%%%%%%%%
%<<<<<<<<<<<<<<<<<<<<<<<<<<<<INPUT/OUTPUT>>>>>>>>>>>>>>>>>>>>>>>>>>>>>>>>>>
%%%%%%%%%%%%%%%%%%%%%%%%%%%%%%%%%%%%%%%%%%%%%%%%%%%%%%%%%%%%%%%%%%%%%%%%%%%

%Each cell is coarsly discretized into a grid. The neighbours of each
%coarse gridpoint are saved in an excel file for nlinfitPDEGridSkeleton to
%read.
directoryout='C:\DirectoryOfCellNeighbours\';
neighboursxls=[directoryneighbours 'gridneighbours.xlsx'];

%Location of the lsm file
directory='C:\DirectoryOfCell\';


%Extracting the temporal information. In the full version this excel file
%also contains bleachpoint locations and background and reporting point
%intensities.
directory='C:\DirectoryOfTimeData\';
ExcelRead=[directory 'ReportLocationsAndBackgroundNormalisation.xlsx'];
[num,txt,raw]=xlsread(ExcelRead);
[u,v]=size(num)
TData1=num(:,1);


%Example bleachpoint location and bleachpoint width as input by the user.
%In the full version this is read from an excel file.
Bxedge=140;
Byedge=8;
Bxwidth=290;
Bywidth=8;


%Loading the LSM file.
file_names=[directory '*.lsm'];
files=dir(file_names);
[~, name, extension] = fileparts(files(n).name);
input_name=[directory name extension]
[LSM, varargout] = lsmread(input_name);
%Frame points, T. Size of image u and v.
[T,~,~,u,v]=size(LSM);
%Use the first frame in order to define the nuclear and cytoplasmic
%boundaries
InputFrame(1:u,1:v)=LSM(1,1,1,:,:);

%Image generation to illustrate where the bleachpoint is
LSM1=imadjust(InputFrame,[min(double(InputFrame(:)))/(2^16); ...
 max(double(InputFrame(:)))/(2^16)],[0; 1]);
LSM2=LSM1;
LSM2(Byedge:Byedge+Bywidth-1,Bxedge:Bxedge+Bxwidth-1)=0;
figure
imshow(LSM2)

%Create a binary image of the bleach location
BP1=InputFrame;
BP1=logical(BP1);
BP1(:)=0;
BP1(Byedge:Byedge+Bywidth-1,Bxedge:Bxedge+Bxwidth-1)=1;


%%%%%%%%%%%%%%%%%%%%%%%%%%%%%%%%%%%%%%%%%%%%%%%%%%%%%%%%%%%%%%%%%%%%%%%%%%%
%<<<<<<<<<<<<<<<<<<<<<< COARSE GRID GENERATION>>>>>>>>>>>>>>>>>>>>>>>>>>>>>
%%%%%%%%%%%%%%%%%%%%%%%%%%%%%%%%%%%%%%%%%%%%%%%%%%%%%%%%%%%%%%%%%%%%%%%%%%%

%Based on the location of the bleachpoint, a coarse grid is made covering
%the whole cell. Each grid point has the dimensions of the bleachpoint. A
%single complete gridpoint overlaps exactly with the bleachpoint
XGridStarta=(Bxedge:-Bxwidth:1);
XGridStarta=XGridStarta(end:-1:1);
XGridStartb=(Bxedge+Bxwidth:Bxwidth:v);
XGridStart=[XGridStarta,XGridStartb];
XGridStart=XGridStart';
XGridEnd=XGridStart+Bxwidth-1;
index=find(XGridEnd>v);
XGridStart(index)=[];
XGridEnd(index)=[];

YGridStarta=(Byedge:-Bywidth:1);
YGridStarta=YGridStarta(end:-1:1);
YGridStartb=(Byedge+Bywidth:Bywidth:v);
YGridStart=[YGridStarta,YGridStartb];
YGridStart=YGridStart';
YGridEnd=YGridStart+Bywidth-1;
index=find(YGridEnd>v);
YGridStart(index)=[];
YGridEnd(index)=[];

Gridlabels=zeros(u,v);
counter=0;
for I=1:length(YGridStart)
 for J=1:length(XGridStart)
 counter=counter+1;
 Gridlabels(YGridStart(I):YGridEnd(I),XGridStart(J):XGridEnd(J)) ...
 =counter;
 end;
end;

%%%%%%%%%%%%%%%%%%%%%%%%%%%%%%%%%%%%%%%%%%%%%%%%%%%%%%%%%%%%%%%%%%%%%%%%%%%
%<<<<<<<<<<<<<<<<<<MANUAL NUCLEUS AND CYTOPLASM DETECTION>>>>>>>>>>>>>>>>>>
%%%%%%%%%%%%%%%%%%%%%%%%%%%%%%%%%%%%%%%%%%%%%%%%%%%%%%%%%%%%%%%%%%%%%%%%%%%

%Binary image masks of the nucleus and cytoplasm are generated by the user,
%manually determining the boundaries using roipoly on a heatmap of the
%first frame of the movie.
InputFrame1=InputFrame;
InputFrame1(Byedge:Byedge+Bywidth-1,Bxedge:Bxedge+Bxwidth-1)= ...
 min(InputFrame(:));
close all
figure;imagesc(InputFrame1);colormap(jet)
%Zoom in if necessary. Draw around the cytoplasm.
CytoBoundingRegion=roipoly;
%Zoom in if necessary. Draw around the nucleus.
NucleusBoundingRegion=roipoly;
NucleusIntensityRegion=NucleusBoundingRegion;
%In the full version, nucleoli are also drawn around and removed from the
%object defined as the nucleus (it is assumed protein cannot penetrate such
%dense chromatin).


%%%%%%%%%%%%%%%%%%%%%%%%%%%%%%%%%%%%%%%%%%%%%%%%%%%%%%%%%%%%%%%%%%%%%%%%%%%
%<<<<<<<<<<<<<<<AUTOMATIC CLASSIFICATION OF EACH GRID POINT>>>>>>>>>>>>>>>>
%%%%%%%%%%%%%%%%%%%%%%%%%%%%%%%%%%%%%%%%%%%%%%%%%%%%%%%%%%%%%%%%%%%%%%%%%%%
%Using the binary nuclear and cytoplasmic masks, we automatically determine
%if each coarse grid point is nuclear or cytoplasmic. The gridpoint is
%classified only if over 50% of that gridpoint is occupied by one of the
%two regions. Here we illustrate only the defining of coarse gridpoints in
%the nucleus. The cytoplasm is defined similarly, but has been removed here
%for brevity.

%In NucleusBlockGridlabels each entire coarse gridpoint is uniquely
%labelled to identify that entire gridpoint as a nuclear (generating the
%coarse discretisation).

%In NucleusGrid only the part of the coarse gridpoint that is actually
%defined as the nucleus is labelled (with the same label as
%NucleusBlockGridlabels.) Thus, when calculating the mean intenisty of the
%nucleus at that coarse gridpoint, only parts of the gridpoint that are
%actually nuclear are included in the calculation. Thus gridpoints on the
%nuclear/cytoplasmic boundary only take into account genuine nuclear
%intensity. The equivalent is true when calculating intensities in
%cytoplasmic gridpoints.

NucleusGrid=NucleusIntensityRegion.*Gridlabels;
UnNucleusGrid=unique(NucleusGrid);
UnNucleusGrid(UnNucleusGrid==0)=[];
%If less than half the gridpoint contains the nucleus then the gridpoint is
%deemed not to be part of the nucleus.
for I=1:length(UnNucleusGrid)
 Index=find(NucleusGrid==UnNucleusGrid(I));
 Ratio=length(Index)/(Bxwidth*Bywidth);
 if(Ratio<0.5)
 NucleusGrid(Index)=0;
 end;
end;
UnNucleusGrid=unique(NucleusGrid);
UnNucleusGrid(UnNucleusGrid==0)=[];
NucleusBlockGridlabels=zeros(u,v);


%NucleusGrid is used to calculate the mean intensity of the nucleus at that
%grid point, considering only pixels that form part of the original
%nucleus.
for I=1:length(UnNucleusGrid)
 index=find(Gridlabels==UnNucleusGrid(I));
 NucleusBlockGridlabels(index)=UnNucleusGrid(I);
end;
UnNucleusGrid=sort(UnNucleusGrid);
%Here we set the nucleus box ids from 1 to n
for I=1:length(UnNucleusGrid)
 index=find(NucleusGrid==UnNucleusGrid(I));
 NucleusGrid(index)=I;

 index=find(NucleusBlockGridlabels==UnNucleusGrid(I));
 NucleusBlockGridlabels(index)=I;

end;

%Find the gridlabel for the bleachpoint. This value should be input into
%nlinfitPDEGridSkeleton.m
index=find(BP1);
BleachPointIndex=unique(NucleusGrid(index));
BleachPointIndex=BleachPointIndex(end);

%Do the above in the cytoplasm to label the gridpoints using CytoGrid and
%CytoBlockGridlabels as above with the nucleus.


%%%%%%%%%%%%%%%%%%%%%%%%%%%%%%%%%%%%%%%%%%%%%%%%%%%%%%%%%%%%%%%%%%%%%%%%%%%
%<<<<<<<<<<<<<<<<<<<GRIDPOINT NEIGHBOUR IDENTIFICATION>>>>>>>>>>>>>>>>>>>>>
%%%%%%%%%%%%%%%%%%%%%%%%%%%%%%%%%%%%%%%%%%%%%%%%%%%%%%%%%%%%%%%%%%%%%%%%%%%
%The numerical form of the PDE will change depending on the neighbours of
%each gridpoint (nucleus->nucleus via diffusion, nucleus->cytoplasm via
%import/export and cytoplasm->cytoplasm via diffusion). The neighbours of
%each nuclear and cytoplasmic gridpoint are recorded. For brevity, we
%illustrate only the recording of neighbours to nuclear gridpoints.

unnucblocks=unique(NucleusBlockGridlabels);
unnucblocks(unnucblocks==0)=[];
NucNucNeighbours=zeros(length(unnucblocks),4)-999;
NucCytoNeighbours=zeros(length(unnucblocks),4)-999;
for I=1:length(unnucblocks)
 [N1,N2]=find(NucleusBlockGridlabels==unnucblocks(I));
 temp=[];
 %If there is a nuclear neighbour below, record its label
 if(unique(NucleusBlockGridlabels(max(N1)+1,N2))>0)
 temp=[temp,unique(NucleusBlockGridlabels(max(N1)+1,N2))];
 end;
 %If there is a nuclear neighbour above, record its label
 if(unique(NucleusBlockGridlabels(min(N1)-1,N2))>0)
 temp=[temp,unique(NucleusBlockGridlabels(min(N1)-1,N2))];
 end;
 %If there is a nuclear neighbour right, record its label
 if(unique(NucleusBlockGridlabels(N1,max(N2)+1))>0)
 temp=[temp,unique(NucleusBlockGridlabels(N1,max(N2)+1))];
 end
 %If there is a nuclear neighbour left, record its label
 if(unique(NucleusBlockGridlabels(N1,min(N2)-1))>0)
 temp=[temp,unique(NucleusBlockGridlabels(N1,min(N2)-1))];
 end;
 %Record all the nuclear grid neighbours of that nuclear gridpoint
 NucNucNeighbours(I,1:length(temp))=temp;

 temp=[]; %Similarly, record all the cytoplasmic neighbour gridpoints
 %If there is a cytoplasmic neighbour below, record its label
 if(unique(CytoBlockGridlabels(max(N1)+1,N2))>0)
 temp=[temp,unique(CytoBlockGridlabels(max(N1)+1,N2))];
 end;
 %If there is a cytoplasmic neighbour above, record its label
 if(unique(CytoBlockGridlabels(min(N1)-1,N2))>0)
 temp=[temp,unique(CytoBlockGridlabels(min(N1)-1,N2))];
 end;
 %If there is a cytoplasmic neighbour right, record its label
 if(unique(CytoBlockGridlabels(N1,max(N2)+1))>0)
 temp=[temp,unique(CytoBlockGridlabels(N1,max(N2)+1))];
 end
 %If there is a cytoplasmic neighbour left, record its label
 if(unique(CytoBlockGridlabels(N1,min(N2)-1))>0)
 temp=[temp,unique(CytoBlockGridlabels(N1,min(N2)-1))];
 end;
 NucCytoNeighbours(I,1:length(temp))=temp;
end;

%Record the neighbours of each cytoplasmic gridpoint as above.


%The nuclear and cytoplasmic neighbours for each nuclear and cytoplasmic
%gridpoint are recorded in an excel file. This excel file is read by the
%pde file nlinfitPDEGridSkeleton.m so that it can employ the correct
%interactions between neighbouring gridpoints (e.g. diffusion between
%neighbouring nuclear gridpoints and import export between nuclear
%gridpoints that neighbour cytoplasmic gridpoints.)
xlswrite(neighboursxls,NucNucNeighbours,'NucNucNeighbours');
xlswrite(neighboursxls,NucCytoNeighbours,'NucCytoNeighbours');
xlswrite(neighboursxls,CytoNucNeighbours,'CytoNucNeighbours');
xlswrite(neighboursxls,CytoCytoNeighbours,'CytoCytoNeighbours');


%%%%%%%%%%%%%%%%%%%%%%%%%%%%%%%%%%%%%%%%%%%%%%%%%%%%%%%%%%%%%%%%%%%%%%%%%%%
%<<<<<<<<<<<<<<<<<<<<<DATA FORMATTING FOR nlinfit>>>>>>>>>>>>>>>>>>>>>>>>>>
%%%%%%%%%%%%%%%%%%%%%%%%%%%%%%%%%%%%%%%%%%%%%%%%%%%%%%%%%%%%%%%%%%%%%%%%%%%

%Mean nucleus intensity over time for each gridpoint extracted. The use of
%NucleusGrid ensures only pixel locations that form part of the nucleus are
%included.
clear NucIntenROI
for I=1:T
 clear temp2
 temp2(1:u,1:v)=LSM(I,1,1,:,:);
 for J=1:max(NucleusGrid(:))
 INDEX=find(NucleusGrid(:)==J);
 NucIntenROI(I,J)=mean(temp2(INDEX));
 end;
end;
%Cytoplasmic intensity over time for each gridpoint extracted similarly
%into CytoIntenROI These mean intensities are then used as input data to
%fit our pde model to. Background subtraction and loss of intensity over
%time in the image may be accounted for prior to this. These are taken into
%account in the full version of this code.
FullModelCytoInput=CytoIntenROI;
FullModelNucInput=NucIntenROI;

%Remove timepoints corresponding to the prebleach phase
TData=TData1;
TData(1:2)=[];
FullModelNucInput(1:2,:)=[];
FullModelCytoInput(1:2,:)=[];
TData=TData-TData(1);%Reset first timepoint to zero


%No. of nuclear and cytoplasmic gridpoints
nonuc=max(NucleusGrid(:));
nocyto=max(CytoGrid(:))-max(NucleusGrid(:));

%Generate a matrix of intensities for input into nlinfit for the model to
%fit to
ExpData=[];
for I=1:numel(unnucblocks)
 ExpData=[ExpData;FullModelNucInput(:,I)];
end;
for I=1:numel(uncytoblocks)
 ExpData=[ExpData;FullModelCytoInput(:,I)];
end;

%To carry out the nonlinear curve fitting (nlinfit) requires the intensity
%vector at each gridpoint to have a corresponding time vector.
Tnlinfit = repmat(TData,max(CytoGrid(:)),1);

%%%%%%%%%%%%%%%%%%%%%%%%%%%%%%%%%%%%%%%%%%%%%%%%%%%%%%%%%%%%%%%%%%%%%%%%%%%
%<<<<<<<<<<<<<<<<<<<<<<<<<<RESIDUAL WEIGHTINGS>>>>>>>>>>>>>>>>>>>>>>>>>>>>>
%%%%%%%%%%%%%%%%%%%%%%%%%%%%%%%%%%%%%%%%%%%%%%%%%%%%%%%%%%%%%%%%%%%%%%%%%%%
%Residual weightings affect how the model fits the data. The user may
%choose a wide variety of weightings to the residuals. Here we illustrate
%the approach taken in Ege et al. These could be adapted to suit the user's
%needs/preferences.

%Residuals are time weighted such that events earlier in time have a
%greater weighting than those later. Useful to capture rapid decay due to
%bleaching and diffusion.
W=1./(TData+sum(FullModelCytoInput(:))+sum(FullModelNucInput(:)));
W=repmat(W,max(CytoGrid(:)),1);


%Each gridpoint is then normalised such that each gridpoint has the same
%effect on data fit, regardless of total intenisty in that gridpoint.
for I=1:nonuc
 W((I-1)*length(TData)+1:I*length(TData))=1/sum(...
 FullModelNucInput(:,I))*W((I-1)*length(TData)+1:I*length(TData));
end;
for I=1:nocyto
 W(nonuc*length(TData)+(I-1)*length(TData)+1:nonuc*length(TData)+...
 I*length(TData))=1/sum(FullModelCytoInput(:,I))*...
 W(nonuc*length(TData)+(I-1)*length(TData)+1:nonuc*length(TData)+...
 I*length(TData));
end;

%Gridpoints are then normalised such that sum of residuals of all the
%gridpoints in the nucleus (excluding the bleachpoint), sum of residuals in
%all the gridpoints in the cytoplasm and the sum of residuals in the
%bleachpoint gridpoint all have equal weighting with each other. This
%avoids the nonlinear model fit from fitting badly at the bleachpoint
%because it is outnumbered by cytoplasmic gridpoints and other nuclear
%gridpoints
%Total weight of bleachpoint
BPWeight=sum(W((BleachPointIndex-1)*length(TData)+1:BleachPointIndex*...
 length(TData)))
%Total weight of nucleus minus bleachpoint
RONWeight=sum(W(1:nonuc*length(TData)))-BPWeight
%Total weight of cytoplasm
CWeight=sum(W(nonuc*length(TData)+1:nonuc*length(TData)+nocyto*...
 length(TData)))
W((BleachPointIndex-1)*length(TData)+1:BleachPointIndex*length(TData))=...
 RONWeight/BPWeight*W((BleachPointIndex-1)*length(TData)+1:...
 BleachPointIndex*length(TData));
W(nonuc*length(TData)+1:nonuc*length(TData)+nocyto*length(TData))=...
 RONWeight/CWeight*W(nonuc*length(TData)+1:...
 nonuc*length(TData)+nocyto*length(TData));

%%%%%%%%%%%%%%%%%%%%%%%%%%%%%%%%%%%%%%%%%%%%%%%%%%%%%%%%%%%%%%%%%%%%%%%%%%%
%<<<<<<<<<<<<<<<<<<<<<<PARAMETER GUESS INPUT>>>>>>>>>>>>>>>>>>>>>>>>>>>>>>>
%%%%%%%%%%%%%%%%%%%%%%%%%%%%%%%%%%%%%%%%%%%%%%%%%%%%%%%%%%%%%%%%%%%%%%%%%%%

%Example input parameter guesses. These will change dependent on form of
%assumed pde model.
%In Ege et al. there were additional import/export parameters that were
%also fit.
K1=1; %association rate in nucleus
ETA=1.5; %Decay due to bleaching in the nucleus
C0=mean(FullModelCytoInput(1,:)); %Initial intensity in the cytoplasm
BleachPointIndex %This value must be input into
parguess = [K1 ETA C0];

%PAUSE HERE. IN nlinfitPDEGridSkeleton:
%ADJUST THE DIRECTORY LOCATING THE GRIDNEIGHBOURS EXCEL FILE
%ADJUST THE BLEACHINDEX TO BleachPointIndex.
%ADJUST FIXED PARAMETERS (E.G. DISSOCIATION RATE IN NUCLEUS AND DIFFUSION)
%THEN EXECUTE:
fullmdl=...
 fitnlm(Tnlinfit,ExpData,@nlinfitPDEGridSkeleton,parguess,'Weights',W)

### (B) FLIP PDE MATLAB Script

%Skeleton MATLAB scripts to illustrate the image processing and PDE model
%fitting algorithms used in 'Quantitative analysis reveals that Actin and
%Src-family kinases regulate nuclear YAP1 and its export' by Ege et al.
%Please reference 'Quantitative analysis reveals that Actin and
%Src-family kinases regulate nuclear YAP1 and its export' by Ege et al. if
%using or copying any part of this code

function output = nlinfitPDEGridSkeleton(pars,Tnlinfit)

 %Reads in the gridpoint neighbours such that the correct boundary
 %conditions are imposed at each gridpoint.
 directory='C:\DirectoryOfCellNeighbours\';
 ExcelRead=[directory 'gridneighbours.xlsx'];
 [nucnuc,txt,raw]=xlsread(ExcelRead,'NucNucNeighbours');
 [nuccyto,txt,raw]=xlsread(ExcelRead,'NucCytoNeighbours');
 [cytonuc,txt,raw]=xlsread(ExcelRead,'CytoNucNeighbours');
 [cytocyto,txt,raw]=xlsread(ExcelRead,'CytoCytoNeighbours');

 %Change this to 'BleachpointIndex' output from 'PDEFitSkeleton.m' so
 %model knows where bleach is happening
 bleachid=10;

 [nonuc,~]=size(nucnuc);
 [nocyto,~]=size(cytocyto);
 tspanodedata=Tnlinfit(1:length(Tnlinfit)/(nonuc+nocyto));

 %Example free parameters to be fitted (import/export between
 %compartments and immobile/mobile reactions in the cytoplasm are not
 %included here.)
 k1=pars(1);%Assoc rate in nucleus
 eta=pars(2);%Decay rate due to bleaching
 C0=pars(3);%Initial condition in cytoplasm
 %Example fixed parameters that must be input by the user. The rate of
 %dissociation from immobile to mobile state in the nucleus. Must take a
 %numerical value.
 km1=KM1;
 %The diffusion parameter used in the numerics where D1 is the true rate
 %of diffusion and h accounts for the gridsize in the numerics. Must
 %take a numerical value.
 D=D1/h^2;


 %Example generation of initial conditions: Generate initial conditions
 %for each gridpoint in the nucleus and cytoplasm based on assumed
 %transfer rates between each state and compartment.


 %e.g. initial mobile fraction in nucleus is a function, f, of
 %cytoplasmic initial concentration.
 M0=f(C0);
 %e.g. initial immobile fraction in nucleus is a function of mobile
 %fraction in nucleus and association and dissociation rates.
 I0=abs(k1/km1*M0);
 %Set each gridpoint in nucleus and cytoplasm to these initial
 %conditions.
 for I=1:nonuc;
 z0(2*I-1)=I0;
 z0(2*I)=M0;
 end;
 for I=1:nocyto;
 z0(I+2*nonuc)=C0;
 end;


%PDE reduced to ODE model to be solved using ode solver
 function dz = FlipFullModelODE(t,z)
 %Assume we are in a system where there is a mobile and immobile
 %fraction in the nucleus but the protein is all mobile in the
 %cytoplasm
 dz = zeros(2*nonuc+nocyto,1);
 %Generating odes for nuclear gridpoints
 for I=1:nonuc
 I;
 %Determine the nuclear and cytoplasmic neighbours for each
 %nuclear gridpoint
 nucneighbours=nucnuc(I,:);
 nucneighbours=nucneighbours(nucneighbours>0);
 cytoneighbours=nuccyto(I,:);
 cytoneighbours=cytoneighbours(cytoneighbours>0);

 %If the gridpoint is the bleachpoint we need to include decay
 %due to bleaching (eta).
 if(I==bleachid)
 %ODE for immobile fraction. Incorporates reactions between
 %mobile and immobile states and decay due to bleaching.
 dz(2*I-1)=-abs(km1)*z(2*I-1)+abs(k1)*z(2*I)-eta*z(2*I-1);
 %ODE for mobile fraction. Incorporates transfer between
 %mobile and immobile fractions, decay due to bleaching and
 %diffusion with the rest of the nucleus.
 dz(2*I) = abs(km1)*z(2*I-1)-abs(k1)*z(2*I)...
 +D*sum(z(2*nucneighbours))...
 -D*length(nucneighbours)*z(2*I)-eta*z(2*I);

 else
 %If the gridpoint is elsewhere in the nucleus include
 %conditions for boundaries. I.e. include cases for nuclear
 %only neighbours, nuclear and cytoplasmic neighbours or
 %cytoplasmic only neighbours (caused by coarse
 %discretization of cell). These will determine the
 %inclusion of diffusion and import/export functions etc. in
 %the numerics
 end
 end;


 for I=1:nocyto
 nucneighbours=cytonuc(I,:);
 nucneighbours=nucneighbours(nucneighbours>0);
 cytoneighbours=cytocyto(I,:);
 cytoneighbours=cytoneighbours(cytoneighbours>0);
 %Generate relevant and equivalent odes for each cytoplasmic
 %gridpoint here.

 end;
 end;


%Output of ode solver that nlinfit attempts to fit to the experimental
 %data.
 [T,Z] = ode15s(@FlipFullModelODE,tspanodedata,z0);
 output=[];
 for I=1:nonuc
 output=[output;Z(:,2*I-1)+Z(:,2*I)];
 end;
 for I=1:nocyto
 output=[output;Z(:,2*nonuc+I)];
 end;
end
